# A murine model of hnRNPH2-related neurodevelopmental disorder reveals a mechanism for genetic compensation by *Hnrnph1*

**DOI:** 10.1172/JCI160309

**Published:** 2023-07-17

**Authors:** Ane Korff, Xiaojing Yang, Kevin O’Donovan, Abner Gonzalez, Brett J.W. Teubner, Haruko Nakamura, James Messing, Fen Yang, Alexandre F. Carisey, Yong-Dong Wang, Tushar Patni, Heather Sheppard, Stanislav S. Zakharenko, Yuh Min Chook, J. Paul Taylor, Hong Joo Kim

**Affiliations:** 1 Department of Cell and Molecular Biology, St. Jude Children’s Research Hospital, Memphis, Tennessee, USA.; 2 Department of Pharmacology, University of Texas Southwestern Medical Center, Dallas, Texas, USA.; 3 Department of Developmental Neurobiology,; 4 Department of Biostatistics, and; 5 Department of Pathology, St. Jude Children’s Research Hospital, Memphis, Tennessee, USA.; 6 Howard Hughes Medical Institute, Chevy Chase, Maryland, USA.

**Keywords:** Neuroscience, Neurological disorders

## Abstract

Mutations in *HNRNPH2* cause an X-linked neurodevelopmental disorder with features that include developmental delay, motor function deficits, and seizures. More than 90% of patients with hnRNPH2 have a missense mutation within or adjacent to the nuclear localization signal (NLS) of hnRNPH2. Here, we report that hnRNPH2 NLS mutations caused reduced interaction with the nuclear transport receptor Kapβ2 and resulted in modest cytoplasmic accumulation of hnRNPH2. We generated 2 knockin mouse models with human-equivalent mutations in *Hnrnph2* as well as *Hnrnph2*-KO mice. Knockin mice recapitulated clinical features of the human disorder, including reduced survival in male mice, impaired motor and cognitive functions, and increased susceptibility to audiogenic seizures. In contrast, 2 independent lines of *Hnrnph2*-KO mice showed no detectable phenotypes. Notably, KO mice had upregulated expression of *Hnrnph1*, a paralog of *Hnrnph2*, whereas knockin mice failed to upregulate *Hnrnph1*. Thus, genetic compensation by *Hnrnph1* may counteract the loss of hnRNPH2. These findings suggest that *HNRNPH2*-related disorder may be driven by a toxic gain of function or a complex loss of *HNRNPH2* function with impaired compensation by *HNRNPH1*. The knockin mice described here are an important resource for preclinical studies to assess the therapeutic benefit of gene replacement or knockdown of mutant hnRNPH2.

## Graphical abstract

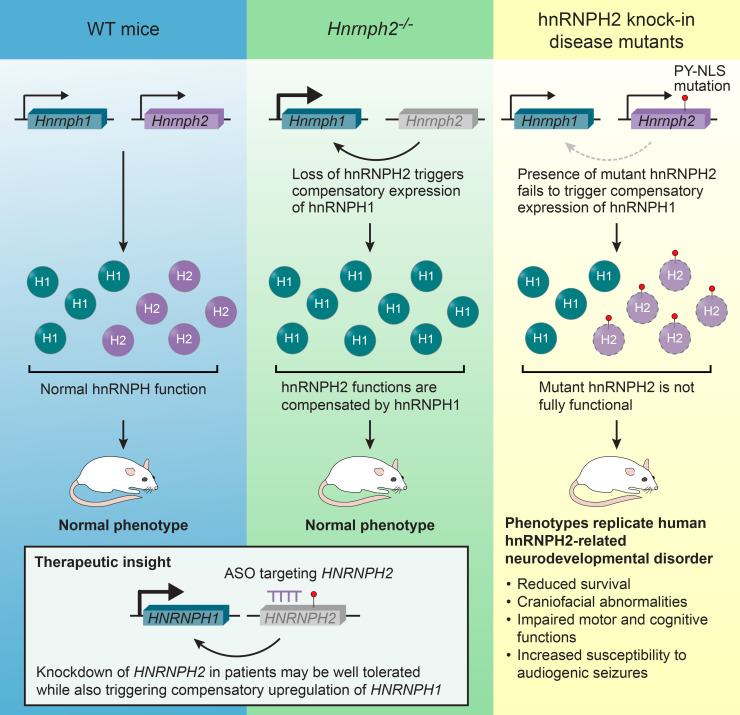

## Introduction

De novo pathogenic variants in *HNRNPH2* were identified in 2016 in 6 unrelated individuals as a novel cause of an X-linked neurodevelopmental disorder, the features of which include developmental delay, intellectual disability, autism spectrum disorder, tone abnormalities, and seizure (OMIM 300986) (1). Since the initial identification of these mutations, the genotypic and phenotypic spectrum of the disorder has been expanded to include more than 30 individuals with 11 distinct de novo variants (2) as well as several maternally inherited cases (3–5). Although all 6 individuals in the initial report were female, subsequent studies have identified male mice carrying missense mutations in *HNRNPH2* associated with a range of overlapping phenotypes (5–7).

More than 90% of individuals with *HNRNPH2*-related neurodevelopmental disorder have a nonsynonymous single nucleotide variant within or adjacent to the nuclear localization signal (NLS) of hnRNPH2, with the 2 most common missense variants, R206W and R206Q, located within the NLS. Additional variants outside the NLS of hnRNPH2 have been reported in children with similar symptoms. Two of these, at residues 114 and 188, are recurrent, suggesting a potential pathogenic effect (4), whereas additional variants found in individual patients are of less clear significance. Individuals with NLS mutations have more severe symptoms than those with variants located outside this region, the latter of which have been reported almost exclusively in male mice (2, 4, 8).

Rare pathogenic variants in *HNRNPH1*, a close paralog of *HNRNPH2*, have been reported in patients with a syndrome very similar to that observed in *HNRNPH2*-related disorder (9, 10). Half of these variants (4 of 8) are located in the NLS of hnRNPH1. As with mutations in hnRNPH2, patients harboring variants within the NLS of hnRNPH1 display a more severe phenotype than patients whose variants are located outside the NLS (9, 10). These observations suggest the possibility of a common basis for abnormal neurodevelopment related to impairment of functions shared between hnRNPH1 and hnRNPH2.

hnRNPH2 is a member of the heterogeneous nuclear ribonucleoprotein (hnRNP) family of proteins, which govern various aspects of nucleic acid metabolism, including transcription, RNA processing, alternative splicing, mRNA trafficking, mRNA stability, and translation (11). hnRNPH2 is a member of the hnRNP H/F subfamily, which comprises hnRNPH1, hnRNPH2, hnRNPH3, and hnRNPF. As components of a messenger ribonucleoprotein complex, hnRNP H/F proteins shuttle between the nucleus and the cytoplasm and function in both compartments. Nucleocytoplasmic transport of hnRNP F/H proteins is regulated by their proline-tyrosine NLS (PY-NLS), which is located in the center of the protein flanked by 2 RNA-recognition motifs (RRMs) (12). In humans, PY-NLSs are recognized for nuclear import by karyopherin β2 (Kapβ2) (13). Deletion of the PY-NLS domain in hnRNPF or mutation of the conserved PY-NLS motif in hnRNPH1 impairs nuclear localization of these proteins (12, 13).

Pathogenic mechanisms arising from variants in *HNRNPH2* remain largely unexamined. One recent in vitro study showed deficiencies in nucleocytoplasmic shuttling of hnRNPH2 with NLS mutations (R206W, P209L), as well as alterations in splicing associated with hnRNPH2 R114W (4). However, detailed characterizations of pathogenic mutations have not been reported, nor have faithful models that recapitulate features of the human clinical syndrome. Mechanistic insight into the functional consequences of syndrome-causing mutations and robust, disease-relevant models are essential for therapeutics development.

Here, we investigated the consequences of common pathogenic mutations within the PY-NLS of *HNRNPH2*. Mutant proteins showed reduced interaction with Kapβ2 and modest but abnormal accumulation in the cytoplasm when expressed in human cells. Knockin mice bearing 2 distinct pathogenic NLS mutations in *Hnrnph2* demonstrated phenotypes highly similar to clinical features observed in humans, including reduced survival in male mice, craniofacial abnormalities, impaired motor and cognitive functions, and increased susceptibility to audiogenic seizures. In contrast, 2 independent *Hnrnph2*-KO mouse models showed no detectable phenotypes, arguing against a simple loss of hnRNPH2 function as the primary driver of disease. Importantly, KO of *HNRNPH2/Hnrnph2* was associated with significant upregulation of the paralogous genes *HNRNPH1/Hnrnph1*, whereas knockin of pathogenic mutations did not result in such upregulation. Thus, our data suggest the possibility of a toxic gain of function or a complex loss of function of hnRNPH2, driven by a failure in compensation by *Hnrnph1*.

## Results

### Pathogenic variants alter the nucleocytoplasmic ratio of hnRNPH2 and enhance its recruitment to stress granules.

**Figure 1:**
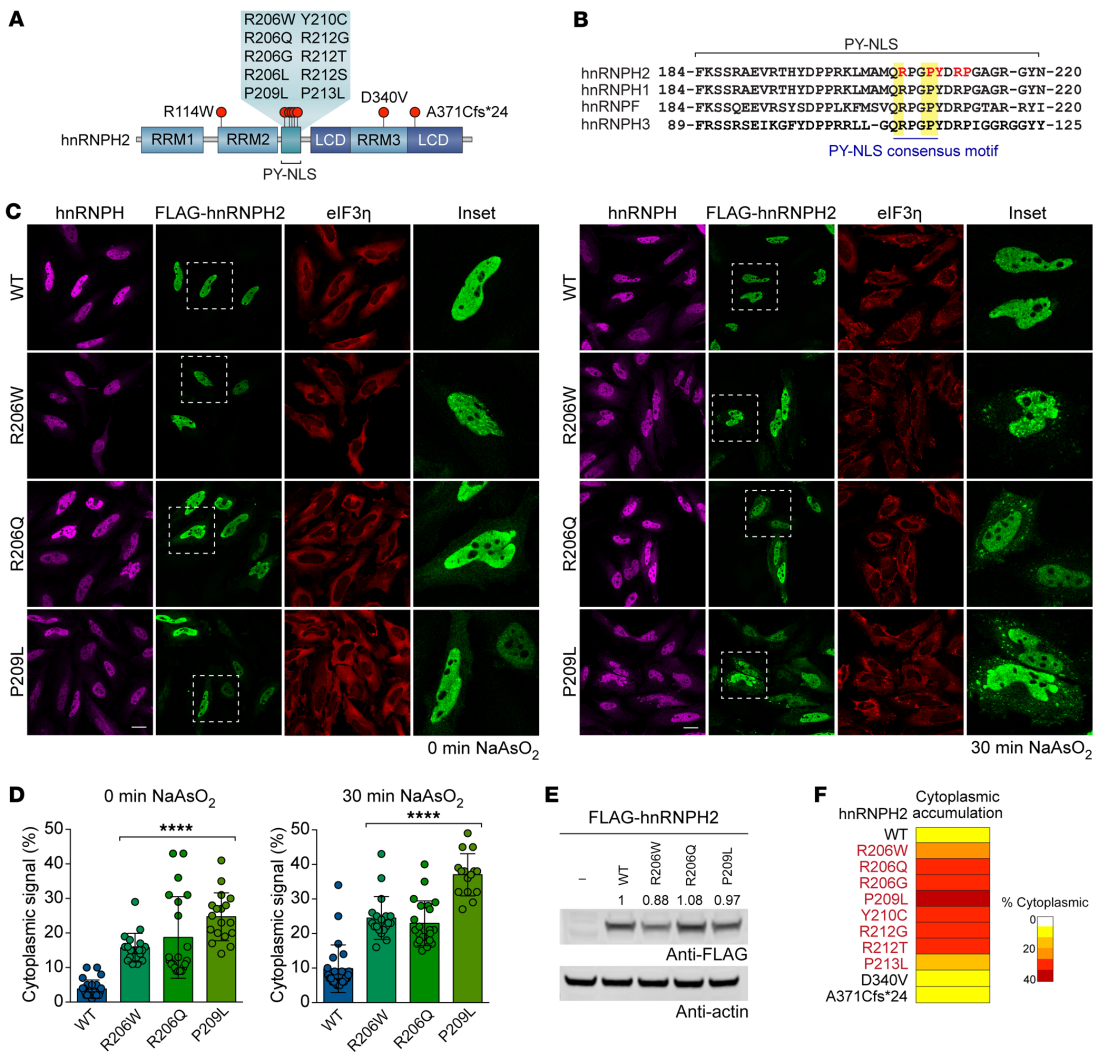
Pathogenic variants alter the nucleocytoplasmic ratio of hnRNPH2 and enhance its recruitment to RNP granules. (**A**) Schematic of hnRNPH2, with red circles indicating mutations in patients with neurodevelopmental disorder. RRM1, RRM2, and RRM3, RNA recognition motifs 1, 2, and 3; PY-NLS, proline-tyrosine nuclear localization signal; LCD, low complexity domain. (**B**) Sequences of human paralogs of the hnRNP F/H family. Yellow indicates consensus PY-NLS motifs; red font indicates patient mutations. (**C**) Intracellular localization of FLAG-hnRNPH2 (WT and indicated mutants) under basal (left) and stressed (right) conditions in HeLa cells. hnRNPH antibody shows localization of endogenous hnRNPH1 and hnRNPH2. eIF3η was used as a cytoplasmic and stress granule marker. The regions within the white boxes are shown at higher magnification in the “Inset” column. Scale bar: 10 μm. (**D**) Quantification of hnRNPH2 cytoplasmic signal intensity as shown in **C**. An interleaved scatterplot with individual data points is shown; data are shown as the mean ± SD. For WT, R206W, R206Q, and P209L mutants, *n* = 24, 19, 21, and 18 cells for basal conditions and *n* = 24, 19, 22, and 15 cells for stressed conditions, respectively. ****P < 0.0001 by 1-way ANOVA with Dunnett’s post test. (**E**) Immunoblot (representative of *n* ≥ 3 experiments) showing comparable levels of expression between hnRNPH2 WT and indicated mutants. Quantified relative expression levels are indicated. (**F**) Summary of intracellular localization of FLAG-hnRNPH2 WT and mutants in HeLa cells, with or without arsenite stress. Images are shown in Supplemental Figure 1. Proteins with PY-NLS mutations (red font) showed cytoplasmic accumulation.

Most hnRNPH2 mutations associated with neurodevelopmental phenotypes are single nucleotide variants located in the PY-NLS of hnRNPH2, which comprises a central hydrophobic or basic motif followed by the motif R/H/K-X_2−5_-PY (Figure 1, A and B). To examine the effect of disease-causing mutations on the subcellular localization of hnRNPH2, we expressed epitope-tagged WT and variant (R206W, R206Q, and P209L) forms of hnRNPH2 in HeLa cells. Under basal conditions, hnRNPH2 WT was almost exclusively located in the nucleus, consistent with established roles for this protein in nuclear RNA processing steps such as splicing. In contrast, disease-causing variants were found in both the nucleus and cytoplasm (Figure 1, C and D). The cytoplasmic accumulation of mutant proteins became more evident when cells were exposed to oxidative stress (0.5 mM NaAsO_2_), which induced the assembly of cytoplasmic stress granules, as marked by eIF3η staining (Figure 1, C and D). All disease-causing mutant forms of hnRNPH2, but not hnRNPH2 WT, were associated with stress granules (Figure 1, C and D). These differences in stress granule association occurred despite comparable levels of hnRNPH2 expression across WT and mutant forms (Figure 1E), suggesting that cytoplasmic accumulation and recruitment of mutant hnRNPH2 to stress granules are not attributable to overexpression of protein. This result is also consistent with those of previous reports that mutations in the PY-NLS of other hnRNPs interfere with their nuclear import and enhance their incorporation into stress granules (14–16).

We next characterized the subcellular localization of 7 additional variants, 5 of which alter the amino acid sequence within the PY-NLS (R206G, Y210C, R212G, R212T, P213L). The remaining 2 variants alter the amino acid sequence in RRM3 (D340V) or the C-terminal low complexity domain (A371Cfs*24), respectively. All hnRNPH2 variants within the PY-NLS showed accumulation of hnRNPH2 protein in the cytoplasm, although the levels of accumulation varied (Figure 1F and Supplemental Figure 1; supplemental material available online with this article; https://doi.org/10.1172/JCI160309DS1). Importantly, despite mutation-dependent redistribution to the cytoplasm, the majority of hnRNPH2 was still found in the nucleus. Indeed, even with the mutation (P209L) that caused the greatest amount of cytoplasmic accumulation, we estimated that approximately 75% of hnRNPH2 was found in the nucleus. In contrast, the 2 non–PY-NLS variants did not show cytoplasmic accumulation of hnRNPH2 (Figure 1F and Supplemental Figure 1); thus, cytoplasmic redistribution of hnRNPH2 is not an invariant feature of the neurodevelopmental syndrome. We note that these 2 non–PY-NLS variants were found in male patients, who are hemizygous and therefore express only mutant hnRNPH2, in contrast to heterozygous female patients who express a mix of WT and mutant protein.

### Pathogenic variants impair the interaction between hnRNPH2 and its nuclear transport receptor Kapβ2.

**Figure 2:**
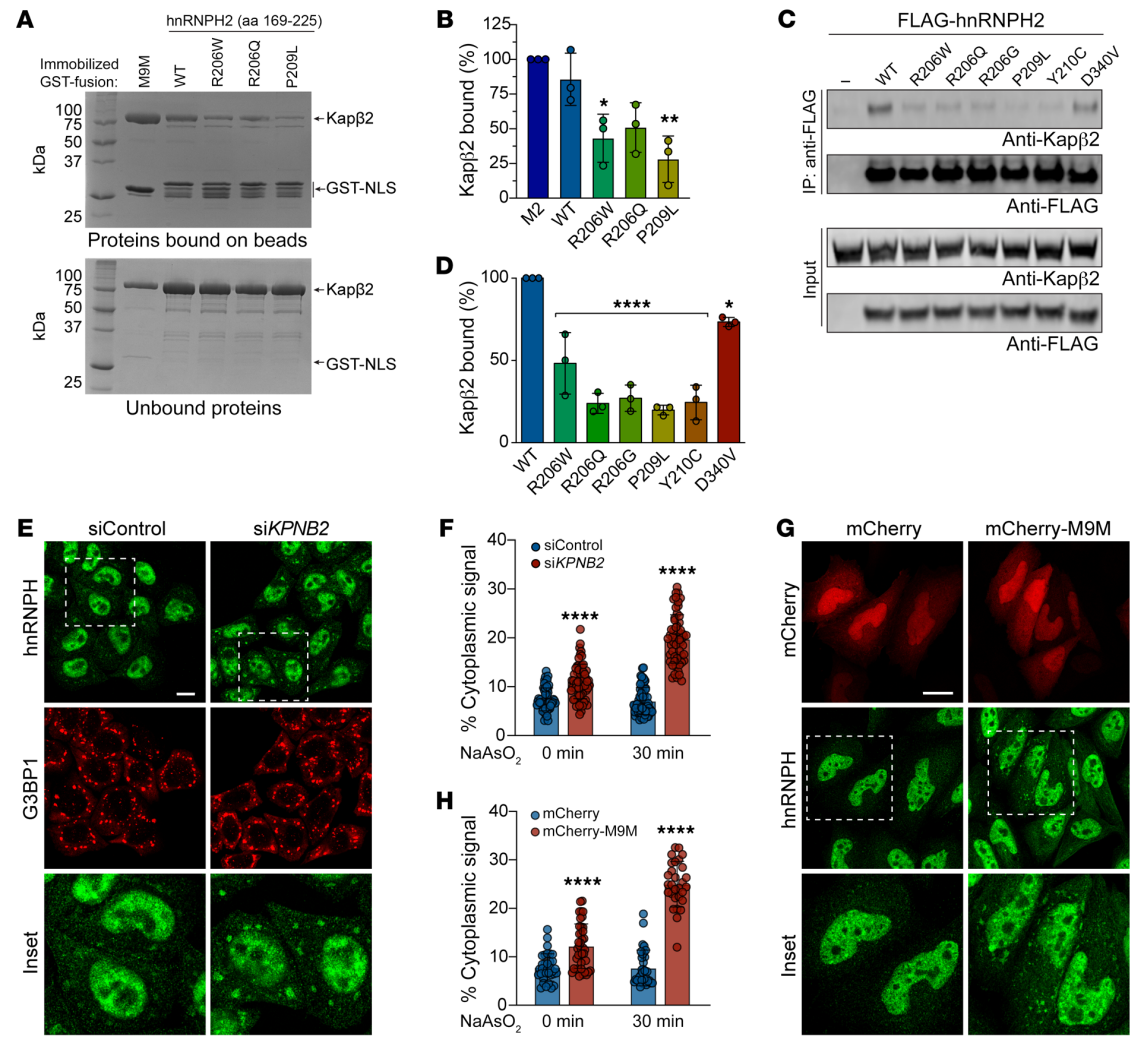
Pathogenic variants impair the interaction between hnRNPH2 and Kapβ2. (**A**) GST pulldown of purified GST-hnRNPH2 peptides with Kapβ2. Proteins bound and unbound to GST beads were visualized by Coomassie Blue. (**B**) Relative band intensities of bound Kapβ2 in triplicate experiments. *n* = 3 biological repeats. (**C**) HEK293T cells were transfected with indicated FLAG-hnRNPH2 constructs, immunoprecipitated for FLAG, and immunoblotted. (**D**) Densitometric analysis of hnRNPH2 and Kapβ2 interaction from immunoblots as shown in **C**. *n* = 3 biological repeats. (**E**) Fluorescent staining of HeLa cells transfected with nontargeting siRNA (siControl) or siRNA targeting *KPNB2*. Transfected cells were treated with sodium arsenite for 30 minutes, fixed, and stained with indicated antibodies. G3BP1 was used as a stress granule marker. The regions within the white boxes are shown at higher magnification in the “Inset” column. Scale bar: 10 μm. (**F**) Quantification of hnRNPH2 cytoplasmic signal intensity in HeLa cells as shown in **E**. An interleaved scatterplot with individual data points is shown. For siControl and si*KPNB2*, *n* = 56 and 62 cells for basal conditions, *n* = 63 and 57 cells for stressed conditions, respectively. (**G**) HeLa cells transfected with mCherry or mCherry-M9M were stained with indicated antibodies. Scale bar: 10 μm. The regions within the white boxes are shown at higher magnification in the “Inset” column. (**H**) Quantification of hnRNPH2 cytoplasmic signal intensity in HeLa cells as shown in **G**. An interleaved scatterplot with individual data points is shown. For mCherry and mCherry-M9M, *n* = 34 and 40 cells for basal conditions and *n* = 33 and 29 cells for stressed conditions, respectively. Data are shown as the mean ± SD. *P < 0.05, **P < 0.01, ****P < 0.0001 by 1-way ANOVA with Dunnett’s post test (**B** and **D**) or 2-way ANOVA with Šidák’s post test (**F** and **H**). Where applicable, exact *P* values are provided in Supplemental Table 4.

Closely paralogous proteins hnRNPH1 and hnRNPF interact with the nuclear import receptor Kapβ2 via their PY-NLS (12, 13, 17). Given the high degree of identity among hnRNPH1, hnRNPF, and hnRNPH2 (Figure 1B), we predicted that hnRNPH2 would bind to Kapβ2 via its PY-NLS for nuclear import and that disease-causing mutations in the PY-NLS would alter this interaction. To test this hypothesis, we performed GST pulldown assays of GST-tagged WT and mutant (R206W, R206Q, and P209L) hnRNPH2 PY-NLS (aa 179–215) (Supplemental Figure 2, A and B). As a positive control, we used an M9M peptide designed to bind to the PY-NLS binding site of Kapβ2 with an affinity that is approximately 200-fold stronger than a natural PY-NLS (18). We observed pulldown of Kapβ2 with GST-M9M and GST-hnRNPH2 WT peptide but not with mutant peptides (Supplemental Figure 2B). When we increased the length of the N- and C-terminal flanking sequences included in the PY-NLS peptide (aa 169–225), the interaction between Kapβ2 and GST-hnRNPH2 PY-NLS peptide was greatly enhanced (Supplemental Figure 2C). Indeed, this form of GST-hnRNPH2 PY-NLS WT (aa 169–225) bound Kapβ2 as efficiently as GST-M9M (Supplemental Figure 2C). However, the disease-associated mutant peptides showed reduced interaction with Kapβ2, even when expressed with this larger flanking sequence (Figure 2, A and B, and Supplemental Figure 2C). The degree to which the PY-NLS mutations impaired Kapβ2 binding correlated with the degree of cytoplasmic redistribution observed in cells, with P209L having the greatest effect (Figure 1, C–F, and Figure 2, A and B).

We next expressed full-length hnRNPH2 constructs in cells to test their interaction with Kapβ2 via immunoprecipitation. Consistent with our GST pulldowns, full-length hnRNPH2 WT coimmunoprecipitated efficiently with Kapβ2, whereas all disease-associated mutant proteins showed reduced interaction with Kapβ2 (Figure 2, C and D). Notably, PY-NLS mutants (R206W/Q/G, P209L, and Y210C) showed a far greater reduction in Kapβ2 interaction (~50%–75%) compared with the non–PY-NLS mutant D340V (Figure 2, C and D).

To test the functional consequences of the hnRNPH2-Kapβ2 interaction, we next inhibited Kapβ2 by RNAi-mediated knockdown (Supplemental Figure 2, D and E). Expression of siRNA targeting *KPNB2* (also known as *TNPO1*) resulted in an approximately 90% decrease in Kapβ2 protein levels (Supplemental Figure 2, D and E) accompanied by increased cytoplasmic localization of endogenous hnRNPH protein and increased association of hnRNPH with stress granules, as assessed by staining with the stress granule marker G3BP1 (Figure 2, E and F). We observed a similar result with overexpression of mCherry-M9M peptide, which caused endogenous hnRNPH to accumulate in the cytoplasm and form cytoplasmic puncta (Figure 2, G and H). These results support the hypothesis that disease-associated PY-NLS mutations impair the ability of hnRNPH2 to bind Kapβ2, thereby diminishing nuclear import of hnRNPH2 and leading to cytoplasmic accumulation.

### hnRNPH2 P209L and R206W mice, but not KO mice, have reduced survival and body weight.

**Figure 3:**
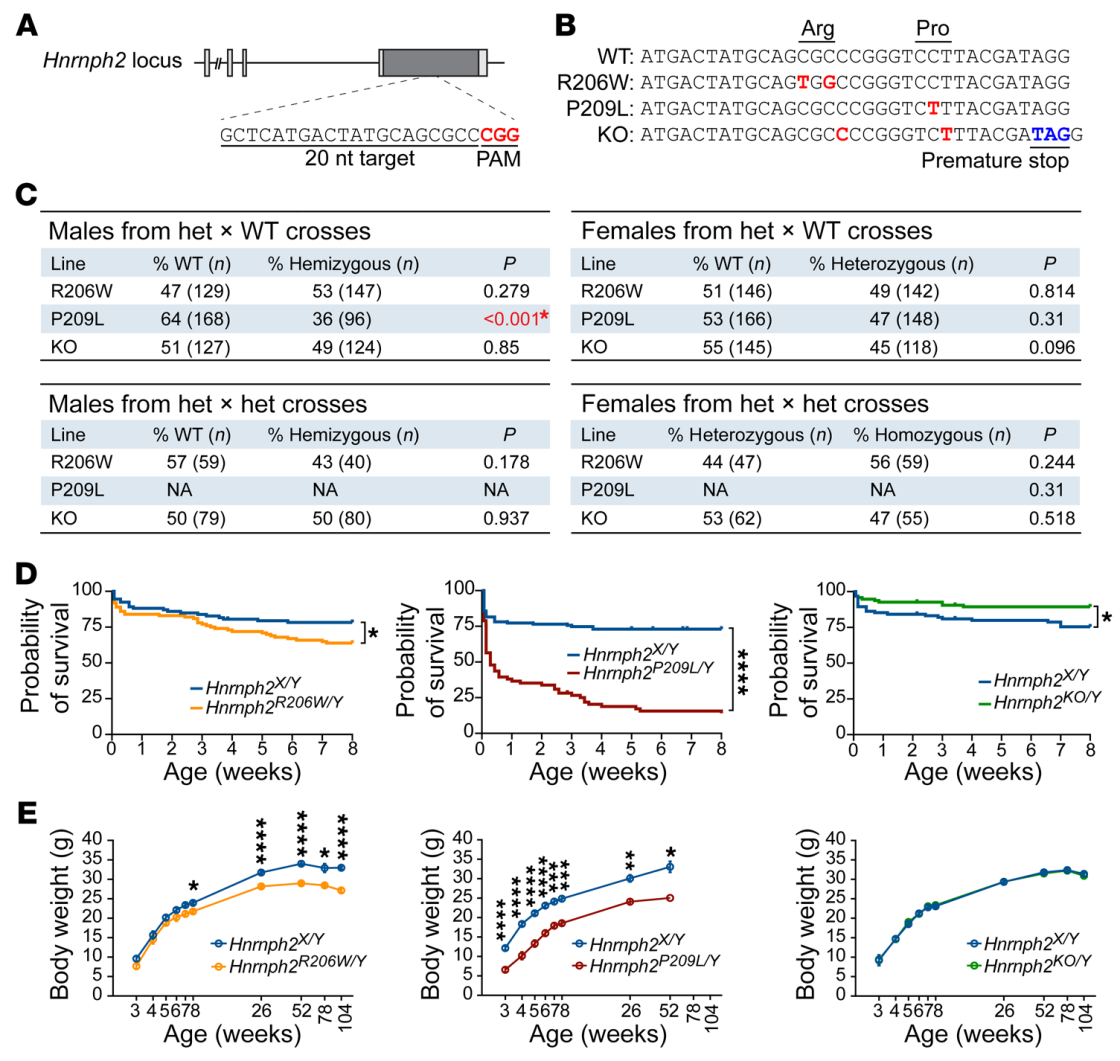
Generation, survival, and body weight of *Hnrnph2*-mutant and -KO mice. (**A**) Schematic of the *Hnrnph2* locus. sgRNA target sequence is shown; red text indicates protospacer adjacent motif (PAM). (**B**) Editing induced by sgRNA and single-stranded oligo donor in mouse. Red text indicates edited nucleotide sequences; blue text indicates a premature stop codon introduced by an indel. (**C**) Ratios of genotyped mice. Significant *P* values shown in red (χ^2^ test). (**D**) Kaplan-Meier survival curves of male mice up to 8 weeks of age; HR = 1.764 for *Hnrnph2^R206W/Y^* (*n* = 100) vs. *Hnrnph2^X/Y^* (*n* = 93); HR = 4.363 for *Hnrnph2^P209L/Y^* (*n* = 71) vs. *Hnrnph2^X/Y^* (*n* = 119); HR = 0.4111 for *Hnrnph2^KO/Y^* (*n* = 95) vs. *Hnrnph2^X/Y^* (*n* = 95). (**E**) Mean body weight of male mice over time. Data are shown as the mean ± SEM. *Hnrnph2^R206W/Y^* (*n* = 13) vs. *Hnrnph2^X/Y^* (*n* = 13); *Hnrnph2^P209L/Y^* (*n* = 12) vs. *Hnrnph2^X/Y^* (*n* = 7); NS for *Hnrnph2^KO/Y^* (*n* = 18) vs. *Hnrnph2^X/Y^* (*n* = 10). Two *Hnrnph2* KO lines (KOMP KO and indel KO2) were used for all analyses, and both lines showed the same results. For simplicity, data from our indel-based KO line are included in each graph. Data from the KOMP-KO line are summarized in Supplemental Figure 5. *P < 0.05, **P < 0.01, ***P < 0.001, ****P < 0.0001 by Mantel-Cox test (**D**) or mixed-effects model (REML) with Šidák’s post test (**E**). Where applicable, exact *P* values are provided in Supplemental Table 4.

Like human *HNRNPH2*, the mouse *Hnrnph2* gene is located on the X chromosome. Human hnRNPH2 and mouse hnRNPH2 have 99% identity; both are 449 amino acids in length, with conservative differences in the identity of only 4 amino acids (<1%), and the PY-NLS motif is perfectly conserved between the two species (Supplemental Figure 3). To investigate the effects of mutations on normal hnRNPH2 function and to model the pathogenicity of mutant hnRNPH2, we generated mouse models by homologous knockin of the human hnRNPH2 R206W or P209L mutations into mouse *Hnrnph2* (Figure 3, A and B, and Supplemental Figure 4A). While generating these knockin mouse lines, we also serendipitously obtained a KO line in which a frameshift caused by an indel generated a premature stop codon, leading to nonsense-mediated mRNA decay (Figure 3B and Supplemental Figure 4, B–D). We also obtained a second KO line from the Knockout Mouse Project (KOMP) Repository (C57BL/6NJ-*^Hnrnph2em1(IMPC)J^*/MmJax, The Jackson Laboratory). These two KO lines differ in two respects: first, in contrast to our KO line, which was driven by an indel with consequent nonsense-mediated decay, the KOMP-KO line was generated by a 1,451 bp deletion in exon 4, which was predicted to result in a truncated, nonfunctional transcript. Second, the background strain of our *Hnrnph2*-mutant and -KO lines is C57BL/6J, whereas the KOMP KO is on a C57BL/6NJ background. Thus, we selected 2 distinct disease-associated *Hnrnph2* knockin mouse lines (R206W and P209L) and 2 distinct *Hnrnph2* KO lines for in-depth phenotypic analysis. Importantly, extensive phenotypic analysis of our indel-based KO line and the KOMP-KO line yielded equivalent results. For ease of presentation, we include data from our own indel-based KO line (hereafter referred to as KO) in all subsequent figures alongside data from our knockin lines, whereas all results from the KOMP-KO line are compiled in Supplemental Figure 5.

All lines produced viable offspring. All heterozygous female mice were born with expected frequencies. In contrast, hemizygous P209L mutant male mice, but not R206W and KO male mice, were detected at a lower frequency than predicted by Mendelian laws (64% WT vs. 36% *Hnrnph2^P209L/Y^*), suggesting partial embryonic lethality of male mice bearing the P209L mutation (Figure 3C). We also crossed heterozygous mutant female mice to hemizygous mutant male mice to produce homozygous mutant mice. Again, homozygous female mice from the R206W and KO lines were born at close to expected frequencies (Figure 3C). We note that this experiment could not be performed in the P209L line, as most hemizygous P209L male mice did not survive long enough to breed. Indeed, less than 15% of *Hnrnph2^P209L/Y^* male mice lived to 8 weeks of age (Figure 3D). *Hnrnph2^R206W/Y^* male mice also had significantly reduced survival up to 8 weeks of age (Figure 3D). In contrast, heterozygous female mice from all lines showed no significant changes in survival up to 8 weeks of age (Supplemental Figure 6A), and hemizygous KO male mice had slightly better survival rates than their littermate controls (Figure 3D). Homozygous female mice from R206W and KO lines also did not have significantly reduced survival up to 8 weeks, although there was a trend toward reduced survival in *Hnrnph2^R206W/R206W^* female mice compared with *Hnrnph2^R206W/X^* female littermates (Supplemental Figure 6B). These results suggest a dosage-dependent effect of *Hnrnph2* mutation on survival and indicate that the P209L mutation has a more severe effect on survival than R206W, consistent with their respective effects in vitro and in cell lines (Figures 1 and 2).

To investigate the effects of hnRNPH2 mutations on long-term survival, we monitored a subset of mice for up to 2 years. In this smaller cohort, we found no significant difference in long-term survival between hemizygous male mice or heterozygous female mice and WT littermate controls (Supplemental Figure 6C). *Hnrnph2^P209L/Y^* and *Hnrnph2^R206W/Y^* male mice weighed significantly less than their WT littermate controls (Figure 3E). *Hnrnph2^R206W/X^* female mice also had significantly reduced body weight compared with littermate controls (Supplemental Figure 6D). *Hnrnph2^P209L/X^* female mice tended to weigh less than littermates, but the difference was not significant (Supplemental Figure 6D). Once more, neither male nor female KO mice were significantly different from controls in terms of body weight or long-term survival, suggesting that reduced survival or reduced body weight in knockin mice likely does not arise from simple loss of hnRNPH2 function.

### hnRNPH2 P209L and R206W mice, but not KO mice, have craniofacial abnormalities and increased incidence of hydrocephalus.

**Figure 4:**
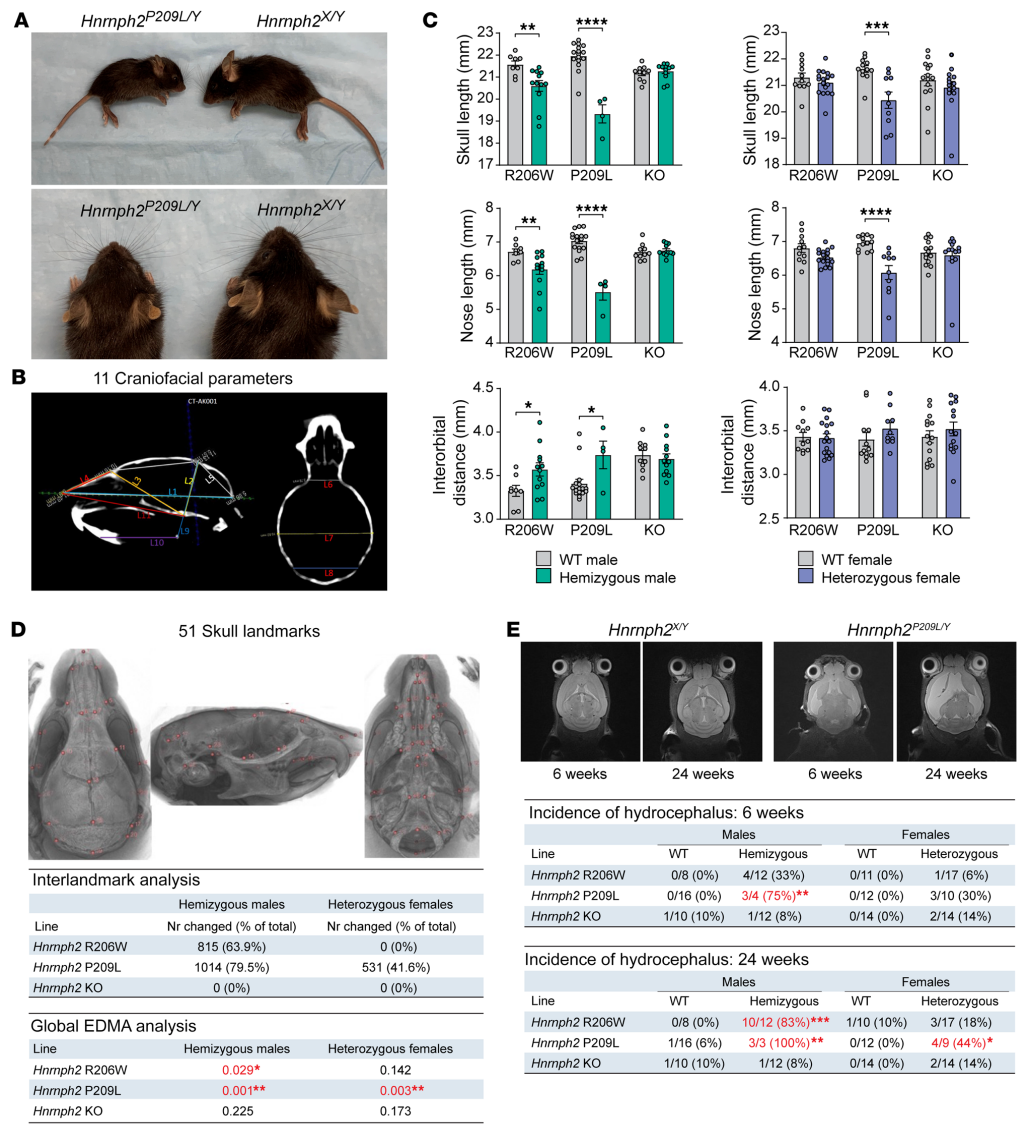
hnRNPH2-mutant mice have craniofacial dysmorphology and increased incidence of hydrocephalus. (**A**) Images of a representative 3-week-old male *Hnrnph2^P209L/Y^* mouse and WT littermate. (**B**) Craniofacial parameters measured manually on individual μCT scans. (**C**) Linear measurements in mice; data are shown as the mean ± SEM. Group sizes for μCT and MRI: *Hnrnph2^R206W/Y^* (*n* = 12) vs. *Hnrnph2^X/Y^* (*n* = 8); *Hnrnph2^P209L/Y^* (*n* = 4) vs. *Hnrnph2^X/Y^* (*n* = 16); *Hnrnph2^KO/Y^* (*n* = 12) vs. *Hnrnph2^X/Y^* (*n* = 10); *Hnrnph2^R206W/X^* (*n* = 17) vs. *Hnrnph2^X/X^* (*n* = 11); *Hnrnph2^P209L/X^* (*n* = 10) vs. *Hnrnph2^X/X^* (*n* = 12); *Hnrnph2^KO/X^* (*n* = 14) vs. *Hnrnph2^X/X^* (*n* = 14). (**D**) Location of 51 landmarks on mouse skull atlas, number of significantly changed linear interlandmark distances, and results of global EDMA analysis. Significant *P* values are shown in red. (**E**) Representative MRI images showing hydrocephalus in a *Hnrnph2^P209L/Y^* hemizygous male mouse compared with a WT littermate and incidence of hydrocephalus at 6 and 24 weeks of age. *P < 0.05, **P < 0.01, ***P < 0.001, ****P < 0.0001 by 2-way ANOVA with Šidák’s post test (**C**), EDMA bootstrap global test (**D**), or Fisher’s exact test (**E**). Where applicable, exact *P* values are provided in Supplemental Table 4.

All human patients with hnRNPH2-related phenotypes have dysmorphic facial features (1). Although most of these patients have unremarkable MRIs, some do present with vertical configuration of the splenium of the corpus callosum, delayed myelination, and decreased cerebellar volume (2). During initial breeding of founders to WT mice, we noticed that in addition to being smaller overall, *Hnrnph2^P209L/Y^* male mice, and to a lesser extent *Hnrnph2^R206W/Y^* male mice, appeared to have short snouts and wide-set eyes (Figure 4A). To further investigate this phenotype, we performed in vivo μCT and MRI on a cohort of mutant knockin and KO mice at 6 and 24 weeks of age.

Manual linear measurements of 11 key craniofacial parameters (19) revealed significant reduction in skull and nose length in *Hnrnph2^P209L/Y^*, *Hnrnph2^R206W/Y^*, and *Hnrnph2^P209L/X^* mice at 6 weeks of age as well as a significant increase in interorbital distance in *Hnrnph2^P209L/Y^* and *Hnrnph2^R206W/Y^* male mice (Figure 4, B and C). Furthermore, upper jaw length was significantly reduced in *Hnrnph2^P209L/Y^*, *Hnrnph2^R206W/Y^*, and *Hnrnph2^P209L/X^* mice, in addition to a reduction in lower jaw length in *Hnrnph2^P209L/Y^* male mice (Supplemental Figure 7A). Importantly, no changes were seen in *Hnrnph2*-KO mice (Figure 4C and Supplemental Figure 7A). To investigate this craniofacial phenotype more extensively, we used a population-level atlas for the automatic identification of 51 skull landmarks (20), followed by pairwise comparison between all landmarks (Figure 4D). After correction for multiple comparisons, *Hnrnph2^P209L/Y^*, *Hnrnph2^R206W/Y^*, and *Hnrnph2^P209L/X^* mice had many significant changes in interlandmark distances, which were mostly decreased compared with those of littermate controls, whereas KO mice showed no differences (Figure 4D). As these measures were not normalized, we were concerned that the observed changes were due to a reduction in the overall size of the mutant mice and not a change in craniofacial shape. To address this, we performed Euclidean distance matrix analysis (EDMA) on 3D landmark coordinate data scaled to centroid size (21, 22). EDMA based on all 51 landmarks revealed a significant difference in global skull shape of *Hnrnph2^P209L/Y^*, *Hnrnph2^R206W/Y^*, and *Hnrnph2^P209L/X^* mice but not *Hnrnph2-*KO mice (Figure 4D). EDMA based on subsets of biologically relevant landmarks (23) showed significant changes in several anatomical regions of *Hnrnph2^P209L/Y^*, *Hnrnph2^R206W/Y^*, *Hnrnph2^P209L/X^* mice and, to a lesser extent, *Hnrnph2^R206W/X^* female mice (Supplemental Figure 7B). In this analysis, the only changes detected in *Hnrnph2*-KO mice were slight but significant differences in the neural crest-mesoderm boundary (Supplemental Figure 7B). Results at 24 weeks of age did not differ significantly from those at 6 weeks (data not shown).

hnRNPH2 P209L and R206W mice often had domed heads, typically associated with hydrocephalus that develops before ossification of the cranial sutures. Although the C57BL/6J background has a relatively high incidence of hydrocephalus (0.029% at The Jackson Laboratory), the number of mice with pathologically confirmed hydrocephalus suggested an increased incidence in hnRNPH2 P209L and R206W mutant mice, but not in KO mice, compared with WT controls (Supplemental Figure 7C). The cause of hydrocephalus in these mice is unknown, as we found neither evidence of aqueduct blockage (Supplemental Figure 7D), nor abnormal morphology of cilia on ependymal cells lining the dilated ventricles, nor motile ciliary dysfunction in the respiratory system (data not shown) (24). For a more quantitative measurement, we scored mice in the MRI cohort as moderately, highly, or severely hydrocephalic. Significantly more 6-week-old *Hnrnph2^P209L/Y^* mice as well as 24-week-old *Hnrnph2^P209L/Y^*, *Hnrnph2^R206W/Y^*, and *Hnrnph2^P209L/X^* mice had at least moderate hydrocephalus compared with WT littermates (Figure 4E and Supplemental Figure 7E). Neither male nor female *Hnrnph2*-KO mice showed increased incidence of hydrocephalus compared with WT littermates (Figure 4E and Supplemental Figure 7C). Notably, hydrocephalic mice in this cohort did not have obvious doming of the skull, suggesting onset of hydrocephalus after closure of cranial sutures in this group. In agreement with histology, no evidence of aqueduct blockage was detected on MRI (data not shown). Given the MRI abnormalities observed in some patients with hnRNPH2 mutations, we performed automated brain parcellation and volumetrics to investigate group differences in total and regional brain volumes. Automated alignment of MRI images to the DSURQE atlas (25) revealed a small but significant decrease in total brain tissue volume (gray matter plus white matter) in 6-week-old *Hnrnph2^P209L/X^* female mice, most likely attributable to their small body size. This difference was lost when calculating total intracranial volume by adding CSF volume to the total tissue volume (brain tissue volume plus CSF). We did not observe any changes in hnRNPH2 R206W or KO mice (Supplemental Figure 7F). We note that this analysis could not be performed in *Hnrnph2^P209L/Y^* male mice due to the low number of mutant male mice surviving to 6 weeks and the failure of these mice to pass atlas alignment quality control in all but 1 case. No significant differences were detected in any of the 356 cortical, white matter, subcortical, or CSF defined regions when normalized to total brain tissue volume, suggesting that brain growth was relatively preserved in mutant mice (Supplemental Table 1).

### Neurons cultured from hnRNPH2 P209L mice show defects in dendritic arborization of cortical neurons.

To characterize developmental abnormalities in the central nervous system of the mutant mice, we performed a systematic histological analysis of hemizygous male mutant or KO mice and their WT littermates. H&E staining revealed no gross abnormalities in the brains of hnRNPH2 P209L, R206W, or KO male mice (Supplemental Figure 8A), apart from the presence of varying degrees of dilatation of the ventricles. Furthermore, immunohistochemistry with markers for astrocytes (GFAP) and microglia (IBA1) did not reveal evidence of inflammation in hnRNPH2 P209L, R206W or KO male brains compared with those of their WT littermate controls (Supplemental Figure 8, B and C). In addition, Luxol fast blue staining and immunohistochemistry using OLIG2, a specific and universal marker of oligodendrocytes in the brain, did not reveal any changes in central nervous system myelination (Supplemental Figure 8, B and D). Finally, immunohistochemistry using the neuronal marker NeuN and immunofluorescence with cortical layer–specific markers SATB2 (layer II–IV), CTIP2 (layer V), and FOXP2 (layer VI) revealed neither significant cell loss nor altered lamination in the visual, somatosensory, or somatomotor cortex (Supplemental Figure 9).

**Figure 5:**
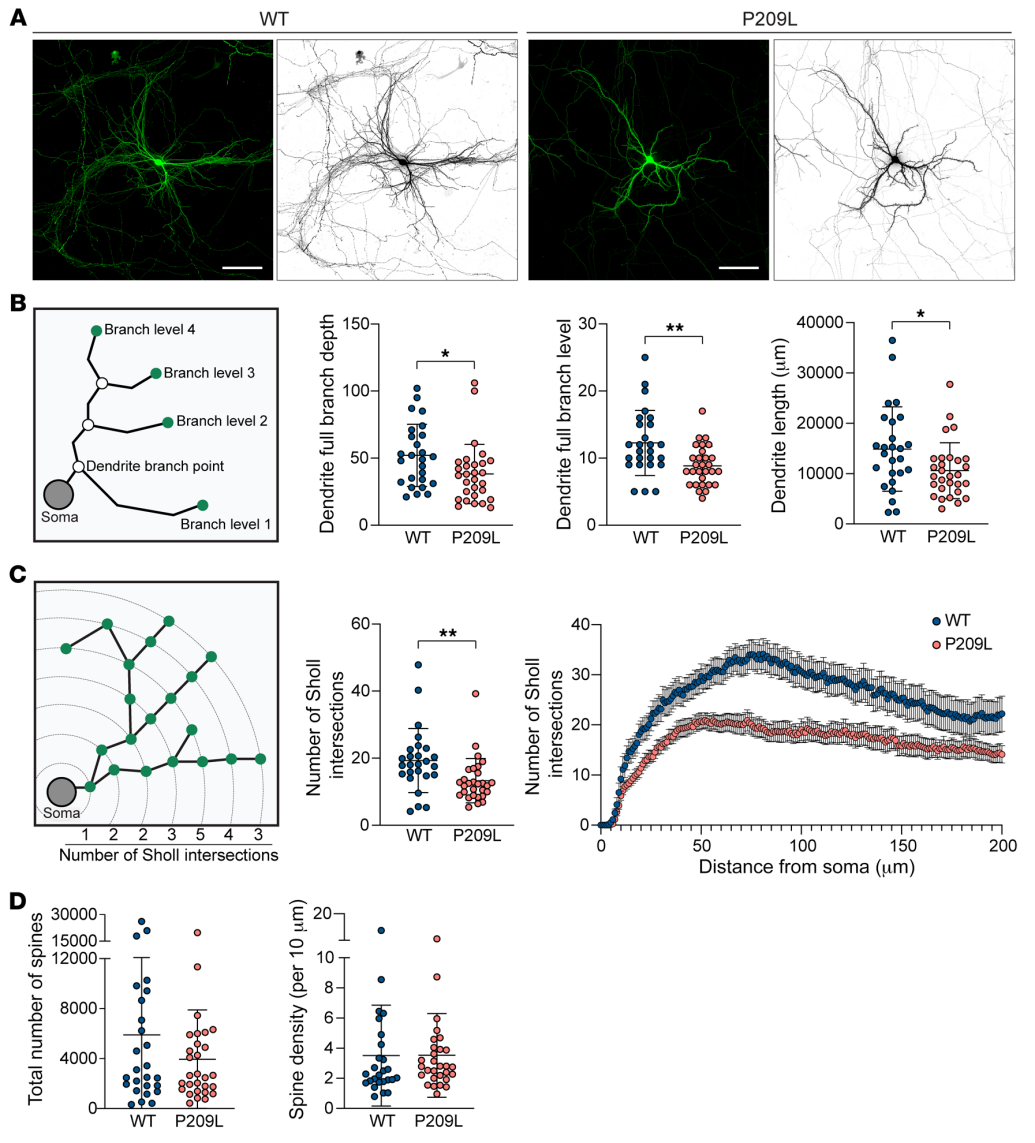
Pathogenic mutation of hnRNPH2 impairs dendritic arborization of cortical neurons. (**A**) Representative micrographs of cortical neurons isolated from WT and *Hnrnph2^P209L/Y^* mice. Cortical cells at P0 from *Hnrnph2^P209L/Y^* and littermate control mice were transfected with GFP at 7 days in vitro, and dendritic morphology was examined at 14 days in vitro. Scale bar: 100 μm. (**B**) Diagram of morphometric measurements and quantified data. Data are shown as the mean ± SD. (**C**) Sholl analysis of neurons isolated from WT and *Hnrnph2^P209L/Y^* mice. The average number of Sholl intersections across the entire neuron (data are shown as the mean ± SD) and the average number of Sholl intersections with 1 μm intervals (data are shown as the mean ± SEM) are plotted. (**D** and **E**) Quantification of total number of dendritic spines across the entire cortical neuron (**D**) and average density of dendritic spines per 10 μm distance along dendrites (**E**). Data are shown as the mean ± SD. Data represent *n* = 26 WT and *n* = 29 P209L neurons. *P < 0.05, **P < 0.01, by unpaired *t* test. Where applicable, exact *P* values are provided in Supplemental Table 4.

To further examine the effect of pathogenic mutations on neuronal development at the cellular level, we assessed dendritic arborization and spine densities in neurons cultured from *Hnrnph2^P209L/Y^* mice. Compared with neurons cultured from littermate control mice, neurons cultured from *Hnrnph2^P209L/Y^* mice had a dramatic reduction in the dendritic arbor (Figure 5A). We detected significantly reduced dendrite branch points, dendrite branch levels, and total dendritic lengths in neurons cultured from *Hnrnph2^P209L/Y^* mice (Figure 5B) that led to a large reduction in dendritic arbor complexity, as assessed by Sholl analysis (Figure 5C). The total number of spines was also reduced in the *Hnrnph2^P209L/Y^* mice, although this difference did not reach statistical significance (Figure 5D). Notably, the spine density (number of spines per 10 μm) was comparable between WT and P209L mice (Figure 5E), suggesting that the observed reduction in total spine number can be attributed to reduced dendritic length. Taken together, these results suggest that overall brain growth is relatively preserved in hnRNPH2-mutant mice. However, dendritic arbor complexity is reduced in these mutant mice, suggesting that neural connection and activity may be affected.

### hnRNPH2 P209L and R206W mice, but not KO mice, have impaired motor function.

**Figure 6:**
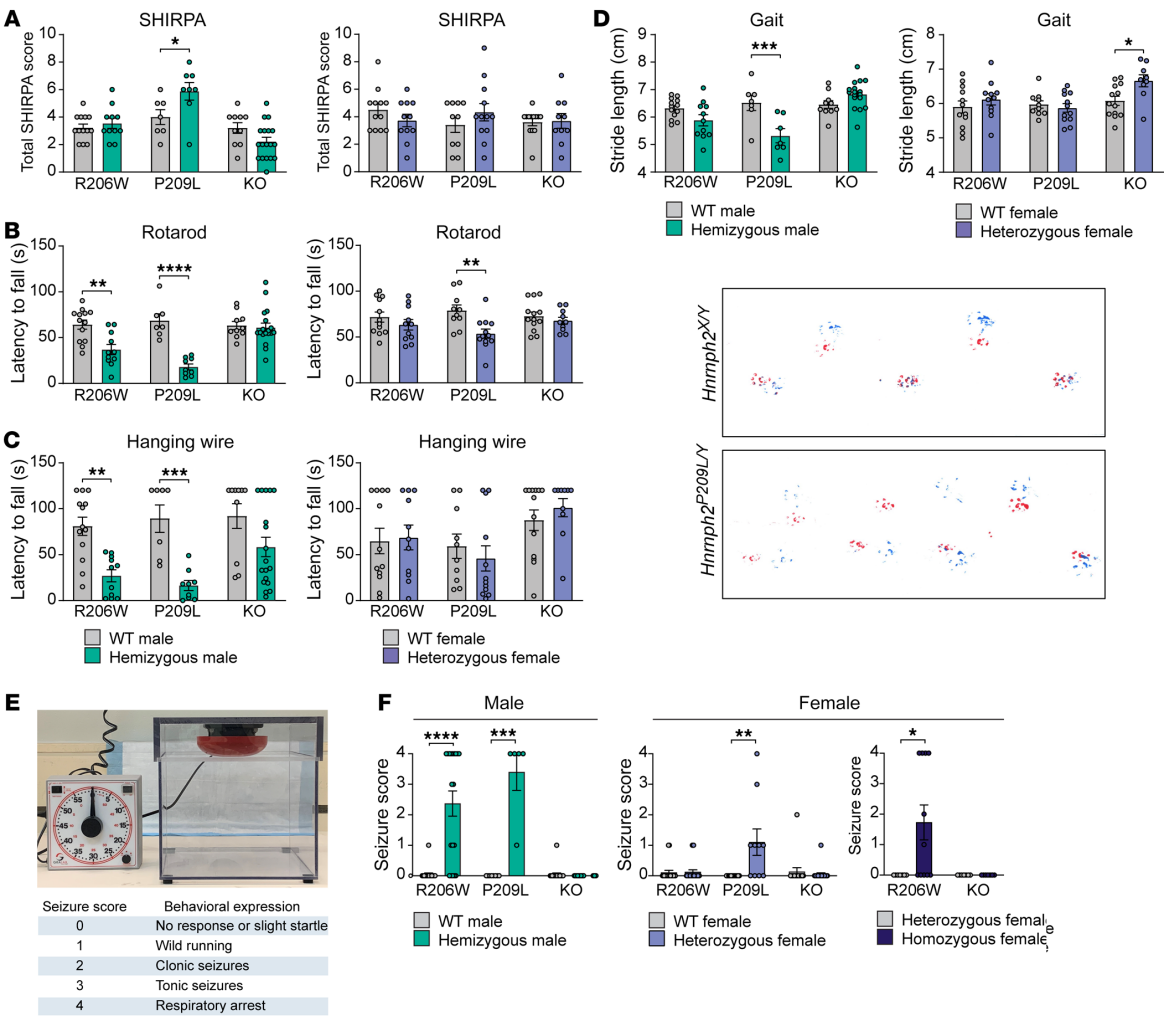
hnRNPH2-mutant mice have impaired motor function and increased susceptibility to audiogenic seizures. (**A**) Total SHIRPA abnormality scores. (**B**) Latency to fall from rotarod. (**C**) Latency to fall from a wire cage top. (**D**) Stride length and representative images showing gait (red, front paws; blue, hind paws) of an hnRNPH2 P209L male mouse and WT littermate. Group sizes for SHIRPA and motor tests: *Hnrnph2^R206W/Y^* (*n* = 11) vs. *Hnrnph2^X/Y^* (*n* = 13); *Hnrnph2^P209L/Y^* (*n* = 8) vs. *Hnrnph2^X/Y^* (*n* = 7); *Hnrnph2^KO/Y^* (*n* = 18) vs. *Hnrnph2^X/Y^* (*n* = 10); *Hnrnph2^R206W/X^* (*n* = 11) vs. *Hnrnph2^X/X^* (*n* = 12); *Hnrnph2^P209L/X^* (*n* = 12) vs. *Hnrnph2^X/X^* (*n* = 10); *Hnrnph2^KO/X^* (*n* = 10) vs. *Hnrnph2^X/X^* (*n* = 13). (**E**) Audiogenic seizure chamber and scoring of seizure behavior. (**F**) Audiogenic seizure severity score. Group sizes: *Hnrnph2^R206W/Y^* (*n* = 19) vs. *Hnrnph2^X/Y^* (*n* = 22); *Hnrnph2^P209L/Y^* (*n* = 5) vs. *Hnrnph2^X/Y^* (*n* = 8); *Hnrnph2^KO/Y^* (*n* = 20) vs. *Hnrnph2^X/Y^* (*n* = 22); *Hnrnph2^R206W/X^* (*n* = 24) vs. *Hnrnph2^X/X^* (*n* = 19); *Hnrnph2^P209L/X^* (*n* = 10) vs. *Hnrnph2^X/X^* (*n* = 12); *Hnrnph2^KO/X^* (*n* = 20) vs. *Hnrnph2^X/X^* (*n* = 15); *Hnrnph2^R206W/X^* (*n* = 7) vs. *Hnrnph2^R206W/R206W^* (*n* = 11); *Hnrnph2^KO/X^* (*n* = 7) vs. *Hnrnph2^KO/KO^* (*n* = 7). Data are shown as the mean ± SEM. *P < 0.05, **P < 0.01, ***P < 0.001, ****P < 0.0001 by 2-way nonparametric ANOVA with Mann-Whitney *U* test for group-wise comparisons (**A** and **F**) or 2-way ANOVA with Šidák’s post test (**B**–**D**). Where applicable, exact *P* values are provided in Supplemental Table 4.

In humans, pathogenic variants in *HNRNPH2* are associated with developmental delay, most often characterized by significant motor abnormalities accompanied by severe expressive and receptive language impairment (26); thus, we first focused on motor function. Mice selected for behavioral phenotyping were first subjected to an observational test battery at 8 weeks of age to obtain a broad screen of phenotypes. Using a modified version of the SHIRPA level 1 protocol, a standardized protocol for comprehensive behavioral and functional assessment (27, 28), we found that global abnormality scores were significantly higher in *Hnrnph2^P209L/Y^* male mice compared with those in their WT littermates, whereas all other mutant and KO male mice and all female mice did not differ significantly from controls (Figure 6A). When abnormality scores were generated for specific functions tested in SHIRPA, we found that motor function was significantly impaired in *Hnrnph2^R206W/Y^*, *Hnrnph2^P209L/Y^*, and *Hnrnph2^P209L/X^* mice compared with that in WT littermate controls (Supplemental Figure 10A). In addition, SHIRPA scores for autonomic function were significantly increased in hnRNPH2 P209L female mice and tended to be increased in male mice compared with WT littermates. In contrast, male KO mice had decreased autonomic function scores compared with WT controls, reflecting their better performance in assays of autonomic function (Supplemental Figure 10B). Scores for sensory and neuropsychiatric functions were not significantly different between *Hnrnph2*-mutant or -KO mice and their littermate controls (data not shown).

We next tested mice in specific and sensitive tests of motor function, including balance, coordination, and muscle strength. Rotarod performance was significantly impaired in *Hnrnph2^P209L/Y^*, *Hnrnph2^R206W/Y^*, and *Hnrnph2^P209L/X^* mice compared with WT littermates, whereas all other mutant or KO male mice and female mice did not differ significantly from controls (Figure 6B). A similar impairment of balance and coordination was observed for *Hnrnph2^P209L/Y^* and *Hnrnph2^R206W/Y^* male mice in the beam walking test (Supplemental Figure 10C). Latency to fall from a wire cage top was significantly decreased in *Hnrnph2^P209L/Y^* and *Hnrnph2^R206W/Y^* male mice (Figure 6C) and grip strength was significantly decreased in *Hnrnph2^P209L/Y^* male mice (Supplemental Figure 10D). Finally, gait analysis revealed that *Hnrnph2^P209L/Y^* male mice had significantly decreased stride length compared with that of WT littermates (Figure 6D), whereas overlap, front base width, and hind base width were unchanged (data not shown).

### hnRNPH2 P209L and R206W mice, but not KO mice, have increased susceptibility to audiogenic seizures.

Patients with hnRNPH2 mutations have reported sensory issues, including hypo- and hypersensitivity to pain, temperature, touch, and, in some cases, sound (2). Therefore, we next tested our mice for sensory function, including visual acuity, olfactory function, and pain perception. We did not find any significant impairment for any of the *Hnrnph2*-mutant or -KO mice in the optomotor response, hot plate, or scent habituation tests (Supplemental Figure 11). However, we did find a significant increase in audiogenic seizure susceptibility, which has been used as a measure of both sensory hypersensitivity and epilepsy in mouse models of monogenic autism (29, 30). At P21, *Hnrnph2^P209L/Y^*, *Hnrnph2^R206W/Y^*, and *Hnrnph2^P209L/X^* mice had significantly increased incidence and severity of audiogenic seizures (Figure 6, E and F). Similarly, *Hnrnph2^R206W/R206W^* female mice were more susceptible to audiogenic seizures compared with *Hnrnph2^R206W/X^* female littermates (Figure 6F). In contrast, *Hnrnph2*-KO mice showed no significant audiogenic seizure behavior (Figure 6F).

### hnRNPH2 P209L mice show increased delta power and epileptiform activity.

Nearly half of patients with mutations in *HNRNPH2* have been reported to have a clinical seizure, and approximately 10% have been reported to have abnormal brain activity by electroencephalography (EEG) without clinical seizures (2). Thus, we performed EEG analysis in hnRNPH2-mutant mice to measure their baseline brain and epileptic activity. To this end, we implanted electrodes near the lambdoid suture (superior/inferior colliculus regions) and between coronal and lambdoid sutures (somatosensory/visual cortex regions) to record local field potentials for 24-hour period. (Supplemental Figure 12A). We observed increased cortical power in *Hnrnph2^P209L/Y^* mice compared with WT littermate controls during the dark phase at the lead implanted at the right lambdoid suture (Supplemental Figure 12B), mainly due to increased power in the delta spectrum (Supplemental Figure 12C). However, this difference was absent during the light phase (Supplemental Figure 12, D and E) and both light and dark phases when the lead was implanted between the right coronal and lambdoid sutures (Supplemental Figure 12, F and G). Delta power is often associated with seizure susceptibility in mice (31). Quantification of epileptiform activity revealed that *Hnrnph2^P209L/Y^* mice had an increased percentage of time spent exhibiting spiking events during the dark phase (Supplemental Figure 12, H and I). Video analysis of individual mice showed no seizure-like behavior in all 4 WT mice in the experiment; however, 2 of 3 *Hnrnph2^P209L/Y^* mice did show seizure-like behavior. One *Hnrnph2^P209L/Y^* mouse exhibited absence-like seizure activity for much of the 1 hour, characterized by high electrographic activity and immobility. The second *Hnrnph2^P209L/Y^* mouse had a seizure event with abnormal EEG waveforms (Supplemental Figure 12J) that included forelimb clonus followed by a curling of the entire body with forelimb clonus. Together, these data suggest that mutation to *HNRNPH2* increases EEG delta power and enhances seizure susceptibility.

### hnRNPH2 R206W male mice have increased anxiety, impaired spatial learning and memory, deficits in social interaction, and reduced marble burying.

In addition to significant motor problems, pathogenic variants in *HNRNPH2* in humans are associated with intellectual disability and psychiatric diagnoses and concerns, including anxiety, autism spectrum disorder, social communication disorder, and obsessive-compulsive disorder or stereotyped behaviors (2). Therefore, we evaluated 8-week-old *Hnrnph2^R206W/Y^* male mice and their WT littermates for anxiety, learning and memory, social interaction, and repetitive behavior. We note that we could not perform the same assays with *Hnrnph2^P209L/Y^* mice due to their high mortality (Figure 3D).

**Figure 7:**
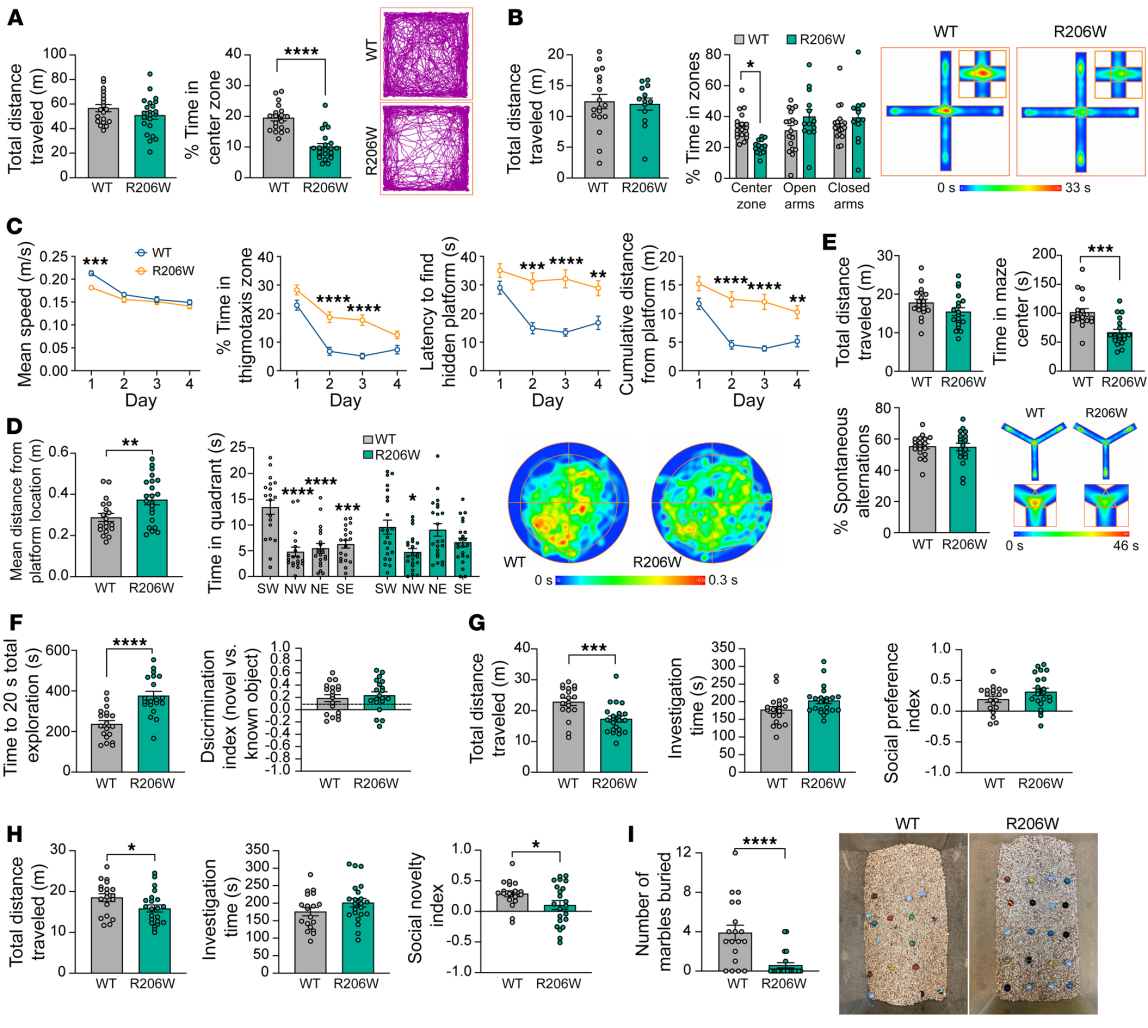
hnRNPH2 R206W male mice have increased anxiety, impaired spatial learning and memory, social interaction deficits, and reduced marble burying. (**A**) Results from open-field testing. Track plots show the position of animals’ center points from a representative mouse for the duration of the test. (**B**) Results from elevated plus maze test. Mice that fell from the maze were excluded from further analysis. Group size: *Hnrnph2^R206W/Y^* (*n* = 13), *Hnrnph2^X/Y^* (*n* = 18). Heatmaps show the average animals’ center point for the groups for the 5-minute test. Insets shows enlarged center zone of the maze. (**C** and **D**) Results from the training phase (**C**) and the probe trial (**D**) of the Morris water maze. Heatmaps show the average animals’ center point for the groups for the duration of the probe trial. (**E**) Results from Y maze spontaneous alternation testing. Group size: *Hnrnph2^R206W/Y^* (*n* = 19), *Hnrnph2^X/Y^* (*n* = 19). Heatmaps show the average animals’ center point for the groups for the 8-minute test. Insets show enlarged center zone of the maze. (**F**) Results from novel object recognition testing. Group size: *Hnrnph2^R206W/Y^* (*n* = 20), *Hnrnph2^X/Y^* (*n* = 19). Dotted line indicates previously published novel object discrimination index for C57BL/6J mice with a 24-hour test interval (ref. 50). (**G**) Results from 3-chamber social preference testing. Group size: *Hnrnph2^R206W/Y^* (*n* = 20), *Hnrnph2^X/Y^* (*n* = 19). (**H**) Results from 3-chamber social novelty testing. (**I**) Results from marble burying test. Representative cages after the test are shown. Data are shown as the mean ± SEM. Unless otherwise noted, group sizes were *Hnrnph2^R206W/Y^* (*n* = 22) and *Hnrnph2^X/Y^* (*n* = 19). *P < 0.05, **P < 0.01, ***P < 0.001, ****P < 0.0001 by unpaired *t* test (**A**; **D**, mean distance from platform location; and **E**–**I**), 2-way ANOVA with Šidák’s post test (**B** and **C**), or 2-way ANOVA with Tukey’s post test (**D**, time spent in each quadrant). Where applicable, exact *P* values are provided in Supplemental Table 4.

We began with the open-field test, which is widely used to evaluate anxiety-like behavior by measuring the amount of time spent in the open center area. In this test, mutant male mice spent significantly less time in the center zone compared with that of controls, despite similar locomotor activity, as measured by total distance traveled across the 20-minute test (Figure 7A). When data were analyzed in 5-minute time bins, the distance traveled within the first 5 minutes of the test was significantly lower for *Hnrnph2^R206W/Y^* male mice compared with controls but was similar to controls for the remainder of the test (Supplemental Figure 13A). In contrast, the percentage of time spent in the center was significantly reduced in mutants across all 4 time bins (Supplemental Figure 13A). Together, these results suggest increased anxiety with impaired habituation to a novel environment in the *Hnrnph2^R206W/Y^* male mice.

To further explore the anxiety phenotype in these mice, we used the elevated plus maze test. In this test, we noticed that significantly more R206W mutants (9 of 22) fell from the maze as compared with controls (1 of 19) (*P* = 0.0109). The frequent falls of the mutant mice off the maze were unexpected and may be related to increased anxiety levels and/or impaired motor function. For mice that completed the 5-minute test, we found no significant difference in the total distance traveled, suggesting similar locomotor activity (Figure 7B). However, the percentage of time spent in the center zone was significantly reduced in *Hnrnph2^R206W/Y^* male mice, whereas the percentage of time spent in the open or closed arms was comparable between control and mutant mice (Figure 7B). As time spent in the center of the elevated plus maze test has been linked with decision making related to approach/avoid conflict (32), this decrease suggests impaired decision making and risk assessment in the *Hnrnph2^R206W/Y^* male mice. When analyzing data in 1-minute time bins, we found that WT mice, but not *Hnrnph2^R206W/Y^* male mice, spent significantly less time in the open arms and more time in the closed arms over time (Supplemental Figure 13B). Increasing open-arm platform avoidance and closed-arm preference is expected as the test progresses, a change in behavior that is considered to reflect the avoidance of potentially dangerous sections of the maze (33). Thus, the failure of mutant mice to avoid the more dangerous open arm platforms over time could be due to impaired spatial learning or impaired decision making and risk assessment (33, 34).

To assess spatial learning and memory, we used the Morris water maze, which assesses cued learning, spatial learning, and memory, as determined by preference for the platform area when the platform is absent. During the cued phase of the test, there was no significant difference in mean speed or percentage thigmotaxis (percentage time spent within 10 cm of the pool wall) between *Hnrnph2^R206W/Y^* male mice and WT littermates (Supplemental Figure 13C). Although mutants took longer to find and climb onto the visible platform in early trials, both groups performed similarly in later trials (Supplemental Figure 13C). Together, these data suggest that *Hnrnph2^R206W/Y^* male mice possess the basic abilities, strategies, and motivation necessary to complete the spatial version of the task (35). During the spatial learning phase of the test, *Hnrnph2^R206W/Y^* male mice had significantly reduced swim speed on day 1 but not days 2–4 (Figure 7C). Percentage thigmotaxis was increased in mutants compared with controls on days 2 and 3 of training, in contrast to their behavior during the cued trials (Figure 7C). Given that thigmotaxis has been linked to anxiety, stress, and fear (36, 37), it is possible that repeated swimming trials over multiple training days increased anxiety in the *Hnrnph2^R206W/Y^* male mice. Thigmotaxis can also interfere with an animal’s ability to learn the location of the hidden platform, and it was therefore not surprising that the latency to find the platform, as well as the cumulative distance from the platform, were both significantly reduced in the mutant group on days 2–4 of training (38) (Figure 7C and Supplemental Figure 13D). In the probe trial to assess memory, *Hnrnph2^R206W/Y^* male mice did not demonstrate significant thigmotaxis but did have significantly slower mean swim speed compared with WT littermates (Supplemental Figure 13E). Furthermore, in contrast to controls, mutant mice did not show a clear preference for the target quadrant, and mean distance from the target was significantly higher (Figure 7D). Given the increased thigmotaxis and apparent reduced spatial learning in the learning phases of the test, it is not possible to say whether the impaired performance in the probe trial was due to deficits in spatial learning, spatial memory, or both.

Next, we tested the mice in the Y maze spontaneous alternation test, a measure of working spatial memory. In this maze, mice typically prefer to investigate a new arm of the maze rather than returning to one that was previously visited. Total distance traveled during the test was not significantly different between groups, suggesting comparable locomotor activity (Figure 7E). However, the number of arm entries was significantly reduced in *Hnrnph2^R206W/Y^* male mice compared with WT littermates (Supplemental Figure 13F). As was observed in the elevated plus maze test, the *Hnrnph2^R206W/Y^* male mice spent significantly less time in the center zone (Figure 7E), the region of the Y maze in which working memory is thought to be engaged (39). The percentage of spontaneous alternations was not significantly different between mutants and controls (Figure 7E). As there was no significant correlation between total number of arm entries and percent spontaneous alternations (Supplemental Figure 13F), these results suggest that *Hnrnph2^R206W/Y^* male mice have intact spatial working memory in the Y maze.

We next performed the novel object recognition test, which assesses visual recognition memory. In this test, mice are presented with a choice between novel and familiar objects, with normal behavior typically reflecting rodents’ innate preference for novelty. In the familiarization phase of the test, *Hnrnph2^R206W/Y^* male mice took longer to reach 20 seconds of total exploration of both objects, suggesting decreased exploration. For 20-second total exploration, neither mutants nor WT littermates showed significant preference for identical objects placed at the top left versus bottom right of the open field (Supplemental Figure 13G). In the testing phase of the test performed 24 hours later, *Hnrnph2^R206W/Y^* male mice again had significantly increased total time to reach 20 seconds of object exploration. For 20-second total exploration, *Hnrnph2^R206W/Y^* male mice showed no significant deficit in novel object recognition, as measured by the discrimination index (Figure 7F).

We next used the 3-chamber social test to assess social interaction and preference. In the social preference test, *Hnrnph2^R206W/Y^* male mice showed a significant reduction in total distance traveled, indicating reduced locomotor activity (Figure 7G). However, total investigation time (social plus nonsocial stimulus investigation time) and social preference index scores were not significantly different between groups (Figure 7G). Similarly, in the social novelty test, mutant male mice had a significant decrease in total distance traveled but a similar total investigation time (novel plus known social investigation time) (Figure 7H). However, the social novelty index was significantly reduced in *Hnrnph2^R206W/Y^* male mice compared with that in littermate controls (Figure 7H). Together, these data suggest that while *Hnrnph2^R206W/Y^* male mice have intact social preference, they exhibit deficits in social recognition memory.

Finally, we performed the marble burying test to assess any potential repetitive, obsessive compulsive-like behavior in *Hnrnph2^R206W/Y^* male mice. In this test, the number of marbles buried correlates with the intensity of the mouse’s repetitive or compulsive digging behavior. To our surprise, we found that mutant male mice buried significantly fewer marbles compared with WT littermates (Figure 7I), suggesting a deficit in species-typical digging behavior (40), although the reduced overall activity observed in mutant mice may also have contributed to the decreased marble burying.

### Pathogenic variants alter the nucleocytoplasmic ratio of hnRNPH2 in mice.

To examine the effect of disease-causing mutations on the subcellular localization of hnRNPH2 in mice, we performed immunoblot analysis on nuclear and cytoplasmic fractions of cortical tissue. Nuclear hnRNPH2 levels were significantly reduced in *Hnrnph2^P209L/Y^* male mice and tended to be decreased in *Hnrnph2^R206W/Y^* male mice (Supplemental Figure 14, A and B). As we were unable to detect any hnRNPH2 protein in the cytoplasmic fraction of mutants or WT littermate controls by immunoblot (data not shown), we next examined brain sections, including hippocampus and cerebellum, using immunofluorescence, a technique that is more sensitive than immunoblot. Using an antibody specific for hnRNPH2, we observed cytoplasmic staining in neurons of both R206W and P209L mutants but not in WT littermate controls (Supplemental Figure 14, C–E). Together, these results suggest that disease-causing mutations modestly alter the subcellular localization of hnRNPH2 in neurons of mice, similar to what we observed for human hnRNPH2 in HeLa cells. Importantly, despite mutations to the PY-NLS, the vast majority of mutant hnRNPH2 was correctly localized in the nucleus of mouse neurons in vivo (Supplemental Figure 14, C–E), consistent with our observations in HeLa cells (Figure 1, C–E).

### Expression of Hnrnph1 is increased in Hnrnph2-KO mice but not in hnRNPH2 P209L or R206W mice.

**Figure 8:**
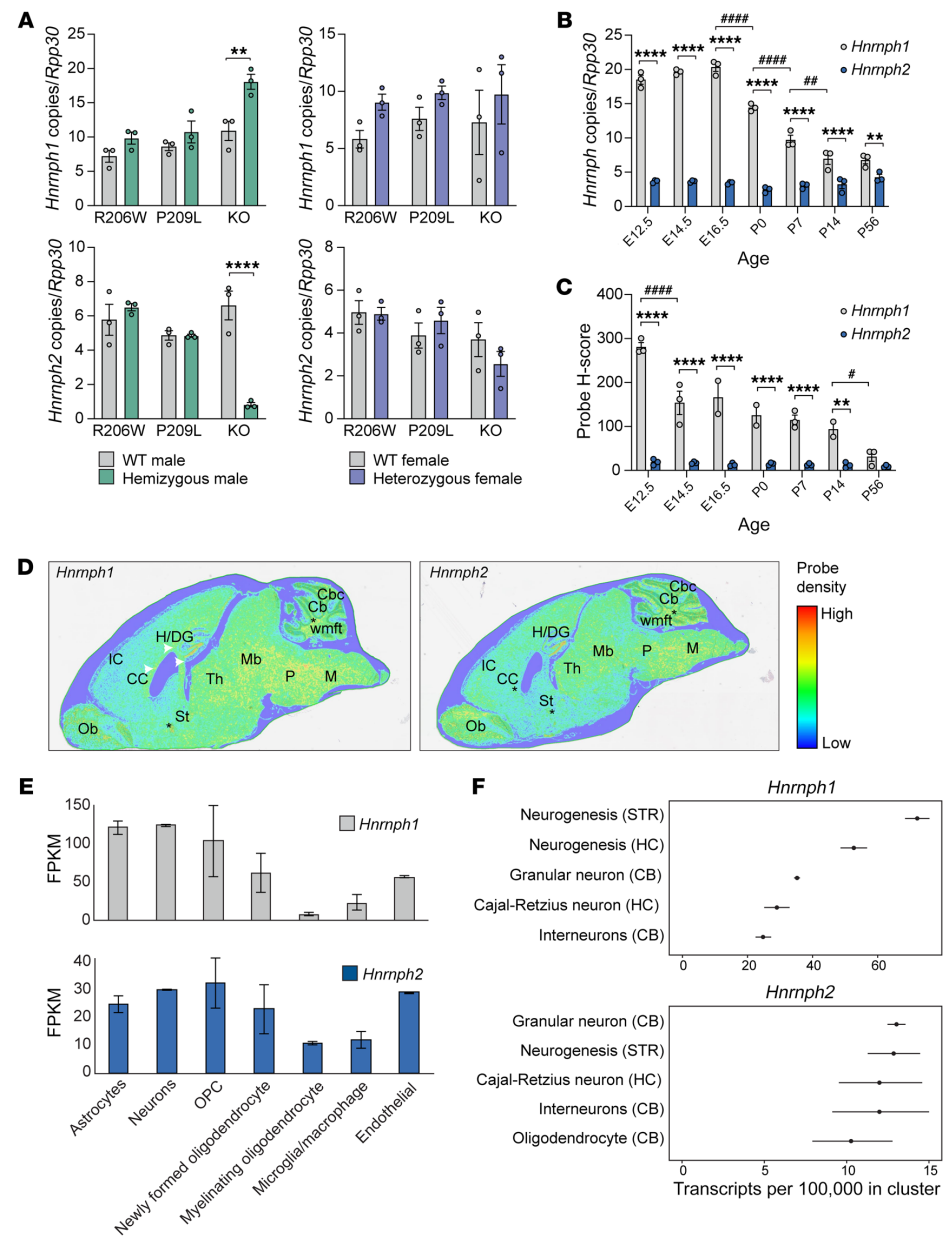
Spatiotemporal expression of *Hnrnph1* and *Hnrnph2* in mouse brain. (**A**) Number of *Hnrnph1* and *Hnrnph2* copies normalized to *Rpp30* in the cortices of indicated mice by ddRT-PCR. *n* = 3 for all groups. (**B**) Number of *Hnrnph1* and *Hnrnph2* copies normalized to *Rpp30* in the cortices of WT C67BL/6J mice across prenatal and postnatal developmental time points by ddRT-PCR. *n* = 3 for all groups. (**C**) H scores for *Hnrnph1* and *Hnrnph2* probes in the brains of WT C67BL/6J mice across developmental time points by Halo analysis of BaseScope ISH. *n* = 3 for all groups. (**D**) Regional expression of *Hnrnph1* and *Hnrnph2* across the adult (P56) mouse brain by BaseScope ISH. White arrowheads indicate corpus callosum; asterisks indicate fiber tracts. Ob, olfactory bulb; CC, corpus callosum; IC, cerebral cortex/isocortex; H/DG, hippocampus/dentate gyrus; St, bed of nuclei of the stria terminalis; P, pons; M, medulla; Th, thalamus; Mb, rostral collicular midbrain; Cb, cerebellum; Cbc, cerebellar cortex; wmft, white matter fiber tracts. (**E**) Expression of *Hnrnph1* and *Hnrnph2* in major classes of brain cells at P7 by RNA. Data extracted from Brain RNA-Seq (46, 47). FPKM, fragments per kilobase of transcript per million mapped reads. (**F**) Top 5 expression cell clusters for *Hnrnph1* and *Hnrnph2* in adult mouse brain by single-cell RNA-Seq (48). Data are shown as the mean ± SEM for **A**–**C** and mean ± SD for **E**. **P < 0.01, ****P < 0.0001 by 2-way ANOVA with Šidák’s post test; *^#^P* < 0.05, *^##^P* < 0.01, *^####^P* < 0.0001 by 2-way ANOVA with Tukey’s post test. Where applicable, exact *P* values are provided in Supplemental Table 4.

*Hnrnph1* is the autosomal conserved paralog of *Hnrnph2*, and the 2 genes are believed to play similar and potentially redundant roles in RNA splicing (41). Mutations in *HNRNPH1* are also associated with a neurodevelopmental syndrome identified in boys that is very similar to hnRNPH2-linked phenotypes (9, 10). Given the high degree of homology between the 2 genes and the possibility of redundancy in function, we examined the expression of *Hnrnph1* in our *Hnrnph2*-mutant and -KO mice using digital-droplet RT-PCR (ddRT-PCR). In the adult cortex, *Hnrnph1* mRNA levels were significantly increased in male *Hnrnph2*-KO mice but not in P209L or R206W mutant male mice (Figure 8A). The increase of *Hnrnph1* mRNA in male *Hnrnph2*-KO mice was accompanied by an increase in hnRNPH1 protein levels (Supplemental Figure 15A). In contrast, expression levels of 2 other members of the hnRNP F/H family, *Hnrnpf* and *Hnrnph3*, remained unaltered in both *Hnrnph2*-mutant and -KO mice (Supplemental Figure 15B). *Hnrnph2* mRNA levels were significantly decreased in male *Hnrnph2*-KO mice, as expected for a transcript subject to nonsense-mediated decay, but unchanged in hemizygous P209L or R206W mutant male mice. Similar trends were observed for both transcripts in female mice, but differences were not statistically significant (Figure 8A).

To test whether the increase of *Hnrnph1* mRNA in our KO mice was a consequence of loss of *Hnrnph2*, we depleted *HNRNPH2* using siRNA and measured *HNRNPH1* RNA levels in HEK293T cells. At approximately 30% KD of *HNRNPH2* (12 nM RNAi), we did not detect any increase in *HNRNPH1* transcript levels. However, when we reached approximately 60% KD of *HNRNPH2* (18 nM RNAi), we detected an increase of approximately 15% in *HNRNPH1* transcript levels. At approximately 88% and approximately 98% KD of *HNRNPH2* (24 and 30 nM RNAi), we found approximately 25% and approximately 55% increases of *HNRNPH1* transcript levels, respectively (Supplemental Figure 15C), suggesting that loss of hnRNPH2 leads to transcriptional upregulation of *HNRNPH1*.

To explore the possibility that the increase in *Hnrnph1* expression may compensate for the loss of *Hnrnph2* in KO mice, we investigated the spatiotemporal expression of these 2 genes. Assessment of the Allen mouse brain atlas revealed that *Hnrnph1* is expressed at high levels across all 12 major regions of the adult mouse brain, whereas *Hnrnph2* expression is detected at low levels in the olfactory areas and cortical subplate only (42). To examine the spatiotemporal expression of these 2 genes during mouse brain development, we performed ddRT-PCR and ISH on WT C57BL/6J mice at 7 embryonic and postnatal time points. Quantification of the ISH generated an “H score” reflecting the level of mRNA expression in the tissue section based on the detection of specific probe signal in cells of interest. In the cortex, *Hnrnph1* was expressed at significantly higher levels than *Hnrnph2* at all time points examined by ddRT-PCR (Figure 8B). Furthermore, whereas *Hnrnph1* mRNA levels decreased significantly after E16.5, *Hnrnph2* mRNA levels did not significantly change over the course of the 7 developmental time points (Figure 8B). ISH on whole brains showed similar results, with the H score for *Hnrnph1* being significantly higher than that for *Hnrnph2* at all time points except P56. Furthermore, *Hnrnph1* H scores significantly decreased after E12.5, whereas *Hnrnph2* H scores remained stable over all time points (Figure 8C). Spatial expression analysis of adult (P56) brains revealed that both *Hnrnph1* and *Hnrnph2* were expressed in similar areas, including regions within the telencephalon, brain stem, and hindbrain as well as fiber tracts (Figure 8D). A similar pattern of spatiotemporal expression has been reported for *HNRNPH1* and *HNRNPH2* in human brain (43, 44) and human brain organoids (45) (Supplemental Figure 15, D–F). In sum, *HNRNPH1* and *HNRNPH2* show similar spatial and temporal expression patterns in human and mouse brains. *HNRNPH1* is highly expressed during early developmental stages and decreases over time, whereas *HNRNPH2* expression is consistently modest throughout development, suggesting that hnRNPH1 may govern early brain developmental processes that are gradually shared with its homolog hnRNPH2 at later and/or postdevelopmental stages.

To define the types of mouse brain cells that express *Hnrnph1* and *Hnrnph2*, we turned to publicly available databases. At P7, RNA-Seq data indicated that both genes were expressed in all of the major cell classes of the brain (46, 47) (Figure 8E). In the adult mouse brain, single-cell RNA-Seq (48) has demonstrated that the top 5 expression cell clusters for *Hnrnph1* and *Hnrnph2* show significant overlap, including cells that undergo adult neurogenesis in the striatum, granular neurons in the cerebellum, and Cajal-Retzius neurons in the hippocampus (Figure 8F). Together, these data suggest that *Hnrnph1* and *Hnrnph2* have closely matching expression patterns with regard to brain regions and cell types. Importantly, as development proceeds, expression of *Hnrnph1* decreases while expression of *Hnrnph2* persists; thus, normal cellular function becomes progressively more dependent on hnRNPH2. These observations support our hypothesis that upregulation of *Hnrnph1* in the setting of *Hnrnph2*-KO mice compensates for the functional loss of hnRNPH2.

### Expression of pathogenic hnRNPH2 variants leads to more severe alterations in gene expression and RNA splicing compared with HNRNPH2 KO.

Finally, we evaluated the effects of pathogenic mutations in hnRNPH2 on the expression and splicing of its target RNAs. To test this, we performed RNA-Seq in 3-week-old neurons derived from human induced pluripotent stem cells (iPSCs) bearing hnRNPH2 R206W, R206Q, or P209L mutations or KO of *HNRNPH2*. Analysis of gene expression pattern changes revealed that pathogenic variants and KO of hnRNPH2 significantly altered global gene expression (Supplemental Figure 16, A and B). A total of 3,745 RNAs were significantly up- or downregulated in one or more conditions as compared with WT controls (Supplemental Figure 16A and Supplemental Table 2). Interestingly, the patterns of up- or downregulation caused by pathogenic mutations and KO of hnRNPH2 were very similar: genes that were up- or downregulated in KO neurons were similarly up- or downregulated in neurons expressing mutant protein (Supplemental Figure 16B). However, the levels of alteration were more severe in mutant-expressing neurons compared with KO neurons (Supplemental Figure 16B). This observation is consistent with our hypothesis that the pathomechanism of hnRNPH2-linked disorder is loss of function with an incomplete compensation by hnRNPH1. Indeed, we confirmed that *HNRNPH1* transcript levels were increased in KO neurons (152%) but were less affected in mutant-expressing neurons (99%, 108%, and 128% in R206W, P209L, and R206Q, respectively). We further noted that the severity of gene expression changes in mutant-expressing neurons was inversely correlated with the levels of *HNRNPH1* upregulation, with R206W (99% of WT expression levels) being the most severe and R206Q (128% of WT expression levels) being the least severe (Supplemental Figure 16B). The gene ontology (GO) terms linked to commonly up- or downregulated genes in mutant-expressing and KO neurons demonstrated enrichment for terms related to neuronal synapses, neuronal transport, ion channel, and innate immunity (Supplemental Figure 16, C–F). Moreover, we identified 10,691 aberrant alternative splicing events, the most common of which were skipped exons (6,829 events), followed by mutually exclusive exons (1,429 events), retained introns (930 events), alternative 3′ splice sites (843 events), and alternative 5′ splice sites (660 events) (Supplemental Figure 16, G and H).

We also performed RNA-Seq analyses using the cortices of our knockin and KO mice (Supplemental Figure 17). A much smaller number of differentially expressed genes was identified in mouse cortices compared with that observed in human iPSC-derived neurons, possibly due to the stochastic nature of gene expression coming from bulk tissues (Supplemental Figure 17, A and B and Supplemental Table 3). Due to the small number of genes altered, we were unable to perform GO analyses in R206W and KO mice; however, GO analysis of differentially expressed genes in P209L mice revealed enrichment for GO terms related to neurogenesis and differentiation, similar to those identified from the human RNA-Seq analyses, and also GO terms related to muscle (Supplemental Figure 16C). We identified 10 genes that were significantly differentially upregulated in R206W and P209L mice but not in *Hnrnph2*-KO mice (Supplemental Figure 17, D and E, and Supplemental Table 3). Interestingly, these genes all appeared as significantly differentially expressed genes in human RNA-Seq analyses (Supplemental Figure 16 and Supplemental Table 2) and are all known to have functional roles in neurons (Supplemental Figure 17, E and F). For 4 of these 10 genes, pathogenic variants have been established as the cause of neurodevelopmental disorders, including *CTNNA2* (OMIM: 618174), *TNPO2* (OMIM: 619556), *ASH1L* (OMIM: 617796), and *SHANK1* (OMIM: 209850) (Supplemental Figure 17E). Similar to RNA-Seq results from the human iPSC-derived neurons, pathogenic mutations and KO of *Hnrnph2* caused widespread changes in alternative pre-mRNA splicing (Supplemental Figure 17, G and H). Taken together, these results demonstrate that pathogenic mutations induce more severe transcriptome changes than KO both in human iPSC-derived neurons and in mice. Consistent upregulation of *HNRNPH1*/*Hnrnph1* in iPSC-derived KO neurons and KO mice, but not in mutants, suggests that hnRNPH1 might be compensating for the loss of hnRNPH2 function and thereby blunting hnRNPH2-related phenotypes in the setting of *HNRNPH2* KO.

## Discussion

The hnRNP family of proteins has a significant enrichment of de novo variants associated with neurodevelopmental disorders with similar clinical phenotypes and potentially shared molecular pathogenesis (49). Mutations in *HNRNPH2* and its close paralog *HNRNPH1* provide one such example, wherein similar mutations (i.e., missense mutations frequently located in the PY-NLS) cause syndromes with overlapping symptoms (9, 10). Here, we investigated the pathological mechanism underlying hnRNPH2-related disorder using in vitro studies, cell lines, and multiple knockin and KO mouse models.

Our in vitro characterization of the consequences of common pathogenic hnRNPH2 mutations revealed, as predicted, that mutations in the PY-NLS lead to a partial redistribution of hnRNPH2 protein from the nucleus to the cytoplasm. Notably, this redistribution was modest, with the majority of hnRNPH2 protein remaining in the nucleus. Furthermore, for several pathogenic variants that lie outside the PY-NLS of hnRNPH2, we observed no redistribution of the protein to the cytoplasm. These findings are complemented by our characterization of KO and knockin mouse models. Importantly, we found that 2 independent *Hnrnph2*-KO mouse lines were phenotypically normal across a wide variety of measures, with a consistent absence of pathological phenotypes, consistent with ongoing phenotyping of the KOMP-KO line reported by the International Mouse Phenotype Consortium. These observations strongly argue that hnRNPH2-related disease cannot be attributed to a simple loss of function. In contrast, *Hnrnph2^P209L^*- and *Hnrnph2^R206W^*-knockin mice recapitulated the modest redistribution of hnRNPH2 from the nucleus to the cytoplasm while driving a highly penetrant phenotype that reproduced multiple clinical features of human disease, including facial abnormalities, seizure propensity, reduced viability in male mice, and several behavioral abnormalities, including reductions in motor ability. Furthermore, *Hnrnph2^R206W^*-mutant mice showed increased anxiety, impaired spatial learning and memory, and deficits in social interaction, echoing several phenotypes observed in human disease and presenting additional quantifiable measures that may be modifiable with therapeutic intervention. Indeed, the extensive, robust phenotypes observed in *Hnrnph2^P209L^* and *Hnrnph2^R206W^* mice suggest strong potential for their use in preclinical studies and to reveal novel targets for therapy.

This pattern of phenotypes in physiological models — no apparent phenotypic consequence in KO mice, robust recapitulation of disease features in knockin mice — suggests two possible mechanisms as drivers of disease. The first possibility is a toxic gain of function, a mechanism with precedents in several common neurological diseases (e.g., ALS caused by mutations in *SOD1*, Parkinson’s disease caused by mutations in *SNCA*) in which disease phenotypes are absent in KO animals but are faithfully recapitulated in animals expressing disease mutations. However, our results are also consistent with an alternative disease mechanism in which mutations in *HNRNPH2* directly cause a loss of hnRNPH2 function, but the persistence of significant hnRNPH2 protein in the nucleus results in a failure to induce compensatory *HNRNPH1* expression. Indeed, *HNRNPH1* has an expression pattern that is nearly identical to that of *HNRNPH2* with respect to brain region and cell type, and it encodes a highly similar protein to hnRNPH2. Interestingly, whereas the expression of *HNRNPH1* decreases as development proceeds, the expression of *HNRNPH2* persists, such that cells become progressively more dependent upon hnRNPH2. In this context, our findings from RNA-Seq analyses — namely, that KO of *HNRNPH2/Hnrnph2* and knockin of pathogenic mutations alter gene expression into the same direction, in which knockin mutations induce more severe transcriptome changes than KO and KO, but not knockin, of *HNRNPH2/Hnrnph2* consistently leads to upregulation of *HNRNPH1/Hnrnph1* — suggest that upregulation of *Hnrnph1* is responsible for rescuing KO animals from the consequences of the loss of hnRNPH2 function. In contrast, the introduction of disease mutations in *Hnrnph2* in mice is not accompanied by significant upregulation of *Hnrnph1*. As such, disease-causing mutations in *HNRNPH2* thwart the physiological mechanism that would otherwise compensate for the loss of hnRNPH2 protein function.

Importantly, both of these possible gain-of-function and loss-of-function mechanisms would be predicted to respond positively to therapies designed to deplete expression of mutant proteins (e.g., antisense oligonucleotides). Indeed, genetic compensation between *HNRNPH1* and *HNRNPH2* suggests a therapeutic strategy wherein knockdown of *HNRNPH2* in patients would be predicted to be well tolerated — as KO of *HNRNPH2* is well tolerated in cells and in mice — while also triggering compensatory upregulation of *HNRNPH1*. Further investigation will be needed to determine the mechanisms underlying cross-regulation of *HNRNPH1* and *HNRNPH2* and how normal functions of hnRNPH2 are disrupted in disease. Of particularly high priority is determining the prospects for therapy aimed at knockdown of mutant *Hnrnph2* to look for upregulation of *Hnrnph1* and potential rescue of the phenotype in mice.

## Methods

### RNA-Seq.

Sample preparation and data processing of RNA-Seq are described in Supplemental Methods.

### Study approval.

All animal experiments were reviewed and approved by the IACUC of St. Jude Children’s Research Hospital.

### Data availability.

RNA-Seq data were deposited to GEO under accession GSE226527, which includes GSE226525 (human) and GSE226526 (mouse).

## Author contributions

AK, XY, KO, AG, BJWT, HN, and FY designed and performed laboratory experiments. AK, KO, AG, BJWT, JM, AFC, YDW, TP, HS, SSZ, YMC, JPT, and HJK analyzed data. AK, JPT, and HJK drafted and revised the manuscript. JPT and HJK supervised the overall study.

## Supplementary Material

Supplemental data

Supplemental table 1

Supplemental table 2

Supplemental table 3

Supplemental table 4

## Version 1 07/17/2023

Electronic publication
