# Supplementary material for: A murine model of hnRNPH2-related neurodevelopmental disorder reveals a mechanism for genetic compensation by Hnrnph1
Source: J Clin Invest. 2023 Jul 17;133(14):e160309. doi: 10.1172/JCI160309 (PMC10348767; doi:10.1172/JCI160309)

Supplemental Material

A murine model of hnRNPH2-related neurodevelopmental disorder reveals a mechanism for genetic compensation by *Hnrnph1*

Ane Korff[1], Xiaojing Yang[1], Kevin O'Donovan[1], Abner Gonzalez[2], Brett J. W. Teubner[3], Haruko Nakamura[1], James Messing[1], Fen Yang[1], Alexandre F. Carisey[1], Yong-Dong Wang[1], Tushar Patni[4], Heather Sheppard[5], Stanislav S. Zakharenko[3], Yuh Min Chook[2], J. Paul Taylor[1,6], and Hong Joo Kim[1]

[1] Department of Cell and Molecular Biology, St. Jude Children's Research Hospital, Memphis, Tennessee, USA
[2] Department of Pharmacology, University of Texas Southwestern Medical Center, Dallas, Texas, USA
[3] Department of Developmental Neurobiology, St. Jude Children's Research Hospital, Memphis, Tennessee, USA
[4] Department of Biostatistics, St. Jude Children's Research Hospital, Memphis, Tennessee, USA
[5] Department of Pathology, St. Jude Children's Research Hospital, Memphis, Tennessee, USA
[6] Howard Hughes Medical Institute, Chevy Chase, Maryland, USA

Address correspondence to: J. Paul Taylor, 262 Danny Thomas Place, Memphis, TN, USA 38105. Phone: 901.595.6047; Email: jpaul.taylor@stjude.org. Or to: Hong Joo Kim, 262 Danny Thomas Place, Memphis, TN, USA 38105. Phone: 901.595.2211; Email: hongjoo.kim@stjude.org.

Conflict of interest: JPT is a consultant for Nido Biosciences and Faze Medicines.

Supplemental Figures

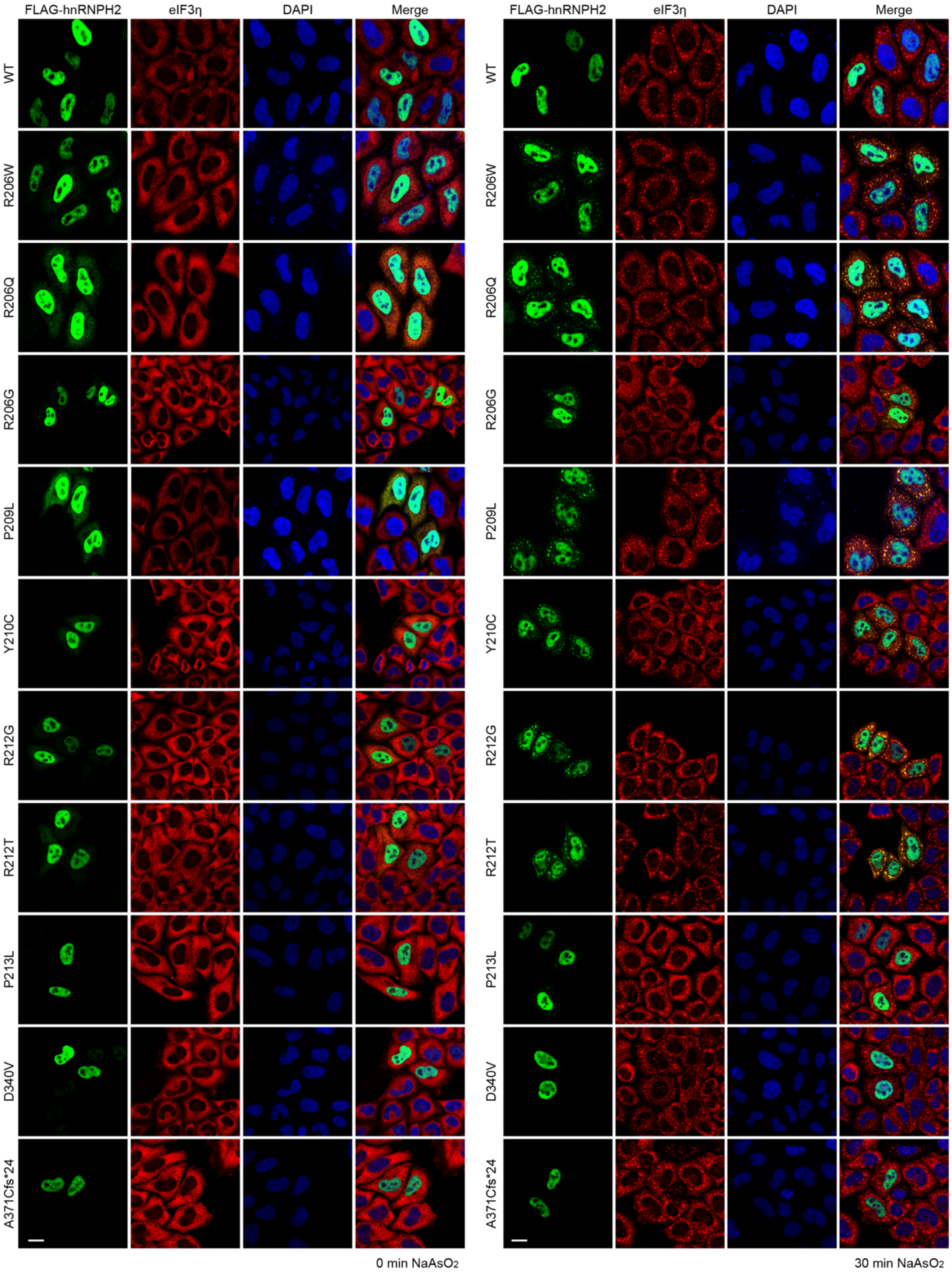

Supplemental Figure 1. Pathogenic variants alter the nucleocytoplasmic ratio of hnRNPH2 and enhance its recruitment to RNA granules. Intracellular localization of indicated FLAG-tagged hnRNPH2 proteins under basal (left) and stressed (right) conditions in HeLa cells. eIF3η was used as a cytoplasmic and stress granule marker. Scale bar, 10 µm.

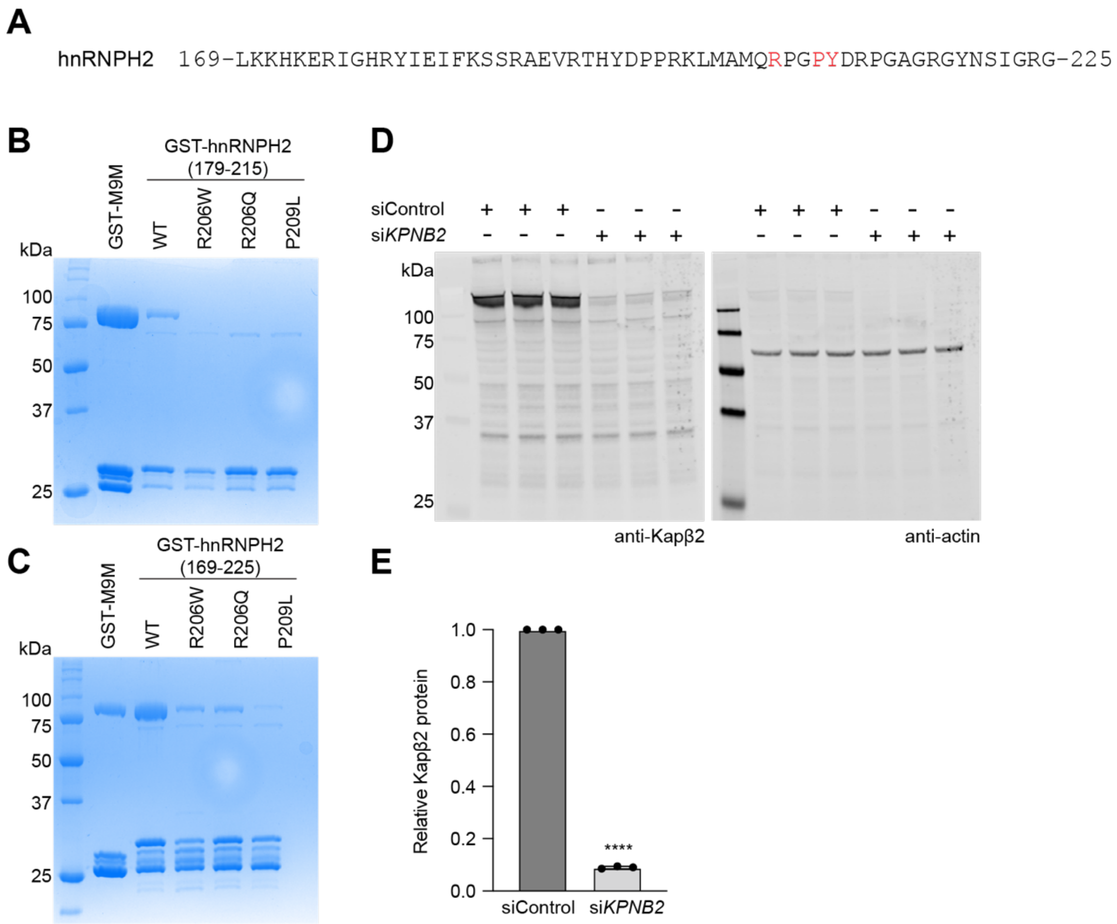

Supplemental Figure 2. Frameshift variants impair the interaction between hnRNPH2 and its nuclear transport receptor Kapβ2. (**A**) hnRNPH2 amino acid sequence used for GST pulldown experiments. Amino acid residues mutated in patients are in red. (**B** and **C**) Peptides spanning amino acids 179-215 (**B**) or 169-225 (**C**) were fused to the C terminus of GST. Gels show Coomassie Blue staining following GST pulldown of purified GST-hnRNPH2 peptides with Kapβ2. (**D**) Immunoblot showing knockdown of Kapβ2 by si*KPNB2*. Three independently prepared samples were loaded on the gel. (**E**) Quantification of Kapβ2 signal shown in (**D**). Graph shows relative Kapβ2 levels normalized to actin; error bars represent mean ± SD. ****$P <$ 0.0001 by student's t-test.

```
sp|P55795|HNRH2_HUMAN    MMLSTEGREGFVVKVRGLPWSCSADEVMRFFSDCKIQNGTSGIRFIYTREGRPSGEAFVE 60
sp|P70333|HNRH2_MOUSE    MMLSTEGREGFVVKVRGLPWSCSAEEVMRFFSDCKIQNGTSGVRFIYTREGRPSGEAFVE 60
                         ************************:*****************:*****************

sp|P55795|HNRH2_HUMAN    LESEEEVKLALKKDRETMGHRYVEVFKSNSVEMDWVLKHTGPNSPDTANDGFVRLRGLPF 120
sp|P70333|HNRH2_MOUSE    LESEDEVKLALKKDRETMGHRYVEVFKSNSVEMDWVLKHTGPNSPDTANDGFVRLRGLPF 120
                         ****:*******************************************************

sp|P55795|HNRH2_HUMAN    GCSKEEIVQFFSGLEIVPNGMTLPVDFQGRSTGEAFVQFASQEIAEKALKKHKERIGHRY 180
sp|P70333|HNRH2_MOUSE    GCSKEEIVQFFSGLEIVPNGMTLPVDFQGRSTGEAFVQFASQEIAEKALKKHKERIGHRY 180
                         ************************************************************

sp|P55795|HNRH2_HUMAN    IEIFKSSRAEVRTHYDPPRKLMAMQRPGPYDRPGAGRGYNSIGRGAGFERMRRGAYGGGY 240
sp|P70333|HNRH2_MOUSE    IEIFKSSRAEVRTHYDPPRKLMTMQRPGPYDRPGAGRGYNSIGRGAGFERMRRGAYGGGY 240
                         **********************:*************************************

sp|P55795|HNRH2_HUMAN    GGYDDYGGYNDGYGFGSDRFGRDLNYCFSGMSDHRYGDGGSSFQSTTGHCVHMRGLPYRA 300
sp|P70333|HNRH2_MOUSE    GGYDDYGGYNDGYGFGSDRFGRDLNYCFSGMSDHRYGDGGSSFQSTTGHCVHMRGLPYRA 300
                         ************************************************************

sp|P55795|HNRH2_HUMAN    TENDIYNFFSPLNPMRVHIEIGPDGRVTGEADVEFATHEDAVAAMAKDKANMQHRYVELF 360
sp|P70333|HNRH2_MOUSE    TENDIYNFFSPLNPMRVHIEIGPDGRVTGEADVEFATHEDAVAAMAKDKANMQHRYVELF 360
                         ************************************************************

sp|P55795|HNRH2_HUMAN    LNSTAGTSGGAYDHSYVELFLNSTAGASGGAYGSQMMGGMGLSNQSSYGGPASQQLSGGY 420
sp|P70333|HNRH2_MOUSE    LNSTAGTSGGAYDHSYVELFLNSTAGASGGAYGSQMMGGMGLSNQSSYGGPASQQLSGGY 420
                         ************************************************************

sp|P55795|HNRH2_HUMAN    GGGYGGQSSMSGYDQVLQENSSDYQSNLA 449
sp|P70333|HNRH2_MOUSE    GGGYGGQSSMSGYDQVLQENSSDYQSNLA 449
                         *****************************
```

Supplemental Figure 3. Sequence alignment of human and mouse hnRNPH2 proteins. Human hnRNPH2 and mouse hnRNPH2 are highly conserved. Both proteins are composed of 449 amino acids, of which only 4 amino acids differ. The PY-NLS motif (yellow highlight) within the PY-NLS (gray highlight) is absolutely conserved between the two species.

A

Location (Mouse GRCm38)	sgRNA	Sequence	Mismatches	ID	Forward HRM primer (5' to 3')	Reverse HRM primer (5' to 3')	R206W KI CFD01	R206W KI CFD24	R206W KI CFD27	P209L KI TCF01	P209L KI TCF03	P209L KI TCF06	KO TCF02	KO TCF42
X:134605507-134605529	on-target	GCTCATGACTATGCAGCGCC CGG	0	original sgRNA	-	-								
8:23407852-23407874	off-target	GGTCTTGACTATGCAGTGCC TGG	3	Hnrnph2 OTsite1	CTCCCCATCCTTTGGCTCTT	CTCCTTATGCAGCAGGTAGC	WT	WT	WT	WT	WT	Variant HRM	WT	WT
4:127641881-127641903	off-target	GCCCAGGACTATGCAGGGCC AGG	3	Hnrnph2 OTsite2	TCCAAGCTCTGGTTTCTGCA	GTTCGCTGTCGATGGTTGAC	WT	WT	WT	WT	WT	Variant HRM	WT	WT
6:133972024-133972046	off-target	GTTCATGGCTATGCAGCCCC AGG	3	Hnrnph2 OTsite3	GCCTGAAGCAATGGTGAAGG	AGCTCCTGGACTTCCATGTG	WT	WT	WT	WT	WT	Variant HRM	WT	WT
3:108380888-108380910	off-target	GCGCATGACTATCCAGCTCC AGG	3	Hnrnph2 OTsite4	TACCCGTCTCAACAAGAGCC	TGTATGTGTAACTGCCTGCCT	WT	WT	WT	WT	WT	Variant HRM	WT	WT
8:15022854-15022876	off-target	GCTCTTGACTATGGAGCACC AGG	3	Hnrnph2 OTsite5	TACTGTCCTGCGTCTTCTGG	AAGACCACAAGCTCCCTGAG	WT	WT	WT	WT	WT	Variant HRM	WT	WT
5:76327518-76327540	off-target	GCTCATGACTGTGCCGCCCC TGG	3	Hnrnph2 OTsite6	ATAGCTGGCTTCCATCCACC	ATGGCAGCTTTCCCATGGA	WT	WT	WT	WT	WT	Variant HRM	WT	WT
2:106895077-106895099	off-target	GCTGATGACTATGAAGCGTC TGG	3	Hnrnph2 OTsite7	TCCAAGAAGAGGAGAAGAGGG	CCAGATTCTCGCAGCTCCTA	WT	WT	WT	WT	WT	Variant HRM	WT	WT
X:65767936-65767958	off-target	GCTTATGACTATGCAGCAGC AGG	3	Hnrnph2 OTsite8	CCACATAGTCCCAAGTGGCT	AGAGACTTGAGGGTAGAAAGGA	WT	WT	WT	WT	WT	Variant HRM	WT	WT
1:193242538-193242560	off-target	GCTCATGACTGTGCAGGGCT GGG	3	Hnrnph2 OTsite9	TGCGTGGTGGATTTCATAATGG	CCTCCCTGACAAGCCATTCT	WT	WT	WT	WT	WT	Variant HRM	WT	WT

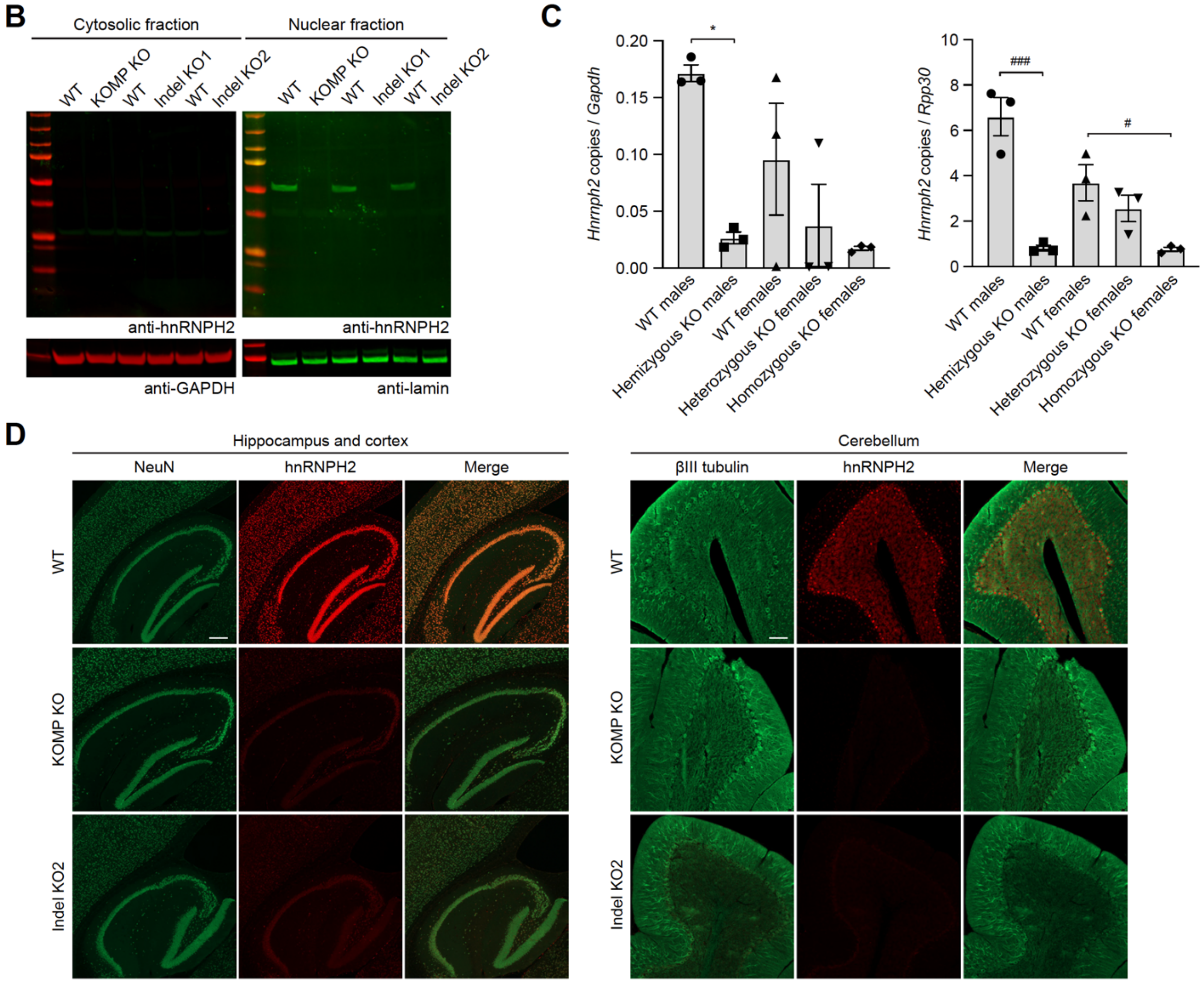

Supplemental Figure 4. Generation and validation of *Hnrnph2* mutant and KO mouse lines. (**A**) Off-target analysis of knock-in mice generated. Lines CFD01, TCF03, and TCF02 were selected for *Hnrnph2*^{R206W}, *Hnrnph2*^{P209L}, and *Hnrnph2*^{KO} genotypes, respectively, for further experiments. (**B**) Western blot of hnRNPH2 expression in cortex of WT, commercially available *Hnrnph2* KO line (KOMP KO, C57BL/6NJ-*Hnrnph2*$^{em1(IMPC)J}$/Mmjax), and two KO lines we generated (Indel KO1 (TCF42) and Indel KO2 (TCF02)). Indel KO2 (line TCF02) was chosen as the *Hnrnph2* KO line for further experiments. Representative images from n ≥ 3 experiments. (**C**) Expression of *Hnrnph2* transcript by ddRT-PCR in the cortex of *Hnrnph2* KO (Indel KO2 (TCF02)) mice. Normalization to *Gapdh* and *Rpp30* are shown; error bars represent mean ± SEM. *P = 0.0121 WT males vs. hemizygous *Hnrnph2* KO males with normalization to *Gapdh*, $^{###}P$ = 0.0001 WT males vs. hemizygous *Hnrnph2* KO males with normalization to *Rpp30*, $^{#}P$ = 0.0153 WT females vs. homozygous *Hnrnph2* KO females with normalization to *Rpp30,* by one-way ANOVA with Sidak's multiple comparisons test. n = 3 for all groups. (**D**) Immunofluorescent

staining of hnRNPH2 in brain sections of WT, a commercially available *Hnrnph2* KO line (KOMP KO), and a newly generated *Hnrnph2* KO line (Indel KO2). Representative images are shown from $n \geq 3$ experiments. NeuN and βIII tubulin were used as neuronal nuclear and cytoplasmic markers, respectively. Scale bar, 200 μm.

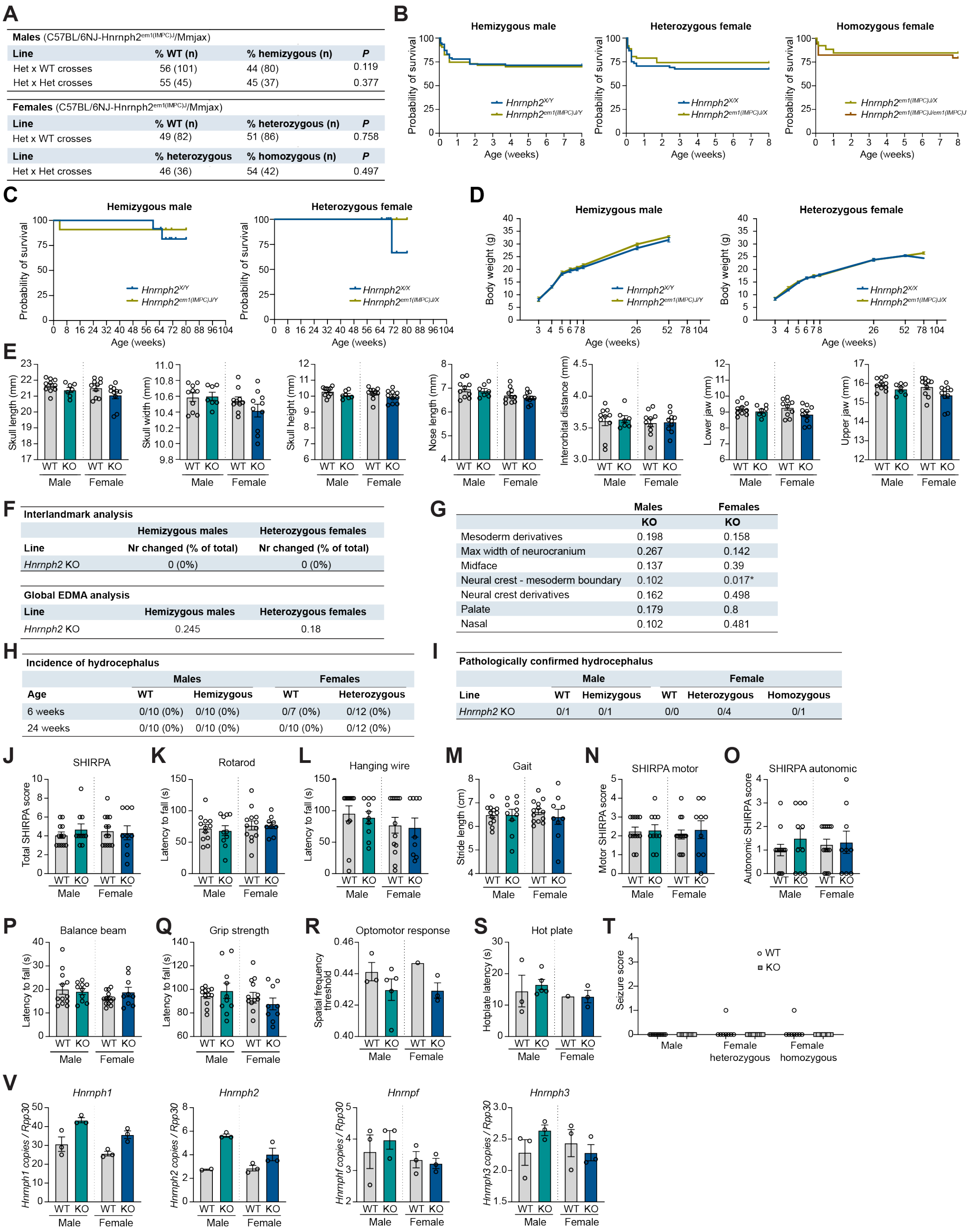

Supplemental Figure 5. Summary of data from *Hnrnph2*$^{em1(IMPC)J}$/Mmjax (KOMP) KO mice. (**A**) Ratios of genotyped mice organized by sex and breeding strategy. (**B**) Kaplan-Meier survival curves of male and female mice up to 8 weeks of age. *P* = 0.8028 (HR = 1.079), 0.4151

(HR = 0.7729), and 0.5998 (HR = 1.378) for hemizygous male, heterozygous female, and homozygous female, respectively. Group sizes were as follows: *Hnrnph2*$^{em1(IMPC)J/Y}$ (n = 63) vs. *Hnrnph2*$^{X/Y}$ (n = 77); *Hnrnph2*$^{em1(IMPC)J/X}$ (n = 62) vs. *Hnrnph2*$^{X/X}$ (n = 68); *Hnrnph2*$^{em1(IMPC)J/em1(IMPC)J}$ (n = 34) vs. *Hnrnph2*$^{em1(IMPC)J/X}$ (n = 26). (**C**) Kaplan-Meier survival curves of male and female mice up to 2 years of age. *P* = 0.6406 (HR = 0.5688) and 0.1573 (HR = undefined) for hemizygous male and heterozygous female, respectively. Group sizes were as follows: *Hnrnph2*$^{em1(IMPC)J/Y}$ (n = 11) vs. *Hnrnph2*$^{X/Y}$ (n = 12); *Hnrnph2*$^{em1(IMPC)J/X}$ (n = 9) vs. *Hnrnph2*$^{X/X}$ (n = 13). (**D**) Mean body weight of male and female mice over time. Error bars represent mean ± SEM. Group sizes were as follows: *Hnrnph2*$^{em1(IMPC)J/Y}$ (n = 11) vs. *Hnrnph2*$^{X/Y}$ (n = 12); *Hnrnph2*$^{em1(IMPC)J/X}$ (n = 9) vs. *Hnrnph2*$^{X/X}$ (n = 13). (**E**) Linear measurements of key craniofacial parameters in hemizygous male and heterozygous female mice. Error bars represent mean ± SEM. Group sizes for craniofacial analysis by µCT were as follows: *Hnrnph2*$^{em1(IMPC)J/Y}$ (n = 7) vs. *Hnrnph2*$^{X/Y}$ (n = 10); *Hnrnph2*$^{em1(IMPC)J/X}$ (n = 10) vs. *Hnrnph2*$^{X/X}$ (n = 10). (**F**) Number of significantly changed linear inter-landmark distances (top) and results of global EDMA analysis (bottom). (**G**) *P* values for regional EDMA analysis for hemizygous males and heterozygous females. (**H**) Incidence of hydrocephalus at 6 and 24 weeks of age. (**I**) Number of mice found dead or flagged for domed heads with pathologically confirmed hydrocephalus. (**J-M**) Characterization of hemizygous male and heterozygous female mice showing (**J**) total SHIRPA abnormality scores, (**K**) latency to fall from rotarod, (**L**) latency to fall from a wire cage top, and (**M**) quantification of stride length. (**N-O**) Subdomain SHIRPA scores for hemizygous male and heterozygous female mice are shown for (**N**) motor function and (**O**) autonomic function. (**P-S**) Characterization of hemizygous male and heterozygous female mice showing (**P**) latency to cross a balance beam, (**Q**) grip strength, (**R**) optomotor response test of visual acuity, and (**S**) hot plate test of pain response. Group sizes for SHIRPA and motor tests were as follows: *Hnrnph2*$^{em1(IMPC)J/Y}$ (n = 10) vs. *Hnrnph2*$^{X/Y}$ (n = 12); *Hnrnph2*$^{em1(IMPC)J/X}$ (n = 9) vs. *Hnrnph2*$^{X/X}$ (n = 13). Group sizes for sensory tests were as follows: *Hnrnph2*$^{em1(IMPC)J/Y}$ (n = 5) vs. *Hnrnph2*$^{X/Y}$ (n = 3); *Hnrnph2*$^{em1(IMPC)J/X}$ (n = 3) vs. *Hnrnph2*$^{X/X}$ (n = 1). (**T**) Audiogenic seizure severity scores. Group sizes were as follows: *Hnrnph2*$^{em1(IMPC)J/Y}$ (n = 11) vs. *Hnrnph2*$^{X/Y}$ (n = 11); *Hnrnph2*$^{em1(IMPC)J/X}$ (n = 11) vs. *Hnrnph2*$^{X/X}$ (n = 8); *Hnrnph2*$^{em1(IMPC)J/X}$ (n = 8) vs. *Hnrnph2*$^{em1(IMPC)J/em1(IMPC)J}$ (n = 7). (**U**) Number of copies of *Hnrnph1*, *Hnrnph2*, *Hnrnpf*, and *Hnrnph3* normalized to *Rpp30* in the cortex of *Hnrnph2*$^{em1(IMPC)J}$/Mmjax hemizygous male and heterozygous female KO mice by ddRT-PCR. n = 3 per group. In all analyses, WT mice are littermate controls. Error bars represent mean ± SEM in graphs J-U.

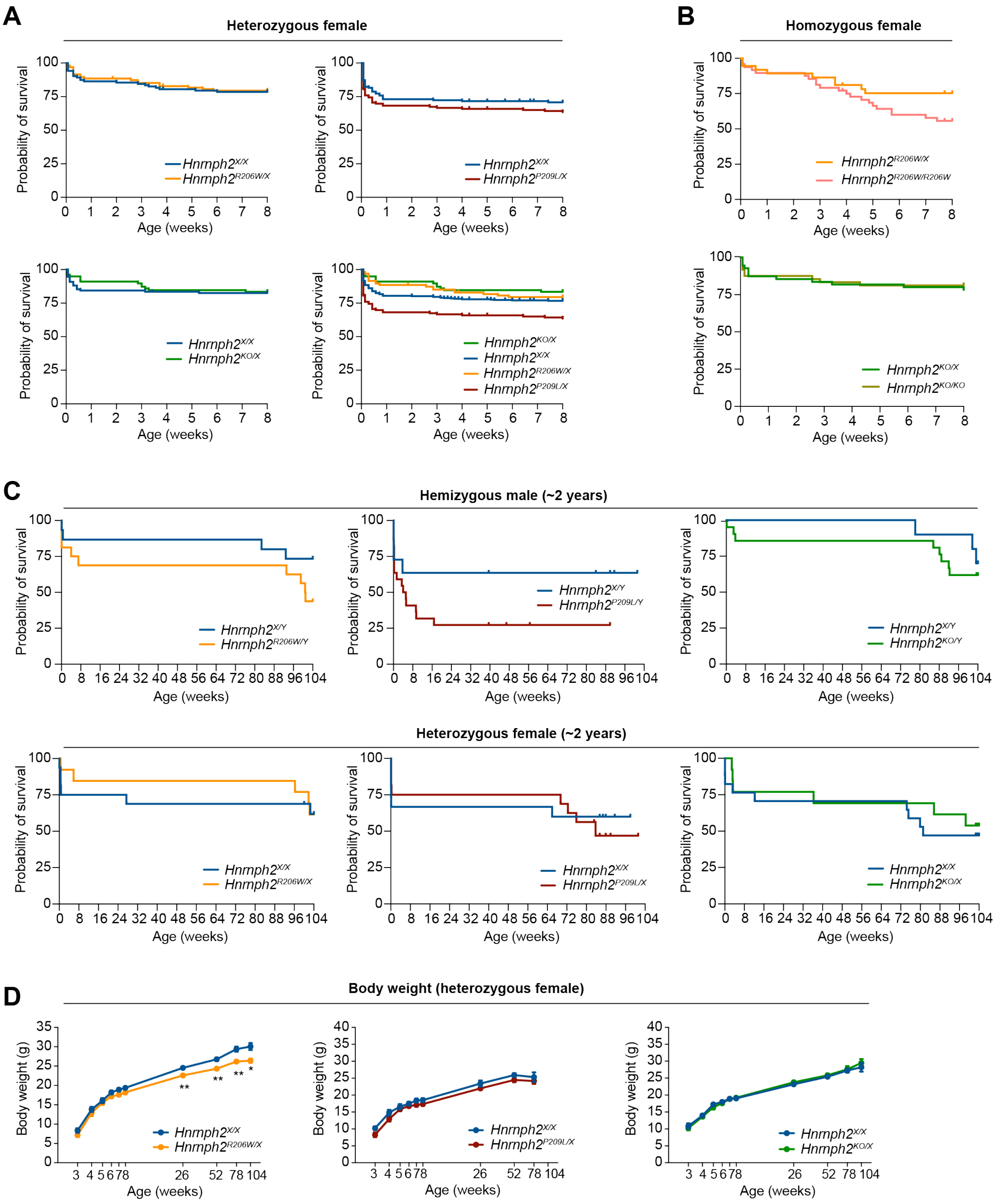

Supplemental Figure 6. Survival and body weight of *Hnrnph2* mice. (**A-B**) Kaplan-Meier survival curves of female heterozygous (**A**) and homozygous (**B**) mice up to 8 weeks of age. (**A**) P = 0.8296, HR = 0.9355 for *Hnrnph2*$^{R206W/X}$ (n = 95) vs. *Hnrnph2*$^{X/X}$ (n = 103); P = 0.1893, HR = 1.306 for *Hnrnph2*$^{P209L/X}$ (n = 129) vs. *Hnrnph2*$^{X/X}$ (n = 141); P = 0.8142, HR = 0.9197 for *Hnrnph2*$^{KO/X}$ (n = 78) vs. *Hnrnph2*$^{X/X}$ (n = 109) by Mantel-Cox test. Bottom right graph shows

overlay of all individual graphs. (**B**) *P* = 0.1026, HR = 1.889 for *Hnrnph2*$^{R206W/R206W}$ (n = 48) vs. *Hnrnph2*$^{R206W/X}$ (n = 37); *P* = 0.7415, HR = 0.8664 for *Hnrnph2*$^{KO/KO}$ (n = 48) vs. *Hnrnph2*$^{KO/X}$ (n = 55) by Mantel-Cox test. (**C**) Kaplan-Meier survival curves of male and female mice up to 2 years of age. For males, *P* = 0.1288, HR = 2.386 for *Hnrnph2*$^{R206W/Y}$ (n = 16) vs. *Hnrnph2*$^{X/Y}$ (n = 15); *P* = 0.1154, HR = 2.272 for *Hnrnph2*$^{P209L/Y}$ (n = 22) vs. *Hnrnph2*$^{X/Y}$ (n = 11); *P* = 0.5484, HR = 1.496 for *Hnrnph2*$^{KO/Y}$(n = 21) vs. *Hnrnph2*$^{X/Y}$ (n = 10) by Mantel-Cox test. For females, *P* = 0.8944, HR = 0.9236 for *Hnrnph2*$^{R206W/X}$ (n = 13) vs. *Hnrnph2*$^{X/X}$ (n = 16); *P* = 0.6974, HR = 1.219 for *Hnrnph2*$^{P209L/X}$ (n = 16) vs. *Hnrnph2*$^{X/X}$ (n = 15); *P* = 0.6151, HR = 0.7691 for *Hnrnph2*$^{KO/X}$ (n = 13) vs. *Hnrnph2*$^{X/X}$ (n = 17) by Mantel-Cox test. (**D**) Mean body weight of female mice over time; error bars represent mean ± SEM. *Hnrnph2*$^{R206W/X}$ vs. *Hnrnph2*$^{X/X}$ ***P* < 0.01 at 26, 52, and 78 weeks, **P* = 0.045 at 104 weeks by mixed-effects model (REML) with Sidak's multiple comparisons test. Group sizes were as follows: *Hnrnph2*$^{R206W/X}$ (n = 11) vs. *Hnrnph2*$^{X/X}$ (n = 12); *Hnrnph2*$^{P209L/X}$ (n = 12) vs. *Hnrnph2*$^{X/X}$ (n = 10); *Hnrnph2*$^{KO/X}$ (n = 10) vs. *Hnrnph2*$^{X/X}$ (n = 13).

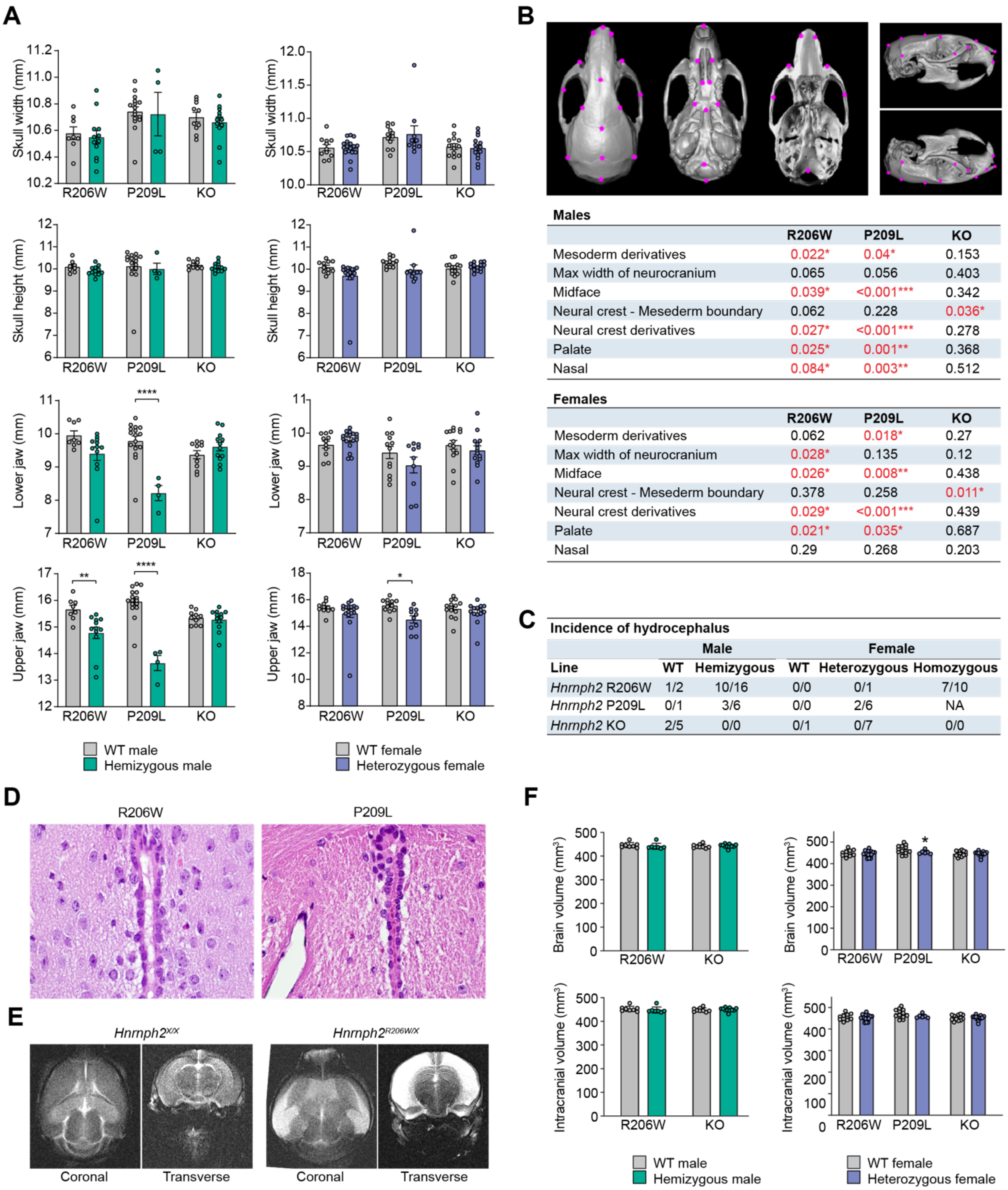

Males

	R206W	P209L	KO
Mesoderm derivatives	0.022*	0.04*	0.153
Max width of neurocranium	0.065	0.056	0.403
Midface	0.039*	<0.001***	0.342
Neural crest - Mesederm boundary	0.062	0.228	0.036*
Neural crest derivatives	0.027*	<0.001***	0.278
Palate	0.025*	0.001**	0.368
Nasal	0.084*	0.003**	0.512

Females

	R206W	P209L	KO
Mesoderm derivatives	0.062	0.018*	0.27
Max width of neurocranium	0.028*	0.135	0.12
Midface	0.026*	0.008**	0.438
Neural crest - Mesederm boundary	0.378	0.258	0.011*
Neural crest derivatives	0.029*	<0.001***	0.439
Palate	0.021*	0.035*	0.687
Nasal	0.29	0.268	0.203

Incidence of hydrocephalus

	Male		Female		
Line	WT	Hemizygous	WT	Heterozygous	Homozygous
Hnrnph2 R206W	1/2	10/16	0/0	0/1	7/10
Hnrnph2 P209L	0/1	3/6	0/0	2/6	NA
Hnrnph2 KO	2/5	0/0	0/1	0/7	0/0

Supplemental Figure 7. Craniofacial dysmorphology and hydrocephalus in *Hnrnph2* mice. (**A**) Key craniofacial parameters measured manually on individual µCT scans. Error bars represent mean ± SEM. Lower jaw length: *****P* < 0.0001 *Hnrnph2*$^{P209L/Y}$ vs. *Hnrnph2*$^{X/Y}$; upper jaw: ***P* = 0.0014 *Hnrnph2*$^{R206W/Y}$ vs. *Hnrnph2*$^{X/Y}$; *****P* < 0.0001 *Hnrnph2*$^{P209L/Y}$ vs. *Hnrnph2*$^{X/Y}$; **P* = 0.0139 *Hnrnph2*$^{P209L/X}$ vs. *Hnrnph2*$^{X/X}$ by two-way ANOVA with Sidak's multiple comparisons test. Group sizes for µCT were as follows: *Hnrnph2*$^{R206W/Y}$ (n = 12) vs. *Hnrnph2*$^{X/Y}$

(n = 8); *Hnrnph2*$^{P209L/Y}$ (n = 4) vs. *Hnrnph2*$^{X/Y}$ (n = 16); *Hnrnph2*$^{KO/Y}$ (n = 12) vs. *Hnrnph2*$^{X/Y}$ (n = 10); *Hnrnph2*$^{R206W/X}$ (n = 17) vs. *Hnrnph2*$^{X/X}$ (n = 11); *Hnrnph2*$^{P209L/X}$ (n = 10) vs. *Hnrnph2*$^{X/X}$ (n = 12); *Hnrnph2*$^{KO/X}$ (n = 14) vs. *Hnrnph2*$^{X/X}$ (n = 14). (**B**) Figure depicting the subset of anatomically relevant landmarks used in the regional EDMA analysis and *P* values of the regional EDMA analysis. (**C**) Number of mice found dead or flagged for domed heads with pathologically confirmed hydrocephalus. (**D**) H&E staining showing patent aqueducts in hnRNPH2 P209L (hemizygous male) and hnRNPH2 R206W (heterozygous female) mice with hydrocephalus. (**E**) Representative MRI images showing hydrocephalus in a *Hnrnph2*$^{R206W/X}$ heterozygous female compared with a WT littermate. (**F**) Brain volume (grey matter + white matter) and intracranial volume (brain volume + CSF volume) measured on individual MRI scans. Error bars represent mean ± SEM. *P = 0.0496 *Hnrnph2*$^{P209L/X}$ vs. *Hnrnph2*$^{X/X}$ by two-way ANOVA with Sidak's multiple comparisons test. Group sizes for brain volume analysis were as follows: *Hnrnph2*$^{R206W/Y}$ (n = 9) vs. *Hnrnph2*$^{X/Y}$ (n = 7); *Hnrnph2*$^{KO/Y}$ (n = 11) vs. *Hnrnph2*$^{X/Y}$ (n = 9); *Hnrnph2*$^{R206W/X}$ (n = 16) vs. *Hnrnph2*$^{X/X}$ (n = 11); *Hnrnph2*$^{P209L/X}$ (n = 7) vs. *Hnrnph2*$^{X/X}$ (n = 12); *Hnrnph2*$^{KO/X}$ (n = 12) vs. *Hnrnph2*$^{X/X}$ (n = 14).

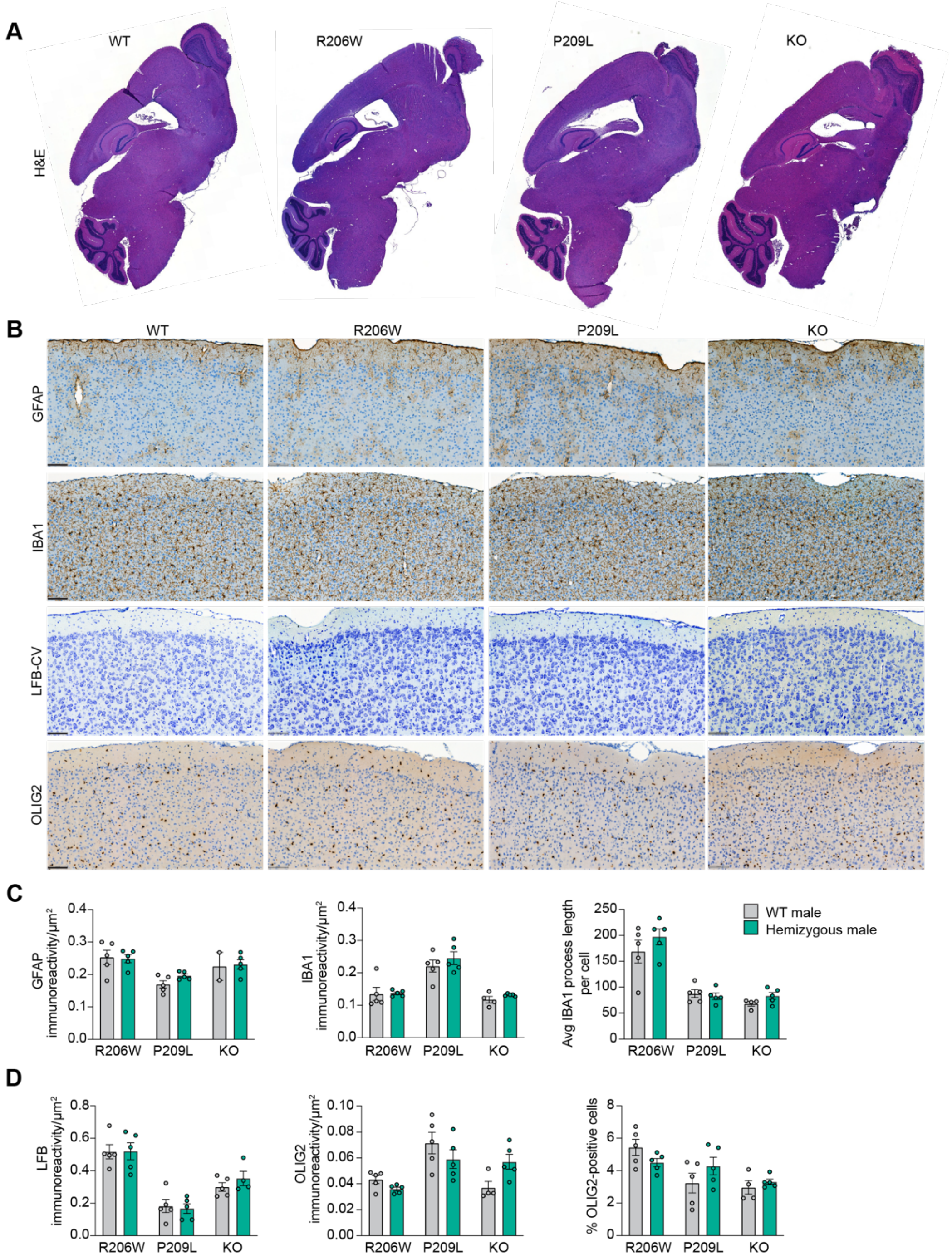

Supplemental Figure 8. H&E staining, Luxol fast blue-cresyl violet (LFB-CV) staining, and immunohistochemistry in hnRNPH2 P209L hemizygous male mouse brains. (**A**) H&E staining showing no gross abnormalities in the brains of mutant males compared to WT littermates. (**B**) Immunohistochemistry with markers against astrocytes (GFAP), microglia (IBA1), and oligodendrocytes (OLIG2) was normal. LFB-CV staining also showed regular morphology of neurons in gray and white matters. Representative images of the primary motor and somatosensory cortex for each stain and marker are shown. Scale bar, 100 μm. (**C** and **D**) Quantification of GFAP and IBA1 (**C**) and LFB-CV staining and OLIG2 immunoreactivity (**D**) in the whole brain are shown. Error bars represent mean ± SEM. n = 5 per group.

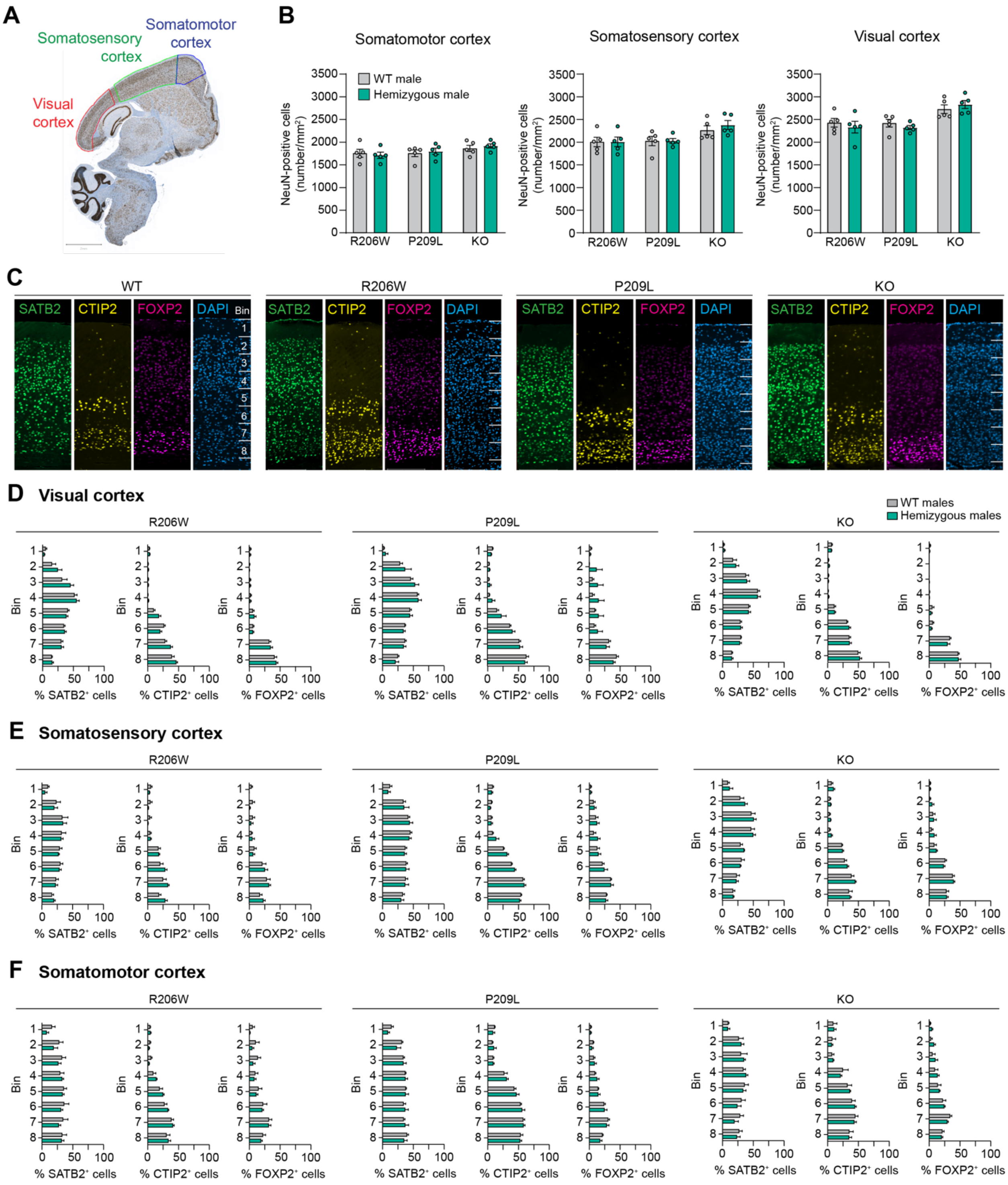

Supplemental Figure 9. Cortical neuronal count and distribution in *Hnrnph2* mice. (**A**) Immunohistochemistry with NeuN in a WT mouse brain with manual annotation of visual, somatosensory, and somatomotor cortex. (**B**) Quantification of neurons in somatomotor, somatosensory, and visual cortex. The number of NeuN-positive cells per mm^2 is shown. Error bars represent mean ± SEM. (**C**) Immunofluorescence with cortical layer-specific markers

SATB2 (layer II-IV), CTIP2 (layer V), and FOXP2 (layer VI) performed in WT, hnRNPH2 R206W, hnRNPH2 P209L, and *Hnrnph2* KO male mice. Regions of interest were positioned over the visual, somatomotor, and somatosensory cortex and subdivided into 8 equal bins. (**D–F**) The number of SATB2-, CTIP2-, and FOXP2-positive cells were counted and expressed as a percentage of the total number of DAPI-positive cells within each bin in the visual (**D**), somatosensory (**E**), and somatomotor cortex (**F**) as defined in panel (**C**). Error bars represent mean ± SEM. n = 5 per group.

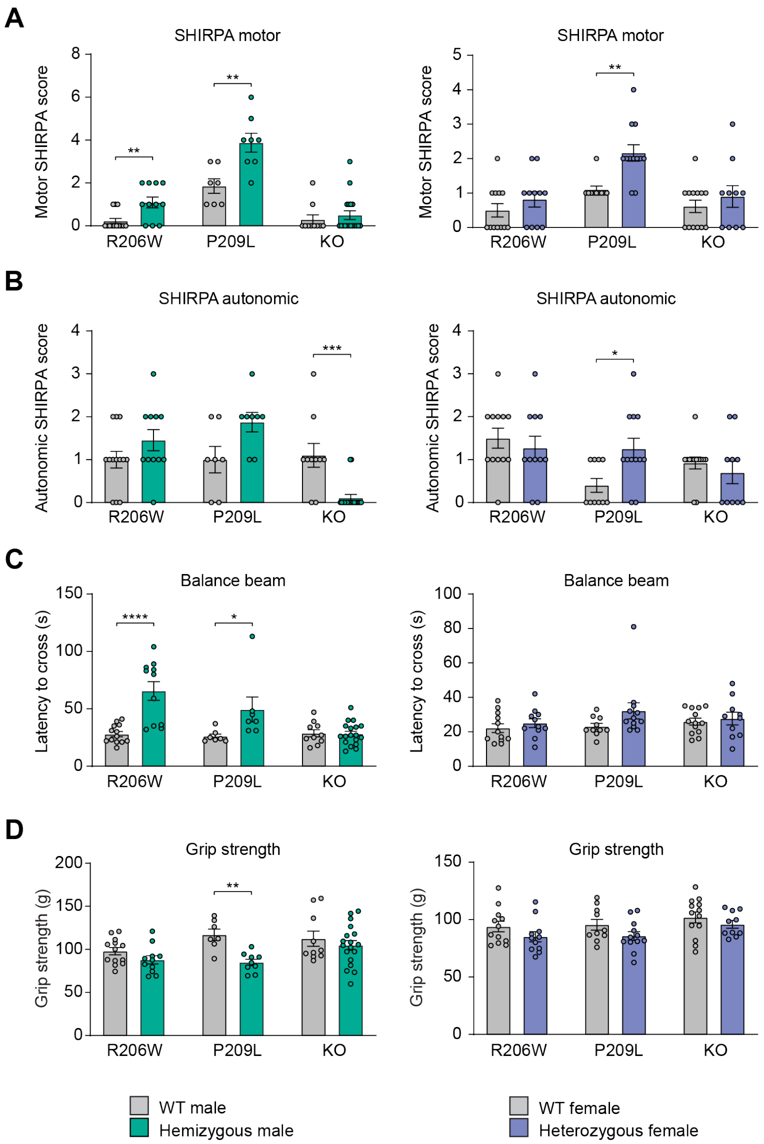

Supplemental Figure 10. *Hnrnph2* mutant mice have impaired motor function. (**A** and **B**) Subdomain SHIRPA scores are shown. Motor function (**A**): ***P* = 0.008 *Hnrnph2*$^{R206W/Y}$ vs. *Hnrnph2*$^{X/Y}$; ***P* = 0.008 *Hnrnph2*$^{P209L/Y}$ vs. *Hnrnph2*$^{X/Y}$; ***P* = 0.001 *Hnrnph2*$^{P209L/X}$ vs. *Hnrnph2*$^{X/X}$ by two-way non-parametric ANOVA with Mann-Whitney U test for group wise comparisons. Autonomic function (**B**): ****P* = 0.0003 *Hnrnph2*$^{KO/Y}$ vs. *Hnrnph2*$^{X/Y}$; **P* = 0.017 *Hnrnph2*$^{P209L/X}$ vs. *Hnrnph2*$^{X/X}$ by two-way non-parametric ANOVA with Mann-Whitney U test for group wise comparisons. Error bars represent mean ± SEM in all graphs. (**C**) Latency to cross balance beam, *****P* < 0.0001 *Hnrnph2*$^{R206W/Y}$ vs. *Hnrnph2*$^{X/Y}$; **P* = 0.0264 *Hnrnph2*$^{P209L/Y}$ vs. *Hnrnph2*$^{X/Y}$ by two-way ANOVA with Sidak's multiple comparisons test. Error bars represent mean ± SEM in all graphs. (**D**) Grip strength, ***P* = 0.0065 *Hnrnph2*$^{P209L/Y}$ vs. *Hnrnph2*$^{X/Y}$ by two-way ANOVA with Sidak's multiple comparisons test. Error bars represent mean ± SEM in all graphs. Group sizes for SHIRPA and motor tests were as follows: *Hnrnph2*$^{R206W/Y}$ (n = 11) vs. *Hnrnph2*$^{X/Y}$ (n = 13); *Hnrnph2*$^{P209L/Y}$ (n = 8) vs. *Hnrnph2*$^{X/Y}$ (n = 7); *Hnrnph2*$^{KO/Y}$ (n = 18) vs. *Hnrnph2*$^{X/Y}$ (n = 10); *Hnrnph2*$^{R206W/X}$ (n = 11) vs. *Hnrnph2*$^{X/X}$ (n = 12); *Hnrnph2*$^{P209L/X}$ (n = 12) vs. *Hnrnph2*$^{X/X}$ (n = 10); *Hnrnph2*$^{KO/X}$ (n = 10) vs. *Hnrnph2*$^{X/X}$ (n = 13).

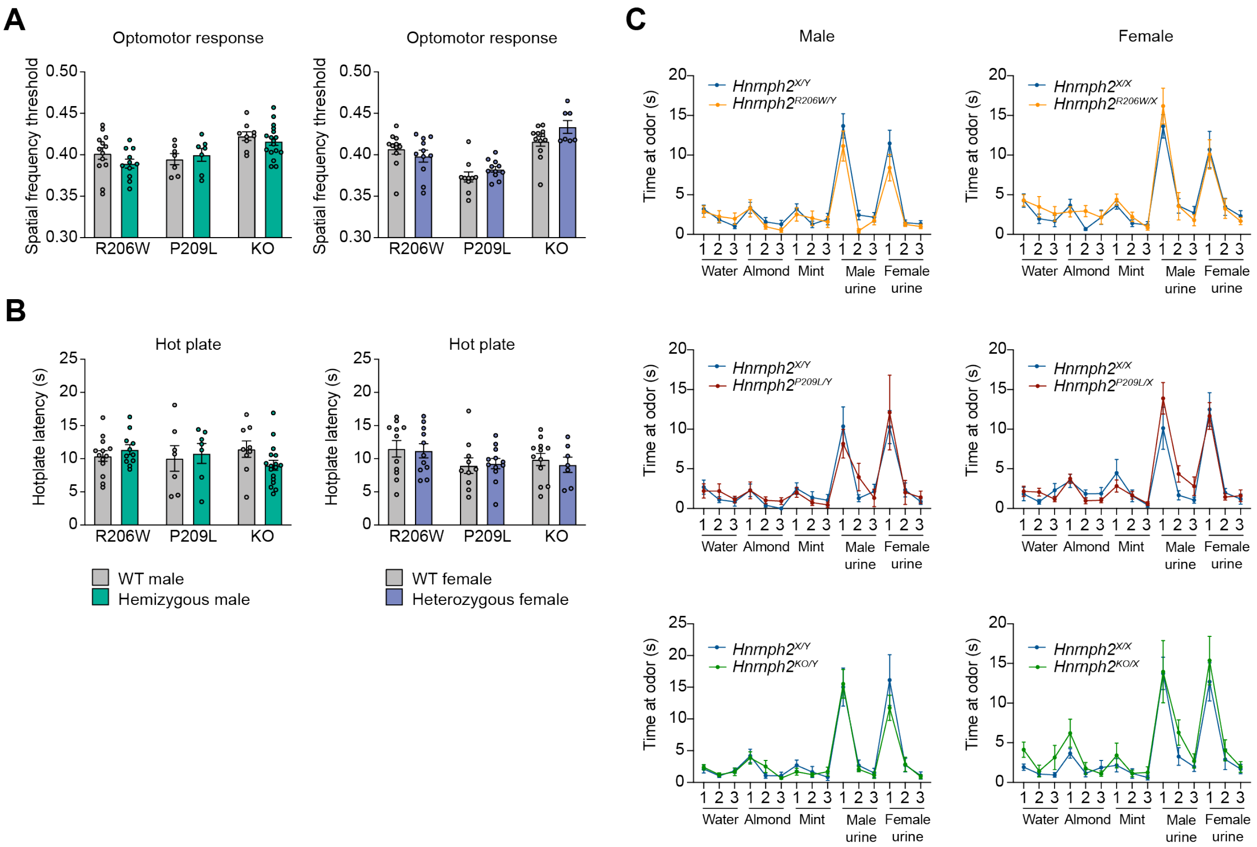

Supplemental Figure 11. *Hnrnph2* mutant and KO mice have normal sensory function. (**A**) Optomotor response test of visual acuity. (**B**) Hot plate test of pain response. (**C**) Scent habituation test of olfaction. Error bars represent mean ± SEM in all graphs. Group sizes were as follows: *Hnrnph2*$^{R206W/Y}$ (n = 11) vs. *Hnrnph2*$^{X/Y}$ (n = 13); *Hnrnph2*$^{P209L/Y}$ (n = 7) vs. *Hnrnph2*$^{X/Y}$ (n = 7); *Hnrnph2*$^{KO/Y}$ (n = 17) vs. *Hnrnph2*$^{X/Y}$ (n = 10); *Hnrnph2*$^{R206W/X}$ (n = 11) vs. *Hnrnph2*$^{X/X}$ (n = 12); *Hnrnph2*$^{P209L/X}$ (n = 11) vs. *Hnrnph2*$^{X/X}$ (n = 10); *Hnrnph2*$^{KO/X}$ (n = 7) vs. *Hnrnph2*$^{X/X}$ (n = 12).

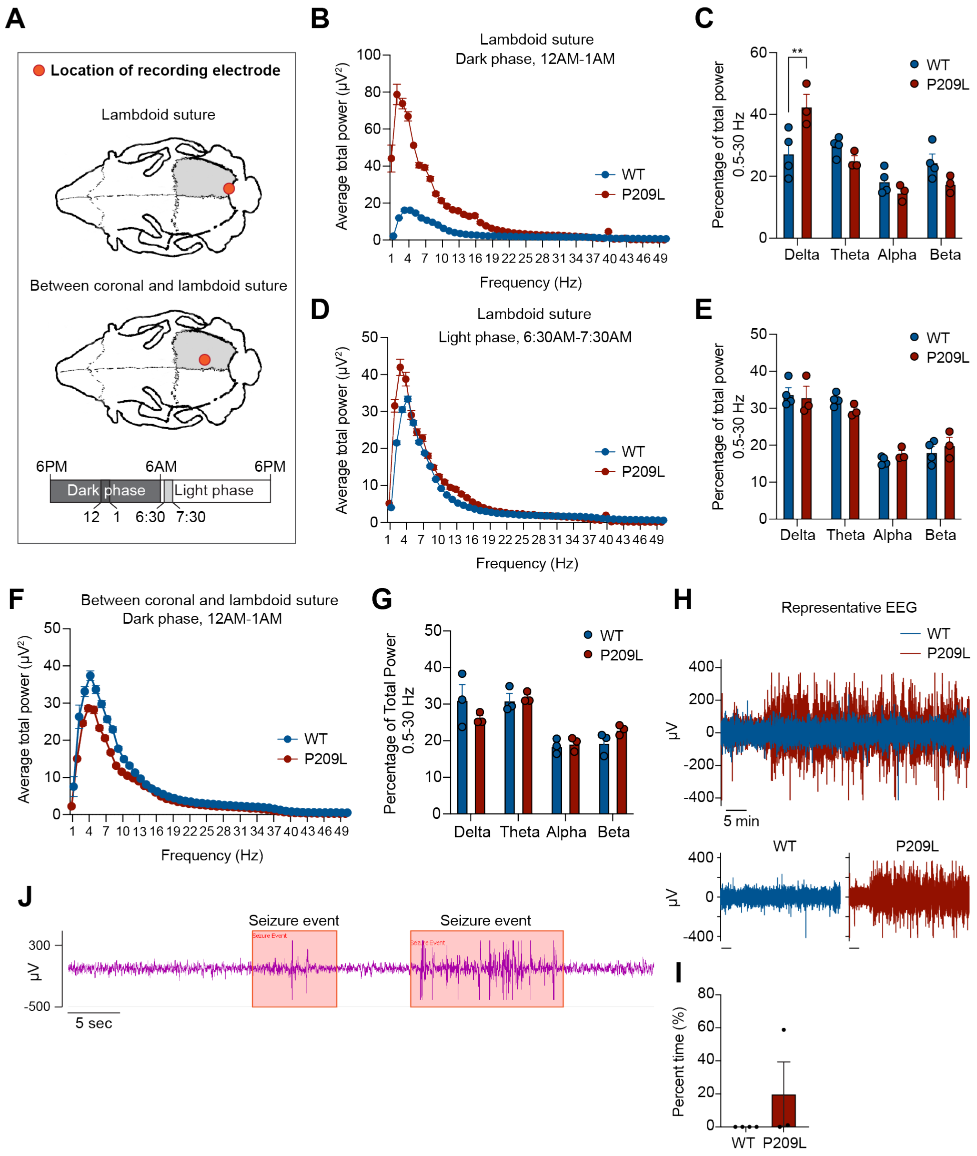

Supplemental Figure 12. Cortical EEG dynamics are altered during the dark phase in *Hnrnph2* mutant mice. (**A**) Schematic depicting location of EEG leads. Top: lambdoid suture; bottom*:* between coronal and lambdoid suture. Mice were monitored and analyzed for 24 hours. Representative 1-hour windows for the dark phase (12am-1am) and light phase (6:30am-7:30am) are presented. (**B, C**) Group analysis for a lead at the right lambdoid suture showing spectral power (**B**) and the relative power distribution across wavelength categories (**C**) from 12am to 1am. ***P* = 0.0021 by two-way ANOVA with Sidak's multiple comparisons test. (**D, E**) Group analysis for a lead at the right lambdoid suture showing average spectral power (**D**) and the relative power distribution across wavelength categories (**E**) from 6:30am to 7:30am. (**F, G**) Group analysis for a lead between the right coronal and lambdoid sutures showing average spectral power (**F**) and the relative power distribution across wavelength categories (**G**) from 12am to 1am. (**H**) Representative EEG activity in the lead at the right lambdoid suture from 12am to 1 am. (**I**) Percentage of time with epileptiform activity in the lead at the right lambdoid suture from 12am to 1 am. (**J**) EEG activity of P209L hemizygous male showing seizure-like behavior. Group sizes for EEG analysis were as follows: $Hnrnph2^{P209L/Y}$ (n = 3) vs. $Hnrnph2^{X/Y}$ (n = 4).

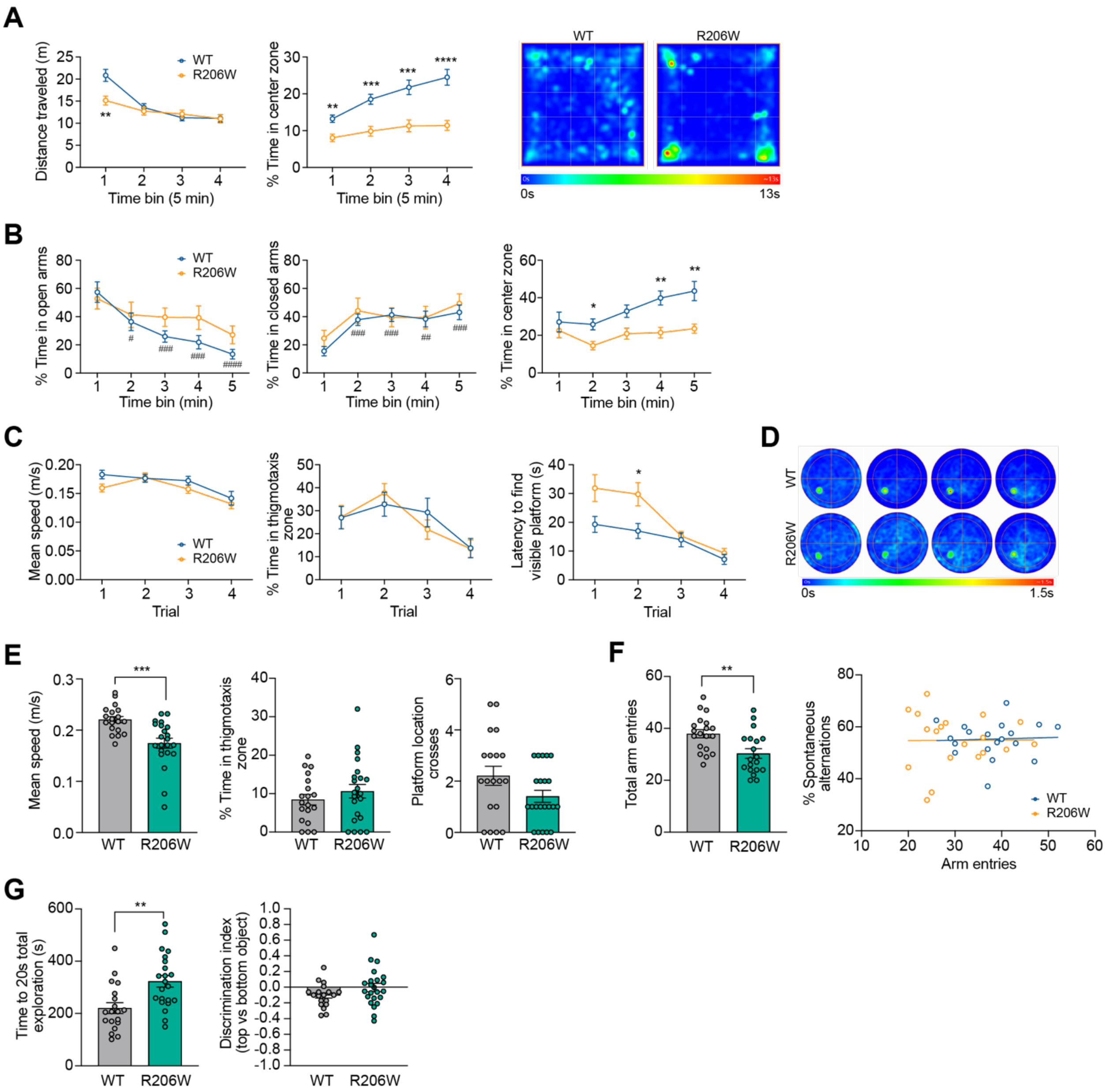

Supplemental Figure 13. hnRNPH2 R206W males have increased anxiety, impaired spatial learning and memory, deficits in social interaction, and reduced marble burying. (**A**) Distance traveled (time bin 1 $^{**}P = 0.0069$ $Hnrnph2^{R206W/Y}$ (n = 22) vs. $Hnrnph2^{X/Y}$ (n = 19)) and percentage time spent in the center zone (time bin 1 $^{**}P = 0.0031$, time bin 2 $^{***}P = 0.0002$, time bin 3 $^{***}P = 0.0009$, time bin 4 $^{****}P < 0.0001$ $Hnrnph2^{R206W/Y}$ vs. $Hnrnph2^{X/Y}$) of the open field test across 5-minute bins by two-way ANOVA with Sidak's multiple comparisons test. At right is an averaged heat map of the animals' center point for the groups for the entire duration of the 20-minute test. (**B**) Percentage time spent in the open arms (time bin 2 $^{\#}P = 0.0359$, time bin 3 $^{\#\#\#}P = 0.0004$, time bin 4 $^{\#\#\#}P = 0.0004$, time bin 5 $^{\#\#\#\#}P < 0.0001$ vs. time bin 1 for $Hnrnph2^{X/Y}$ by two-way ANOVA with Dunnett's multiple comparisons test), closed arms (time bin 2 $^{\#\#\#}P = 0.0002$, time bin 3 $^{\#\#\#}P = 0.0006$, time bin 4 $^{\#\#}P = 0.0042$, time bin 5 $^{\#\#\#}P = 0.0004$ vs. time bin 1 for $Hnrnph2^{X/Y}$ by two-way ANOVA with Dunnett's multiple comparisons test), and center zone (time bin 2 $^{*}P = 0.02$, time bin 4 $^{**}P = 0.0024$, time bin 5 $^{**}P = 0.0087$ $Hnrnph2^{R206W/Y}$ (n = 13) vs. $Hnrnph2^{X/Y}$ (n = 18) by two-way ANOVA with Sidak's multiple

comparisons test) of the elevated plus maze across 1-minute bins. (**C**) Mean speed, percentage time spent in the thigmotaxis zone, and latency to find the visible platform (trial 2 **P* = 0.0467 *Hnrnph2*$^{R206W/Y}$ (n = 22) vs. *Hnrnph2*$^{X/Y}$ (n = 19) by two-way ANOVA with Sidak's multiple comparisons test) in the cued trials of the Morris water maze. (**D**) Averaged heat map of the animals' center point for the groups for training day 1-4 of the Morris water maze. (**E**) Mean speed (****P* = 0.0004 *Hnrnph2*$^{R206W/Y}$ (n = 22) vs. *Hnrnph2*$^{X/Y}$ (n = 19) by unpaired t test), percentage time spent in the thigmotaxis zone, and number of platform location crossings during the probe trial of the Morris water maze. (**F**) Number of total arm entries (***P* = 0.0034 *Hnrnph2*$^{R206W/Y}$ (n = 20) vs. *Hnrnph2*$^{X/Y}$ (n = 19) by unpaired t test) and lack of correlation between percentage spontaneous alternations and number of total arm entries by Pearson's correlation analysis. (**G**) Time to reach 20 seconds of total object exploration (***P* = 0.0019 *Hnrnph2*$^{R206W/Y}$ (n = 22) vs. *Hnrnph2*$^{X/Y}$ (n = 19) by unpaired t test) and object position discrimination index in the familiarization stage of the novel object recognition test.

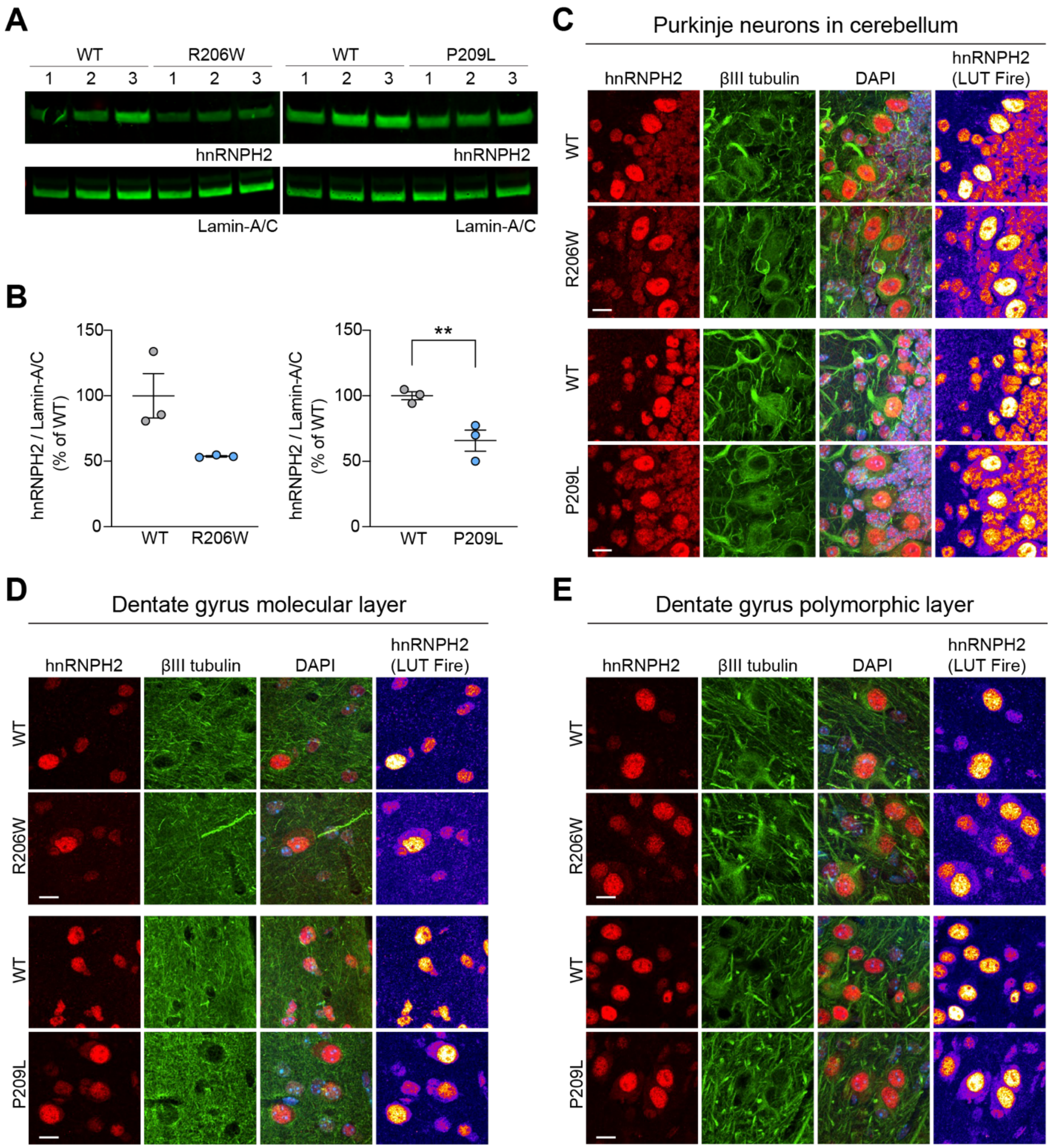

Supplemental Figure 14. Pathogenic variants alter the nucleocytoplasmic ratio of hnRNPH2 in hnRNPH2 P209L and R206W mice. (**A**) Immunoblot of hnRNPH2 in cortical nuclear fractions. Lamin A/C was used as a loading control; labels 1-3 indicate three biological replicates. (**B**) Quantification of hnRNPH2 normalized to lamin A/C; error bars represent mean ± SEM. $^{**}P$ = 0.0063 *Hnrnph2*$^{P209L/Y}$ vs. *Hnrnph2*$^{X/Y}$ by two-way ANOVA with Sidak's multiple comparisons test. n = 3 per group. (**C-E**) Immunofluorescent staining of hnRNPH2 in mouse brain sections. Purkinje neurons in cerebellum (**C**), molecular layer (**D**) and polymorphic layer (**E**) of dentate gyrus are shown. βIII tubulin and DAPI were used as neuronal cytoplasmic and nuclear markers, respectively. Look-up table (LUT) fire was used to increase the visibility of the hnRNPH2 cytoplasmic signal. Scale bars, 10 μm.

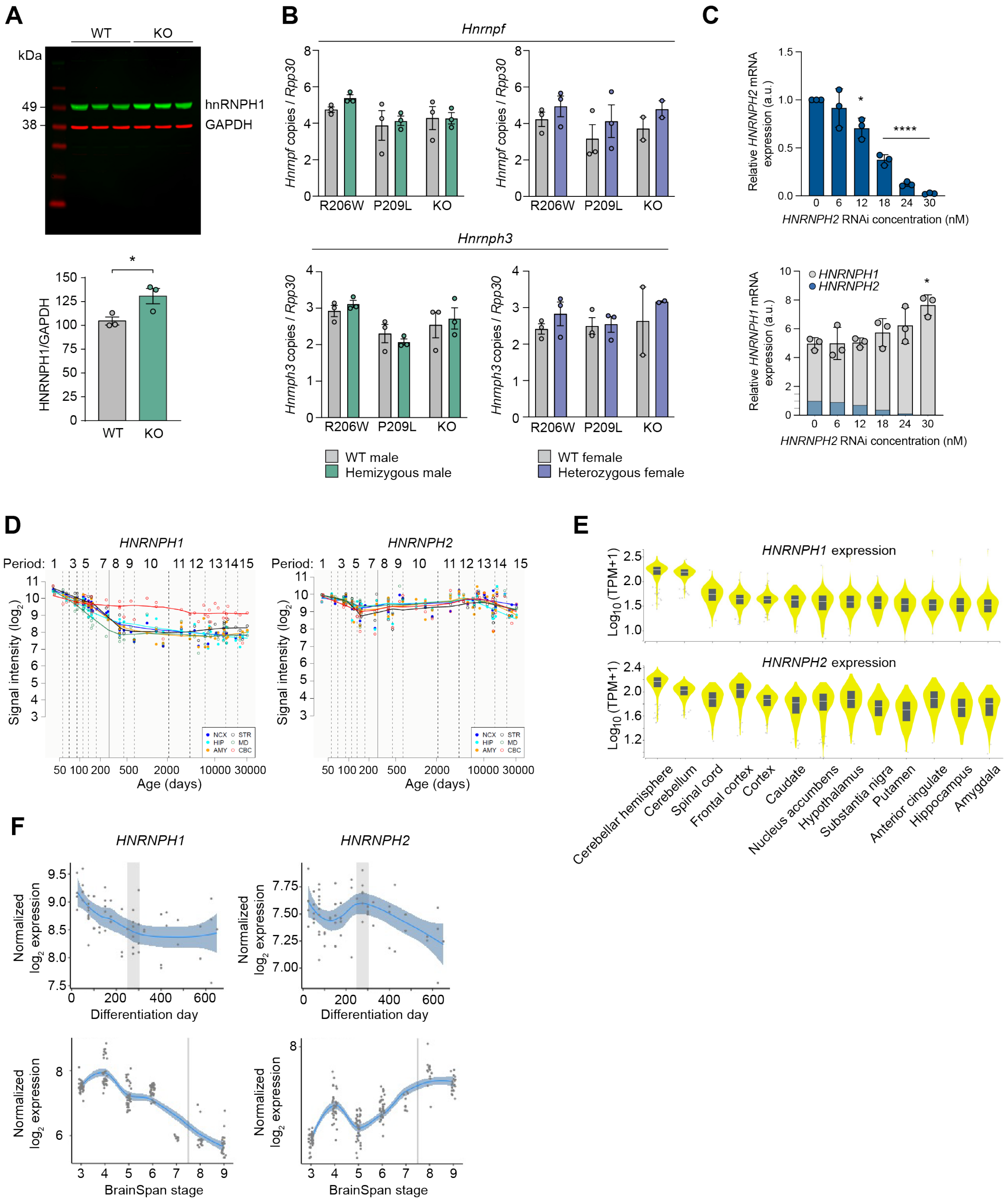

Supplemental Figure 15. Spatiotemporal expression of *HNRNPH1* and *HNRNPH2* in human brain and cortical organoids. (**A**) Immunoblot and quantification of hnRNPH1 expression in whole brain RIPA-soluble fractions. GAPDH was used as a loading control; **P* = 0.0454 WT vs. *Hnrnph2* KO by t-test. Error bars represent mean ± SEM. n = 3 per group. (**B**) Number of *Hnrnpf* and *Hnrnph3* copies normalized to *Rpp30* in the cortex of *Hnrnph2*^{R206W}, *Hnrnph2*^{P209L}, and *Hnrnph2*^{KO} mice by ddRT-PCR. Error bars represent mean ± SEM. n = 3 per

group, except for *Hnrnph2*$^{KO/X}$ and *Hnrnph2*$^{X/X}$ which are n=2 per group. (**C**) Relative expression levels of *HNRNPH1* and *HNRNPH2* in HEK293T cells transfected with siRNA targeting *HNRNPH2* by qRT-PCR. To show the relative expression of *HNRNPH1* and *HNRNPH2*, the *HNRNPH2* graph is overlaid on the *HNRNPH1* graph with an adjusted scale. *P = 0.0107 (*HNRNPH2*), 0.0106 (*HNRNPH1*), and ****P < 0.0001 by one-way ANOVA with Dunnett's multiple comparisons test. Error bars represent mean ± SD. (**D**) Trajectory plots showing the expression of *HNRNPH1* and *HNRNPH2* in 6 major brain regions across 15 developmental time points by Affymetrix GeneChip Human Exon 1.0 ST Arrays. Period 1-7, fetal development; solid line, birth; period 8-9, infancy; period 10-11, childhood; period 12, adolescence; period 13-15, adulthood. Neocortex (NCX), hippocampus (HIP), amygdala region (AMY), striatum (STR), mediodorsal nucleus of the thalamus (MD), and cerebellar cortex (CBC) are shown. Reprinted from the Human Brain Transcriptome dataset (1-3). (**E**) Violin plots showing the expression of *HNRNPH1* and *HNRNPH2* in 13 brain regions by RNA-seq. Data used for the analyses described here were obtained from the Genotype-Tissue Expression (GTEx) Portal, dbGaP accession number: phs000424.v8.p2. TPM, transcripts per million. (**F**) Trajectory plots showing the expression of *HNRNPH1* and *HNRNPH2* in human cortical organoids across differentiation day and mapped BrainSpan stages by RNA-seq. Stage 3-7, fetal development; stage 8, birth to 6 months; stage 9, 6 months to 19 months. Transition from prenatal to postnatal stages is indicated with a vertical grey area/line. Reprinted from the Gene Expression in Cortical Organoids dataset (4).

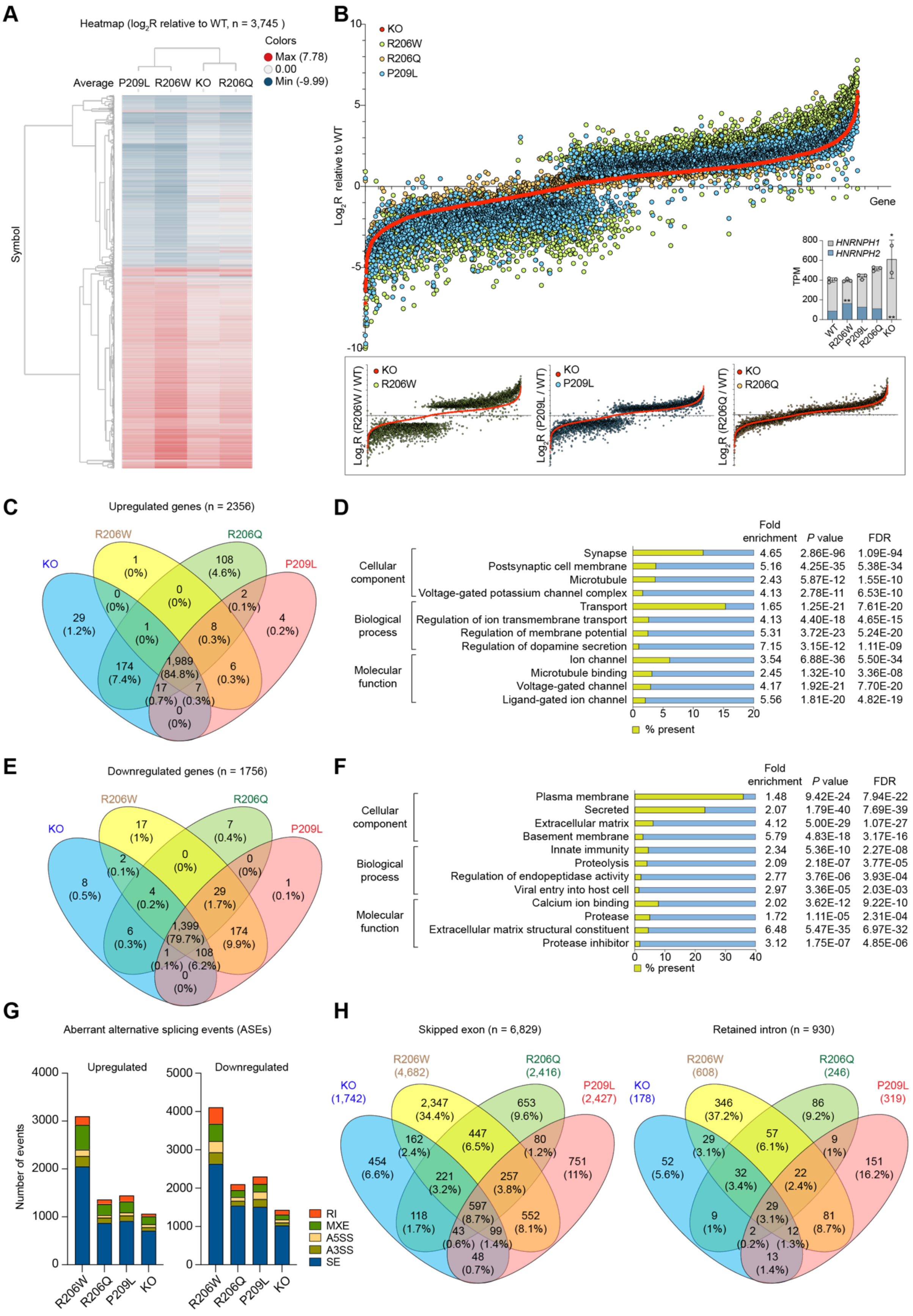
A
Heatmap (log2R relative to WT, n = 3,745)
Colors
Max (7.78)
0.00
Min (-9.99)
Average P209L R206W KO R206Q
Symbol
B
KO
R206W
R206Q
P209L
Log2R relative to WT
Gene
HNRNPH1
HNRNPH2
TPM
WT R206W P209L R206Q KO
Log2R (R206W / WT)
Log2R (P209L / WT)
Log2R (R206Q / WT)
C
Upregulated genes (n = 2356)
KO
R206W
R206Q
P209L
1 (0%)
108 (4.6%)
0 (0%)
0 (0%)
2 (0.1%)
29 (1.2%)
1 (0%)
8 (0.3%)
4 (0.2%)
1,989 (84.8%)
174 (7.4%)
6 (0.3%)
17 (0.7%)
7 (0.3%)
0 (0%)
D
Fold enrichment
P value
FDR
Cellular component
Synapse 4.65 2.86E-96 1.09E-94
Postsynaptic cell membrane 5.16 4.25E-35 5.38E-34
Microtubule 2.43 5.87E-12 1.55E-10
Voltage-gated potassium channel complex 4.13 2.78E-11 6.53E-10
Biological process
Transport 1.65 1.25E-21 7.61E-20
Regulation of ion transmembrane transport 4.13 4.40E-18 4.65E-15
Regulation of membrane potential 5.31 3.72E-23 5.24E-20
Regulation of dopamine secretion 7.15 3.15E-12 1.11E-09
Molecular function
Ion channel 3.54 6.88E-36 5.50E-34
Microtubule binding 2.45 1.32E-10 3.36E-08
Voltage-gated ion channel 4.17 1.92E-21 7.70E-20
Ligand-gated ion channel 5.56 1.81E-20 4.82E-19
% present
E
Downregulated genes (n = 1756)
KO
R206W
R206Q
P209L
17 (1%)
7 (0.4%)
2 (0.1%)
0 (0%)
0 (0%)
8 (0.5%)
4 (0.2%)
29 (1.7%)
1 (0.1%)
1,399 (79.7%)
6 (0.3%)
174 (9.9%)
1 (0.1%)
108 (6.2%)
0 (0%)
F
Fold enrichment
P value
FDR
Cellular component
Plasma membrane 1.48 9.42E-24 7.94E-22
Secreted 2.07 1.79E-40 7.69E-39
Extracellular matrix 4.12 5.00E-29 1.07E-27
Basement membrane 5.79 4.83E-18 3.17E-16
Biological process
Innate immunity 2.34 5.36E-10 2.27E-08
Proteolysis 2.09 2.18E-07 3.77E-05
Regulation of endopeptidase activity 2.77 3.76E-06 3.93E-04
Viral entry into host cell 2.97 3.36E-05 2.03E-03
Molecular function
Calcium ion binding 2.02 3.62E-12 9.22E-10
Protease 1.72 1.11E-05 2.31E-04
Extracellular matrix structural constituent 6.48 5.47E-35 6.97E-32
Protease inhibitor 3.12 1.75E-07 4.85E-06
% present
G
Aberrant alternative splicing events (ASEs)
Upregulated
Downregulated
Number of events
R206W R206Q P209L KO
RI
MXE
A5SS
A3SS
SE
H
Skipped exon (n = 6,829)
KO (1,742)
R206W (4,682)
R206Q (2,416)
P209L (2,427)
2,347 (34.4%)
653 (9.6%)
162 (2.4%)
447 (6.5%)
80 (1.2%)
454 (6.6%)
221 (3.2%)
257 (3.8%)
751 (11%)
597 (8.7%)
118 (1.7%)
552 (8.1%)
43 (0.6%)
99 (1.4%)
48 (0.7%)
Retained intron (n = 930)
KO (178)
R206W (608)
R206Q (246)
P209L (319)
346 (37.2%)
86 (9.2%)
29 (3.1%)
57 (6.1%)
9 (1%)
52 (5.6%)
32 (3.4%)
22 (2.4%)
151 (16.2%)
29 (3.1%)
9 (1%)
81 (8.7%)
2 (0.2%)
12 (1.3%)
13 (1.4%)

Supplemental Figure 16. RNA sequencing of human iPSC-derived neurons harboring a pathogenic mutation or deletion of *HNRNPH2*. (**A**) Heatmap of differentially expressed genes from 3-week-old neurons comparing WT (n = 3), hnRNPH2 R206W (n = 3), hnRNPH2 R206Q (n = 3), and *HNRNPH2* KO (n = 2). Colors of bars represent $\log_2$ ratio normalized to WT and scaled expression levels. Red, white, and blue correspond to high (max = 7.78), 0, and low (min = -9.99) expression, respectively. (**B**) Log_2 ratio (relative to WT) for each genotype is plotted for individual genes (X-axis). Inset bar graphs show *HNRNPH1* and *HNRNPH2* transcript levels in each genotype. $^{**}P = 0.0024$ (R206W) and $^{**}P = 0.0026$ (KO) by one-way ANOVA with Dunnett's multiple comparisons test. Error bars represent mean ± SD. (**C**) Venn diagram showing the overlap of genes upregulated in each genotype. (**D**) Gene ontology (GO) analysis of commonly upregulated genes in mutant and KO neurons (1,989 genes). (**E**) Venn diagram showing the overlap of genes downregulated in each genotype. (**F**) GO analysis of commonly downregulated genes in mutant and KO neurons (1,399 genes). (**G**) Graphs summarizing aberrant alternative splicing events. Numbers of upregulated and downregulated events are shown separately. RI: retained intron, MXE: mutually exclusive exons, A5SS: alternative 5′ splice sites, A3SS: alternative 3′ splice sites, SE: skipped exons. (**H**) Venn diagrams showing the overlap of skipped exon and retained intron events in each genotype.

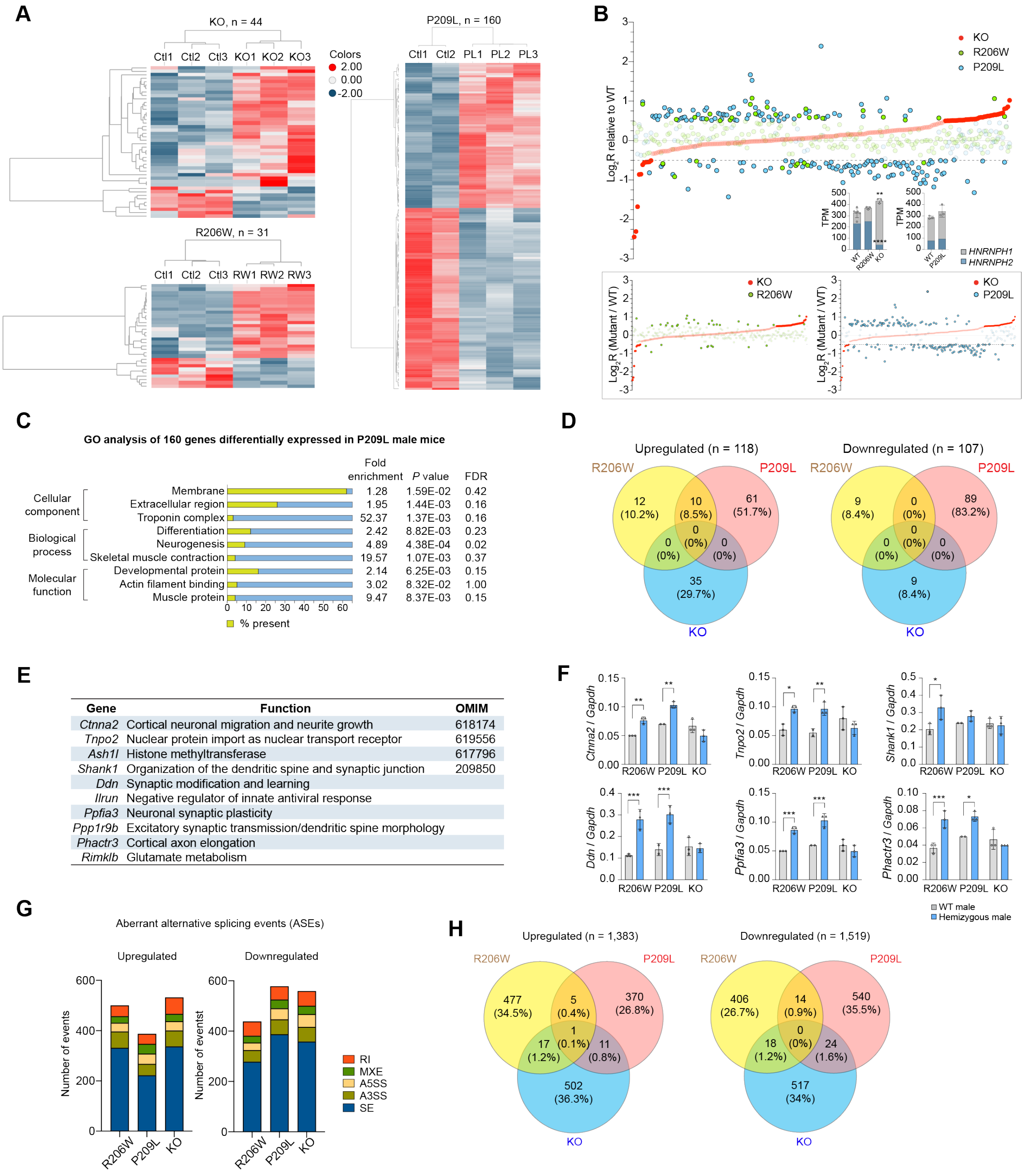

Supplemental Figure 17. RNA sequencing of cortices from mice harboring a pathogenic mutation or deletion of *Hnrnph2*. (**A**) Heatmap of differentially expressed genes in cortices from 8-week-old KO (n = 3) and R206W male mice (n = 3) and 3-week-old P209L male mice (n = 3). Littermate WT male mice (n = 3, except for WT males from the P209L line which was n = 2)) were used as controls. Colors of bars represent $\log_2$ ratio normalized to WT and scaled expression levels. Red, white, and blue correspond to high (max = 2.00), 0, and low (min = -2.00) expressions, respectively. (**B**) Log_2 ratio (mutant / WT) for each mutant is plotted for

individual genes (X-axis). Inset bar graphs show *HNRNPH1* and *HNRNPH2* transcript levels in each genotype. ****$P < 0.0001$ (*Hnrnph2* in KO) and **$P = 0.0029$ (*Hnrnph1* in KO) by one-way ANOVA with Dunnett's multiple comparisons test. Error bars represent mean ± SD. (**C**) Gene ontology (GO) analysis of differentially regulated genes in P209L male mice compared to littermate controls (536 genes). (**D**) Venn diagrams showing the overlap of genes upregulated and downregulated in each genotype. (**E**) Table listing 10 commonly upregulated genes in R206W and P209L mice and their known neuron-related functions. (**F**) ddRT-PCR analyses validating upregulation of subset of genes listed in (**E**). For *Ctnna2*, **$P = 0.0031$ (R206W), **$P = 0.0013$ (P209L); for *Tnpo2*, *$P = 0.0111$ (R206W), **$P = 0.0100$ (P209L); for *Shank1*, *$P = 0.0128$ (R206W); for *Ddn,* ***$P = 0.0003$ (R206W), ***$P = 0.0008$ (P209L); for *Ppfia3*, ***$P = 0.0006$ (R206W), ***$P = 0.0004$ (P209L); for *Phactr3*, ***$P = 0.0005$ (R206W), *$P = 0.0159$ (P209L) by two-way ANOVA with Sidak's multiple comparisons test. Error bars represent mean ± SD. n = 3 per group, except for WT males from the P209L line which was n = 2. (**G**) Graphs summarizing aberrant alternative splicing events. Numbers of upregulated and downregulated events are shown separately. RI: retained intron, MXE: mutually exclusive exons, A5SS: alternative 5′ splice sites, A3SS: alternative 3′ splice sites, SE: skipped exons. (**H**) Venn diagrams showing the overlap of upregulated and downregulated ASEs in each genotype.

Supplemental Table Legends

Supplemental Table 1. Regional brain volumes normalized to total brain tissue volume in mice harboring a pathogenic mutation or deletion of *Hnrnph2*. Group sizes for brain volume analysis were as follows: *Hnrnph2*$^{R206W/Y}$ (n = 9) vs. *Hnrnph2*$^{X/Y}$ (n = 7); *Hnrnph2*$^{KO/Y}$ (n = 11) vs. *Hnrnph2*$^{X/Y}$ (n = 9); *Hnrnph2*$^{R206W/X}$ (n = 16) vs. *Hnrnph2*$^{X/X}$ (n = 11); *Hnrnph2*$^{P209L/X}$ (n = 7) vs. *Hnrnph2*$^{X/X}$ (n = 12); *Hnrnph2*$^{KO/X}$ (n = 12) vs. *Hnrnph2*$^{X/X}$ (n = 14).

Supplemental Table 2. RNA composition of human iPSC-derived neurons harboring a pathogenic mutation or deletion of HNRNPH2.

Supplemental Table 3. RNA composition of cortices from male mice harboring a pathogenic mutation or deletion of *Hnrnph2*.

Supplemental Table 4. Exact P values from statistical comparisons shown in Figures 1-8.

Supplemental Table 5. CRISPR-Cas9 editing construct sequences.

**Name**	**Sequence (5’ to 3’)**
sgRNA Spacers	
hnRNPH2 Deletion	
SS73.HNRNPH2.g21	CAUUUCAGUUAUGUGCCCUA
SS74.hHNRNPH2.g6	CAGGACUAUUGAGCUACUUU
hnRNPH2 R206 and P209 point mutation	
SNP211.hnRNPH2.g14	CCCGGCCUAUCAUAGGGACC
Primers for PCR and Deep Sequencing Validation (partial Illumina adaptors in uppercase)	
hnRNPH2 Deletion	
SS73.hhnRNPH2.DS.F	CTACACGACGCTCTTCCGATCT tgtgctcttgggtcacttacaa
SS74 hhnRNPH2.ds.R	CAGACGTGTGCTCTTCCGATCT gtgccttctagtttgtggtgaaataa
hnRNPH2 Internal primers to ensure complete knockout	
SS73.internal.F2	gggctggcagagggtataat
SS73.internal.R2	gtggtgctctggaaactgga
hnRNPH2 R206 and P209 point mutation lines	
SNP211.DS.F	CTACACGACGCTCTTCCGATCTgcctttgtgcagtttgcttcacagg
SNP211.DS.R	CAGACGTGTGCTCTTCCGATCTtggtgctctggaaactggacccacc
Donor sequences	
hnRNPH2 R206W ssODN donor	
SNP211.hhnRNPH2.g14.block_mod_R206W.sense<br><br>*AltR™ modifications<br><br>Modifications in upper case	*agtagccgagctgaagttcgaacccactatgatccccctcgaaagctcatggctatgcagTggccaggCccTtatgataggccgggggctggcagagggtataatagcattggcagaggagctgggtttgaaagg
hnRNPH2 R206Q ssODN donor	
SNP211.hhnRNPH2.g14.block_mod_R206Q.sense<br><br>*AltR™ modifications<br><br>Modifications in upper case	*agtagccgagctgaagttcgaacccactatgatccccctcgaaagctcatggctatgcagcAgccaggCccTtatgataggccgggggctggcagagggtataatagcattggcagaggagctgggtttgaaagg
hnRNPH2 P209L ssODN donor	
SNP211.hhnRNPH2.g14.block_mod_P209L.anti	*ttcaaacccagctcctctgccaatgctattataccctctgccagcccccggcctatcataCAgacctggccgctgcatagccatgagctttcgagggggatcatagtgggttcgaacttcagc
hnRNPH2 g14 blocking ssODN donor	
SNP211.hhnRNPH2.g14.block.anti<br>*AltR™ modifications<br>Blocking mutations in upper case	*cctttcaaacccagctcctctgccaatgctattataccctctgccagcccccggcctatcataAggGcctggccgctgcatagccatgagctttcgagggggatcatagtgggttcgaacttcagctcggctact

Supplemental Methods

Cell culture and transfection

HEK293T (CRL-3216) and HeLa (CCL-2) cells were originally purchased from ATCC and periodically authenticated by short tandem repeat (STR) profiling. Cells were grown in Dulbecco's modified Eagle's medium (DMEM) supplemented with 10% fetal bovine serum (FBS), 1% penicillin/streptomycin, and 1% L-glutamate. Cells were counted using ADAM-CellT (NanoEntek), plated and transfected using Lipofectamine 3000 (Thermo Fisher; L3000008) for transient overexpression or RNAiMAX (Thermo Fisher; 13778075) for siRNA knockdown according to the manufacturer's instructions.

Generation of human iPSCs bearing hnRNPH2 mutations

Genetically modified AN1.1 iPSC lines were generated using CRISPR-Cas9 technology. Briefly, for each modification, unmodified AN1.1 iPSCs were pretreated for 1 hour in StemFlex (Thermo Fisher Scientific) supplemented with 1X RevitaCell (Thermo Fisher Scientific). After pretreatment, approximately, $1X10^6$ cells were nucleofected (Lonza, 4D-Nucleofector™ X-unit) with precomplexed ribonuclear proteins (RNPs) consisting of 120 pmol of chemically modified sgRNA (Synthego), 40 pmol of Cas9 protein (St. Jude Protein Production Core), 40ng of pCXLE-EGFP (Addgene Plasmid #27082), and, when required, 1.5 ug of ssODN donor and 1.5ug of blocking ssODN donor in a small (20ul) cuvette using solution P3 and program CA-137 according to the manufacturer's recommended protocol. Cells were sorted five days post nucleofection for single cells by FACS at the Flow Cytometry and Cell Sorting Shared Resource (St. Jude) for transfected (GFP+) cells. Clones were plated into prewarmed (37C) StemFlex media supplemented with 1X CloneR (Stem Cell Technologies) into Vitronectin XF (Stem Cell Technologies) coated 96-well plates. Clones were screened for the desired modification via targeted deep sequencing on a Miseq Illumina sequencer as previously described (5). Samples

were demultiplexed using the index sequences, fastq files were generated, and NGS analysis of clones was performed using CRIS.py (6). Correctly modified clones were identified, expanded, and sequence confirmed. Cell identity was authenticated using the PowerPlex® Fusion System (Promega) performed at the Hartwell Center (St. Jude) and tested negative for mycoplasma by the MycoAlertTMPlus Mycoplasma Detection Kit (Lonza). Editing construct sequences and relevant primers are listed in Supplemental Table 5.

Differentiation of iPSC-derived neurons

iPSCs were differentiated into cortical neurons with a two-step protocol (pre-differentiation and maturation) as previously described (7). When iPSCs reached 70-80% confluence, cells were washed twice with DPBS and dissociated with Accutase (STEMCELL Technologies) and collected cells were filtered using a cell strainer (STEMCELL Technologies). Filtered cells were centrifuged at 200 rcf for 5 min at room temperature and pellets were resuspended with medium containing N2 supplement, non-essential amino acids (NEAA), GlutaMAX Supplement and Y-27632 (STEMCELL Technologies) and 1 µg/ml doxycycline hyclate (Sigma Aldrich). Cell counting was performed using 10 µl and a Countess cell counter (Thermo Fisher). Cells were subplated at 1.2 x 10^6 cells/well in 6-well dishes coated with Matrigel in knockout Dulbecco's modified Eagle's medium (KO-DMEM)/F12. The medium was changed daily for 3 days, and Y-27632 was removed from day 2. For maturation, pre-differentiated precursor cells were washed, dissociated, counted, and subplated at 25 x 10^4 cells/ml on dishes coated with 50 µg/ml poly-L-ornithine in BrainPhys neuronal medium (STEMCELL Technologies) containing N2 (Thermo Fisher Scientific), B-27, 20 ng/ml BDNF (PeproTech), 20 ng/ml GDNF (PeproTech), 500 µg/ml dibutyryl cyclic-AMP (Sigma Aldrich), 200 nM L-ascorbic acid (Sigma Aldrich), 1 µg/ml natural mouse laminin (Thermo Fisher Scientific), 1 µM AraC (Sigma Aldrich) and 1 µg/ml doxycycline hyclate. Half-medium was changed every other day.

Immunofluorescence and microscopy in human cell lines

HeLa cells were seeded on 8-well glass slides (Millipore). Twenty-four hours post transfection for overexpression or 72 hours post transfection for siRNA knockdown, cells were stressed with 500 μM sodium arsenite (Sigma-Aldrich) for times as indicated in text and legends. Cells were then fixed with 4% paraformaldehyde (Electron Microscopy Sciences), permeabilized with 0.5% Triton X-100, and blocked in 5% bovine serum albumin (BSA). Primary antibodies used were mouse monoclonal anti-FLAG (M2, F1804; Sigma), goat polyclonal anti-eIF3η (sc-16377; Santa Cruz Biotechnology), rabbit monoclonal anti-hnRNPH2 (ab179439; Abcam), mouse monoclonal anti-G3BP (611126; BD Biosciences), and mouse monoclonal anti-hnRNPA1 (05-1521; Millipore). For visualization, the appropriate host-specific Alexa Fluor 488, 555, or 647 (Invitrogen) secondary antibody was used. Slides were mounted using Prolong Gold Antifade Reagent with DAPI (Life Technologies). Images were captured using a Leica TCS SP8 STED 3X confocal microscope (Leica Biosystems) with a 63x objective. Fluorescent images were subjected to automated compartmentalization analysis using CellProfiler software (Broad Institute). Cells were segmented using DAPI and eIF3η channels to identify the nucleus and cytoplasm. Integrated intensity of nucleus, cytoplasm, and cells were measured. Percent cytoplasmic signal was calculated with the integrated cytoplasmic signal over the integrated cell signal.

Immunoprecipitation and Western blot analysis in cell lines

Cell lysates were prepared by lysing cells in buffer containing 20 mM phosphate buffer pH 7.5, 150 mM NaCl, 0.2% Triton X-100, and 10% glycerol with complete protease inhibitor cocktail (Clontech Laboratories). Cells were incubated on ice for 20 minutes before centrifugation at 14,000 rpm at 4°C. The resulting supernatant was pre-treated with EZview Red Protein A agarose beads (P6486; Sigma) for 45 minutes to reduce the likelihood of nonspecific binding to the agarose, and the beads were removed. EZview Red Anti-FLAG M2 agarose beads (F2426;

Sigma) were then added to the pre-treated lysates and incubated at 4°C for 2 hours. The agarose beads were washed three times with buffer above to remove any remaining nonspecific binding. Samples were eluted with FLAG peptide (F3290; Sigma) at a final concentration of 100 µg/ml for 30 minutes at vortex setting 5 (Scientific Industries) at 4°C. Samples were boiled in 1x LDS sample buffer (Thermo Fisher). Samples were resolved by electrophoresis on NuPAGE Novex 4-12% Bis-Tris gels (Invitrogen). Gels were transferred to nitrocellulose using an iBlot 2 gel transfer device (Thermo Fisher) and blocked in 5% BSA. Primary antibodies used were rabbit polyclonal anti-FLAG (F7425; Sigma) and mouse monoclonal anti-Kapβ2 (ab10303; Abcam). Blots were subsequently incubated with IRDye fluorescence antibodies (LI-COR) and protein bands were visualized using the Odyssey Fc system (LI-COR) and Image Studio (LI-COR). Bands were quantified by densitometry in ImageJ (NIH). The full, uncut gels are included in the Supplemental Material.

Pulldown assays for Kapβ2 binding to immobilized GST-hnRNPH2 peptides

E. coli (BL21) transformed with pGEX-4TT3 plasmids expressing GST-hnRNPH2 proteins were grown in 35 ml LB with 100 µg/ml ampicillin to OD_{600} 0.6. Protein expression was then induced with 0.5 mM isopropyl-β-d-1-thiogalactoside (IPTG) for 5 hours at 37°C. Cells were harvested by centrifugation, resuspended in lysis buffer (50 mM Tris pH 7.4, 150 mM NaCl, 1 mM EDTA, 2 mM DTT, 15% glycerol, and protease inhibitors), lysed by sonication, the lysate centrifuged, and supernatant containing GST-hnRNPH2 proteins added to Glutathione Sepharose 4B (GSH; GE Healthcare) beads. The beads with immobilized GST-hnRNPH2 proteins were washed with lysis buffer. 50 µl beads containing ~60 µg immobilized GST-hnRNPH2 proteins were incubated with 8 µM Kapβ2 in 100 µl total volume for 30 minutes at 4°C and then washed three times with 1 ml lysis buffer. Proteins bound on the beads were eluted by boiling in SDS sample buffer and visualized by Coomassie staining of SDS-PAGE gels.

Generation of *Hnrnph2* mutant and knockout mice

For the R206W mutation, two conserved C nucleotides at positions 833 and 835 were substituted with T and G, respectively (c.833 C > T and c.835 C > G). For the P209L mutation, the C nucleotide at position 842 was substituted with T (c.842 C > T). gRNA was in vitro transcribed using MEGAshortscript T7 kit (Life Tech Corp; AM1354), and the template PCR amplified using the following primers:

Forward: 5′ - CCTTAATACGACTCACTATAGGGCTCATGACTATGCAGCGCCGTTTTAGAGCTAGAAATAGC-3′

Reverse: 5′- AAAAGCACCGACTCGGTGCCACTTTTTCAAGTTGATAACGGACTAGCCTTATTTTAACTTGCTATTTCT AGCTCTAAAAC-3′

The resulting PCR products contained the T7 promoter, gRNA sequence, and tracrRNA (5′-CCTTAATACGACTCACTATAGGGCTCATGACTATGCAGCGCCGTTTTAGAGCTAGAAATAGCAAGTTAAAATAAGGCTAGTCCGTTATCAACTTGAAAAAGTGGCACCGAGTCGGTGCTTTT-3′). Synthetic single-strand DNA was used as mutation donor. Donor DNA sequences are shown below.

P209L: 5′- ACAAGGAAAGAATAGGGCATAGGTACATCGAAATCTTCAAGAGTAGCCGAGCTGAAGTCCGAACCCACTATGATCCACCTAGAAAGCTCATGACTATGCAGCGCCCGGGTCTTTACGATAGGCCAGGGGCTGGAAGAGGGTATAATAGCATTGGCAGAGGAGCCGGGTTTGAAAGAATGAGGCGGGGTGCCTATGGTGGA-3′

R206W: 5′- AACACAAGGAAAGAATAGGGCATAGGTACATCGAAATCTTCAAGAGTAGCCGAGCTGAAGTCCGAACCCACTATGATCCACCTAGAAAGCTCATGACTATGCAGTGGCCGGGTCCTTACGATAGGCCAGGGGCTGGAAGAGGGTATAATAGCATTGGCAGAGGAGCCGGGTTTGAAAGAAT

GAGGCGGGGTGCCTATGGT-3′

The gRNAs, Cas9 mRNA, and ssDNA were co-microinjected into C57BL/6J zygotes at 25, 25, and 10 ng/µl respectively. Seven mice with P209L and 11 mice with R206W mutations were identified by PCR (5′-GACACTGCCAGTGGACTTTC-3′ and 5′-TGCTCTGGAAACTGGACCCA-3′) followed by sequencing (5′-TGCTCTGGAAACTGGACCCA-3′). These potential founders were crossed with WT C57BL/6J mice to confirm transmission of the mutation. Resulting progeny carrying the mutations were tested for possible off-target effects as predicted by the Wellcome Sanger Institute Genome Editing Off-Target by Sequence tool (8). Of the 61 predicted off-target sites (1: 0, 2: 0, 3: 9, 4: 52), all nine 3-nucleotide mismatch sites were tested by high-resolution melt analysis. All but one of the lines tested showed no off-target effects at these sites (**Supplemental Figure 4**). One line gave variant calls on all 9 sites, which was attributed to low DNA concentration of the sample. Nevertheless, this line was discarded. One line of each mutation (P209L, R206W, and KO) was chosen for phenotyping and heterozygous mutant or KO females bred to C57BL/6J males to maintain the genetic background. Subsequent generations were genotyped by Transnetyx automated real-time PCR (Transnetyx).

Breeding of experimental cohorts

For most experiments, heterozygous mutant females were bred to WT males to generate heterozygous mutant or KO females ($Hnrnph2^{R206W/X}$, $Hnrnph2^{P209L/X}$, $Hnrnph2^{KO/X}$), hemizygous mutant or KO males ($Hnrnph2^{R206W/Y}$, $Hnrnph2^{P209L/Y}$, $Hnrnph2^{KO/Y}$), WT females ($Hnrnph2^{X/X}$), and WT males ($Hnrnph2^{X/Y}$). In addition, for some experiments heterozygous mutant females were bred to hemizygous mutant or KO males to generate homozygous mutant or KO females ($Hnrnph2^{R206W/R206W}$, $Hnrnph2^{KO/KO}$), heterozygous mutant or KO females ($Hnrnph2^{R206W/X}$, $Hnrnph2^{KO/X}$), hemizygous mutant or KO males ($Hnrnph2^{R206W/Y}$, $Hnrnph2^{KO/Y}$), and WT males $Hnrnph2^{X/Y}$. We note that this cross could not be performed in the $Hnrnph2^{\mathrm{P209L}}$ line, as very few $Hnrnph2^{P209L/Y}$ males survived until sexual maturity (6-8 weeks). All experiments were performed

on generation F3 or later. Animals were group housed under standard conditions.

Mendelian inheritance and survival up to 8 weeks

All pups born and genotyped (samples collected from live pups at P2-P7 and from pups found dead before P2-P7 sample collection) in the colonies from April 2018 to March 2021 were included in calculation of genotype ratios. All pups born and genotyped during this time were also included in survival analyses, except for mice used in cohort 2 (audiogenic seizure cohort) and cohort 3 (µCT and imaging cohort).

Behavioral phenotyping and long-term survival

Experimental cohort 1, consisting of male hemizygous mutants or KOs, female heterozygous mutants or KOs, and WT littermate controls, were first subjected to an observational test battery at 8 weeks old. This was followed by more specific and sensitive tests of motor and sensory function at 8-9 weeks and 10-12 weeks, respectively. These mice were also weighed weekly from 3 to 8 weeks, then again at 6 months and every 6 months thereafter and followed for survival.

A slightly modified protocol of the EMPReSS (European Mouse Phenotyping Resource for Standardized Screens) version of SHIRPA (SmithKline Beecham, Harwell, Imperial College, Royal London Hospital, Phenotype Assessment) level 1 observational test battery was used (9). Briefly, mice were observed undisturbed in a clear viewing jar for activity, tremor, palpebral closure, coat appearance, skin color, whisker appearance, lacrimation, defecation, and urination. Mice were then moved to an arena and the following parameters scored: transfer arousal, locomotor activity, gait, pelvic elevation, tail elevation, startle response, touch escape and righting reflex. Thereafter, mice were held by the tail and scored for positional passivity, trunk curl, limb clasping, and visual placing. After placement on a wire mesh grid, mice were assessed for corneal reflex, pinna reflex, whisker orienting reflex, toe pinch response, and

negative geotaxis. Lastly, contact righting response when place in a tube and rolled upside down was tested, and any evidence of biting and excessive vocalization noted. The data were quantified using a binary scoring system as previously described (10). A normal behavior received a score of 0 and an abnormal behavior received a score of 1, enabling a global abnormality score to be determined for each mouse, with a higher score corresponding to a greater degree of abnormality. In addition, scores were also generated for specific functions including motor, sensory, neuropsychiatric, and autonomic function (11).

Rotarod analysis was performed on an accelerating rotarod apparatus (IITC Life Science) using a 2-day protocol. Mice were trained on the first day with one session set at 4 rpm for 5 minutes. The following day, rotation speed was set to accelerate from 4 to 40 rpm at 0.1 rpm/s, mice were placed on the apparatus, and the latency to fall was recorded for four separate trials per mouse. Mice were given a 15-minute rest period between each trial. Grip strength was measured using a grip strength meter (Bioseb) as grams of force for all 4 paws for each mouse in six repeated measurements. The beam walking test was performed using a 2-day, multi-beam protocol (12). Briefly, on day 1 mice were trained to walk across an elevated 12-mm square beam to reach an enclosed goal box. On day 2, mice received one trial each on a 12-mm square beam, a 6-mm square beam, and a 12-mm round beam, and latency to cross, number of hind paw slips, and number of falls recorded. A custom neurological scoring system was also used, where a score of 0 was given if the mouse was unable to traverse the beam in 60 s, 1 if a mouse traversed the entire beam by dragging itself with its front paws (hind paws remain in contact with the side of the beam at all times), 2 if a mouse was able to traverse the beam with some hind paw stepping on top of the beam before starting to drag itself with its front paws, 3 if a mouse was able to traverse the entire beam with hind paw stepping, but placed its hind paws on the side of the beam at least once (no dragging with front paws), and 4 if a mouse was able to traverse the entire beam with hind paw stepping and never placing its hind paws on the side of the beam. In the wire hang test, mice were placed onto a wire cage top, which was

then inverted and elevated above a clean cage, and latency to fall (up to 120 s) recorded. For gait analysis, the front and hind paws of each animal were dipped in red and blue paint (water-soluble and non-toxic), respectively. The animal was then placed in a 70-cm long tunnel lined on the bottom with Whatman filter paper, the entrance sealed, and animal allowed to walk through one time. Footprints were scanned and analyzed with Image J for stride length, fore- and hind base width, and overlap (13).

Experimental cohort 2, consisting of male hemizygous mutants or KOs, female heterozygous mutants or KOs, female homozygous mutants or KOs, and WT littermate controls, were tested for audiogenic seizure susceptibility in a clear acrylic box (30 x 30 x 30 cm), with a 6" red fire bell mounted to the underside of a removable lid, and connected to a standard GraLab timer. The bell consistently produced 120-125 dB sound as measured from inside the closed box using a digital sound level meter. At P21, mice were removed from their home cage one by one just before testing, put into a clean holding cage, and moved to the testing room. Mice were then transferred to the audiogenic seizure chamber and allowed to explore the box for 15 s before the bell was turned on for 60 s. The intensity of the response (seizure severity score) was categorized as 0 for no response or slight startle, 1 for wild running, 2 for clonic seizures, 3 for tonic seizures, and 4 for respiratory arrest (14).

Experimental cohort 4, consisting of hemizygous R206W males and WT littermate controls, were subjected to a battery of tests over 4 weeks to assess learning and memory, emotional behaviors, and social behaviors. To reduce any potential carryover effects, tests were run in the order of least invasive to most invasive, based on the sensitivity of tests on previous handling, and stress induced by each test (15). Tests were conducted with a 1-2 day interval, which has been shown to have little impact on performance compared to 1 week inter-test intervals (16). Starting at 8 weeks old, experimentally naïve mice were tested for anxiety in the elevated plus maze, anxiety and locomotion in the open field test, visual recognition memory in the novel object recognition test, spatial working memory in the Y maze spontaneous alternation

test, social preference in the three-chamber social interaction test, spatial learning and memory in the Morris water maze, and repetitive, compulsive-like behavior in the marble burying test. For all tests, mice were moved to the test room, kept behind a room divider, and allowed to acclimatize undisturbed for 30 minutes before starting tests. At the end of a test, mice were placed in a holding cage until all mice from a home cage completed testing, before being returned to the home cage. During all tests, the investigator remained out of sight behind a room divider. Behavioral parameters were recorded and analyzed using ANY-maze automated activity monitoring system and software (v7.2). To facilitate automated tracking and reduce stress on the animals, all tests were conducted under low (30-50 Lux), indirect lighting. Mazes, objects (novel object recognition test) and wire cages (three-chamber social interaction test) were thoroughly cleaned with 70% vol/vol ethanol after each test and allowed to dry before starting the next test.

In the elevated plus maze (Stoelting), mice were placed in the center facing an open arm away from the investigator and allowed to explore the maze undisturbed for 5 minutes (17). Parameters including total distance traveled, mean speed, open and closed arm entries and time, and center entries and time were recorded and analyzed for the total test time, as well as temporally across the session to assess any potential differences in habituation to novelty and aversive learning (18, 19). The animal's entire area was used to score arm entries and exits, with at least 80% of the animal needed for an arm entry to occur, and mice were considered to be in the center zone if not in any arm. Mice that fell off the maze were excluded from the analysis.

The open field test was run according to the protocol by Seibenhener and Wooten (20). Briefly, mice were placed in the center of a 40 cm x 40 cm open field arena (Stoelting) facing away from the investigator and allowed to explore undisturbed for 20 minutes. Parameters including total distance traveled, mean speed, time in the center zone (defined as the center 24 cm x 24 cm of the maze) were recorded and analyzed for the total test time, as well as

temporally across the session to assess any potential differences in habituation to novelty (21). The animal's center was used to score zone entries and exits.

The novel object test protocol used was based on those published by Leger et al. (22) and Lueptow (23) and included a familiarization phase and test phase. As the test was conducted in the same open field arena that was used in the open field test a few days prior, a habituation phase was not included. During the familiarization phase, mice were placed in the center of a 40 cm x 40 cm open field arena (Stoelting) containing 2 identical novel objects (either white wooden balls with flat bottoms, or grey wooden squares; Stoelting) and allowed to explore undisturbed for 10 minutes. For the testing stage (24 hours after familiarization), one of the objects was switched out for a novel object. The pair of objects used and the position of the novel object (top left vs bottom right) was randomized between mice and groups. Data analysis was limited to the time needed to reach 20 seconds of total object exploration time. Mice that failed to reach 20 seconds of total object exploration time were excluded from the analysis. Parameters including total distance traveled, mean speed, time taken to reach 20 seconds of total exploration time, and time exploring each object were recorded and analyzed. Object exploration was defined as the animal's head being within 20 mm of an object and oriented toward the object (orientation angle of 60°). Instances where mice climbed on top of the objects (animal's center is inside the object) were not considered to be object exploration. An object discrimination index was calculated for the familiarization stage (top left vs bottom right object) and the test stage (novel vs known object) by subtracting the time exploring the bottom right/known object from the time exploring the top left/novel object, divided by the total object exploration time (20 seconds).

In the Y maze (Stoelting), mice were placed in a distal part of an arm, facing away from the investigator and allowed to explore the maze freely for 8 minutes. Parameters including total distance traveled, mean speed, number of entries and time spent in each arm and the maze center were recorded and analyzed for the total test time. The animal's entire area was used to

score arm entries and exits, with at least 80% of the animal needed for an arm entry to occur, and mice were considered to be in the center zone if not in any arm. A spontaneous alternation was defined as consecutive entry into 3 different arms on overlapping triplet sets, and the percentage of spontaneous alternations was calculated as the number of spontaneous alternations divided by the total number of arm entries minus 2, multiplied by 100 (24). Mice that climbed out of the maze were excluded from the analysis.

The three-chamber social interaction test consisted of a pre-test, followed the next day by a social preference test and social novelty test, with an inter-test interval of 5 minutes (25). In the pre-test, mice were placed in the center chamber of the sociability cage (Stoelting) facing away from the investigator and allowed 10 minutes to freely explore the cage, which contained crumpled paper balls inside wire enclosures placed in the right and left chambers of the cage. The next day, the 2 wire cages contained either a wooden block to serve as the non-social stimulus, or an unfamiliar age-, sex-, and background strain-matched WT mouse to serve as the social stimulus. The test mouse was placed in the center chamber of the cage facing away from the investigator and allowed to explore the cage undisturbed for 10 minutes. At the end of the test, the test mouse was removed and placed into a holding cage and the wooden block was replaced by an unfamiliar age-, sex-, and background strain-matched WT mouse to serve as the novel social stimulus. The mouse used as the social stimulus during the social preference test was kept in the wire cage and served as the known social stimulus during the social novelty test. After 5 minutes, the test mouse was again placed in the center chamber of the sociability cage facing away from the investigator and allowed to explore the cage undisturbed for 10 minutes. Parameters including total distance traveled, mean speed, and time investigating each stimulus were recorded and analyzed. Stimulus investigation was defined as the animal's head being within 25 mm of the wire cage and oriented toward the stimulus (orientation angle of 60°). A social preference index was calculated by subtracting the time spent investigating the non-social stimulus from the time spent investigating the social stimulus and dividing the result by

the time spent investigating the social stimulus plus the non-social stimulus. A social novelty index was calculated by subtracting the time spent investigating the known social stimulus from the time spent investigating the novel social stimulus and dividing the result by the time spent investigating the known social stimulus plus the novel social stimulus (25).

The Morris water maze protocol was modified from that of Vorhees and Williams (26). A blue circular plastic pool was used with a diameter of 120 cm and depth of 81 cm along with an adjustable height circular platform (grey for cued trials, clear for training trials) with a diameter of 10 cm (MazeEngineers). The pool was filled with water to approximately 20 cm from the top and allowed to equilibrate to room temperature (approximately 20°C) for at least 2 days. To facilitate automated tracking and reduce visibility of the platform during training trials, 3 bottles of non-toxic white tempera paint were added to the pool. A cued test was first performed during which the pool was surrounded by black room dividers to eliminate any distal room cues and the platform was visible (height was adjusted to just above the surface of the water and a red plastic flag was attached to the grey platform to improve visibility). The cued test consisted of 4 trials performed on a single day, with an inter-trial interval of 10-15 minutes (mice were run in blocks of 10 and all mice completed a trial before the next trial was started). The position of the visible platform was moved for each trial (southeast, northeast, southwest, northwest) and the starting position was alternated between north and west. Mice were gently placed in the water facing the pool wall and given 60 seconds to find and climb on to the visible platform. Mice that failed to find the platform were gently picked up and placed on the platform. Once on the platform, they were allowed to remain there for 15 seconds. At the end of each trial, mice were returned to a heated holding cage while waiting to start the next trial and returned to the home cage at the completion of the 4 trials. Two days later, the mice were subjected to 4 days of training, consisting of 4 trials per day, with an inter-trial interval of 10-15 minutes (mice were run in blocks of 10 and all mice completed a trial before the next trial was started). During training trials, only 1 black room divider was used to hide the investigator from view during the test. A

black and white stripe poster on the room divider, as well as objects in the testing room around the pool (e.g., two lamps, wall cabinet) served as distal spatial cues. A clear platform was submerged 1-2 cm below the surface of the water and was not visible under the water with white paint added. A set of semi-randomly selected distal start positions were used, with the platform remaining in the southwest quadrant as previously described (26). Mice were gently placed in the water facing the pool wall and given 60 seconds to find and climb on to the hidden platform. Mice that failed to find the platform were gently picked up and placed on the platform. Once on the platform, they were allowed to remain there for 15 seconds. At the end of each trial, mice were returned to a heated holding cage while waiting to start the next trial and returned to the home cage at the completion of the 4 trials. After 4 days of training, mice were tested for spatial memory in a single probe trial, during which the platform was removed from the pool. Mice were gently placed in the water facing the pool wall at a location not used during training, directly across from the previous location of the platform (northeast), and removed after 30 seconds. Parameters including total distance traveled, mean swim speed, time in each quadrant, latency to reach the platform (cued and training trials), percentage of time in the thigmotaxis zone (within 10 cm of the pool wall), cumulative distance from hidden platform (training trials), platform location crossings (probe trial), and mean distance from platform location (probe trial) were recorded and analyzed. Data for the training trials are averaged across 4 trials per day and plotted as block means. The animal's center was used to score zone entries and exits.

The marble burying test was performed according to the protocol by Angoa-Perez et al. (27). Standard polycarbonate rat cages were filled with fresh mouse bedding to a depth of 5 cm and the bedding surface leveled. Twenty standard glass toy marbles of assorted styles and colors were placed gently on the surface of the bedding in 5 rows of 4 marbles each. A single mouse was placed in a cage away from the marbles, the cage was covered with a filter-top lid, and the mouse allowed to remain in the cage undisturbed for 1 hour. After the test, the mouse

was removed, taking care not to move any marbles, and returned to its home cage. The number of marbles buried (at least two-thirds of surface area covered by bedding) was counted.

EEG implantation

EEG/EMG headstage (Model 8431-SM, Pinnacle Technology) implantation was completed according to the manufacturer's instructions unless otherwise noted. In brief, mice were anesthetized using isoflurane vapors at 3% and maintained during implantation at 1.5%. A small midline incision was made, exposing the skull. Six holes were made in the skull using a 23-gauge needle; following this, each hole had a screw with a wire lead attached placed inside. Dental cement (DuraLay, Reliance Dental Manufacturing) was used to cover the screws, leaving the wires exposed. The headstage was then placed on top of the dried dental cement and EMG leads were inserted into the nuchal muscles via a pocket created using forceps. Headstage wires were then connected to the lead wire from the screw using wire glue (Anders Products) and allowed to dry completely before covering them with dental cement. Animals were then given a post-operative analgesic injection (meloxicam, 2 mg/kg) and mush food for the next three days to ease recovery. Animals were given at least one week to recover prior to data collection.

EEG data collection and analysis

EEG/EMG collection was synchronized with video recording using Pinnacle Seizure Acquisition Software (v2.1.0). Data was obtained using the Pinnacle Acquisition System with a 8406-SE31M pre-amplifier at a sampling rate of 2000 Hz with a high and low bandpass filter at 0 and 500 Hz, respectively. Representative 1-hour time spans were selected in an unbiased manner (12am-1am and 6:30am-7:30am for the dark and light phases, respectively) and were analyzed blind to genotype for spectral power and epileptiform activity. All analyzed data was recorded after at least 2 hours of acclimation to the recording arena and connection to the wiring tether.

Electrical activity was converted to frequency using a fast Fourier transformation (FFT) algorithm using the Hann method. Pinnacle Sirenia Seizure Pro (v2.2.5) software was used for spectral power analysis at individual frequencies and delta (0.5-4 Hz), theta (5- Hz), alpha (9-13 Hz), and beta (14-30 Hz) bands. Spectral power data were used in combination with video recordings to rule out motion-based artifacts and to manually quantify the percentage of time spent in epileptiform activity. Spikes that were greater than 2x the baseline amplitude and <200 ms in duration and showing polyspikes (defined as spikes crossing baseline more than two times) were considered epileptiform. The "time spent" satisfying criterion was summed across the entire hour and calculated as the percentage of time spent exhibiting the phenotype. The lambdoid lead EEG waveform and video from 12am to 1am was watched and scored to detect any absence-like (behavioral arrest during EEG spiking) or motor (behavioral changes similar to myoclonic jerks, tonic-clonic action, wild running, or postural loss) seizures.

In vivo MRI and μCT

Experimental cohort 3, consisting of male hemizygous mutants or KOs, female heterozygous mutants or KOs, and WT littermate controls, were imaged at the Center for In Vivo Imaging and Therapeutics at St. Jude Children's Research Hospital using micro-computed tomography (μCT) and magnetic resonance imaging (MRI) at 6 and 24 weeks of age. The μCT was performed on a Siemens Inveon PET/CT system (Siemens) at 88-μm resolution, and the MRI was performed on a Bruker Clinscan 7T MRI system (Bruker Biospin MRI GmbH). MRI was acquired with a mouse brain surface receive coil positioned over the mouse head and placed inside a 72-mm transmit/receive coil. After the localizer, a T2-weighted turbo spin echo sequence with variable flip-angle echo trains was performed in the coronal orientation (TR/TE = 2500/114 ms, matrix size = 192 × 192 x 104, resolution = 0.12 x 0.12 x 0.12 mm, number of averages = 4). Prior to scanning, mice were anesthetized in a chamber (3% isoflurane in oxygen delivered at 1 L/min) and maintained using nose-cone delivery (1-2% isoflurane in oxygen delivered at 1 L/min).

Animals were provided thermal support using an inbuilt electronic heating pad (μCT) or a heated bed with warm water circulation (MRI) and a physiological monitoring system to monitor breath rate. After imaging, animals were allowed to recover on a heating pad.

Morphometric analysis was performed on the μCT images to identify group differences in skull shape. Linear measurements of 11 key craniofacial parameters (28) were performed manually on μCT slices using Inveon Research Workplace software (IRW 4.2, Siemens). This was followed by automated imaged-based shape analysis using a population-level atlas of the *Mus musculus* craniofacial skeleton (29). Briefly, the head was extracted from the whole-body μCT images using an iterative search and best-match algorithm. The μCT atlas (https://github.com/muratmaga/mouse_CT_atlas) was then aligned to native space images using a first pass affine transform, followed by a non-linear warping. The calculated transform was then applied to a set of 51 previously identified landmarks and the coordinates for the landmarks in native space were extracted. Processing steps were performed using the ANTS software package (https://github.com/ANTsX/ANTsPy). All alignment results were visually inspected by at least 2 raters. The Euclidean distance between each point was calculated and used for subsequent analysis. First, we performed pairwise comparisons of linear distances between all 51 landmarks. Next, we performed Euclidean distance matrix analysis (EDMA), a geometric morphometric approach enabling the quantification and comparison of shape in three dimensions (30). For global EDMA analysis all 51 landmarks were included, whereas the regional EDMA analysis was performed on a subset of landmarks that summarize regions with specific embryonic tissue origins, further divided into anatomically relevant subsets including palate, midface, and nasal regions (31). To account for overall difference in size, both the global and regional EDMA analyses were scaled to centroid size (calculated as the square root of the sum of squared distances of all landmarks from their centroid), which is a common proxy for overall size in geometric morphometric analyses (32).

Brain parcellation and volumetrics were performed to investigate group differences in

total and regional brain volumes. We used the DSURQE atlas (33), which contains 336 cortical, white matter, subcortical, and CSF defined regions. The DSURQE anatomical image was first downsampled to 120-μm isotropic resolution to satisfy the Nyquist criteria of our image resolution and reduce computational time for fitting. The acquired T2 images were preprocessed, including skull-stripping and intensity normalization. The images were then aligned to the atlas by a first-pass affine registration, followed by a non-linear warping. The inverse warping was applied to the labeled atlas to bring all labeled areas into native space. All image processing steps were performed using the AFNI software package (https://afni.nimh.nih.gov/). The volume (number of voxels times native resolution) of each labeled area from the atlas was extracted for subsequent analysis. The results of the inverse warping were quality checked by visual inspection by at least 2 raters. Cases with poor alignment (17 out of a total of 140) were removed from the final volumetric analysis.

Mouse histology and immunofluorescence

For confirmation of hydrocephalus, mice were anesthetized by isoflurane inhalation and transcardially perfused with 10% neutral buffered formalin (NBF) (mice flagged for domed heads) or postfixed in 10% neutral buffered formalin (mice found dead). Heads were decalcified, paraffin-embedded in the coronal plane, 10 4-μm step sections (every 50 μm) cut, stained with hematoxylin and eosin (H&E), and reviewed by a veterinary pathologist.

Brains from experimentally naïve male hemizygous mutants or KOs and male WT littermate controls were harvested at 8 weeks (*Hnrnph2*^{R206W} and KO) or 3 weeks (*Hnrnph2*^{P209L}) of age for histology and immunofluorescence. Briefly, mice were anesthetized by isoflurane inhalation and transcardially perfused with 10% NBF, the brain dissected from the skull and cut in half on the sagittal plane, processed for paraffin embedding, and cut at 10 μm. Sections were stained with H&E and Luxol fast blue-cresyl violet (LFB-CV) to ascertain overall morphology and myelination. In addition, IHC was performed using antibodies against neurons (NeuN; 2367, Cell

Signaling Technology), astrocytes (GFAP; Z0334, DAKO), microglia (IBA1; CP290A, BioCare Medical), and oligodendrocytes (OLIG2; ab109186, Abcam). Sections were deparaffinized, followed by heat-induced epitope retrieval (HIER) with appropriate buffer (AR9640, Leica; 950-500 or 760-107, Roche), incubation with primary antibodies and Bond Polymer Refine Detection with DAB (DS9800, Leica), or incubation with OmniMap Rabbit HRP antibody (760-4311, Roche) and ChromoMap DAB (760-159, Roche). Lastly, sections were counterstained with hematoxylin and, if needed, post-counterstained with Bluing Reagent (760-2037, Roche), before being coverslipped. Sections were reviewed by a veterinary pathologist and immunoreactivity quantified using HALO image analysis platform (Indica Labs). The number of NeuN-positive cells was quantified using QuPath software (34). Briefly, visual, somatosensory, and somatomotor cortices were manually annotated according to the Allen mouse brain atlas, and QuPath's positive cell detection function applied. The number of NeuN-positive neurons were expressed as number of cells per mm^2.

To assess the expression of hnRNPH2, immunofluorescence was performed using an N-terminal hnRNPH2 antibody (ab179439, Abcam) or a C-terminal hnRNPH2 antibody (ab181171, Abcam), as well as antibodies for neuronal nuclei (NeuN; ab104224, Abcam), or neuronal cytoplasm and processes (beta III tubulin; ab78078, Abcam). To assess cortical cytoarchitecture, immunofluorescence was performed using antibodies against SATB2 (sc-81376, Santa Cruz Biotechnologies), which is broadly expressed in upper layer (II-IV) neurons as well as in subpopulations of deep layer (V-VI) neurons, CTIP2 (ab18465, Abcam), which is expressed exclusively in a subpopulation of layer V neurons, and FOXP2 (HPA000382, Atlas Antibodies), which is expressed in layer VI neurons. Sections were deparaffinized, followed by HIER using Universal Antigen Retrieval Reagent (Roche, CTS015), permeabilization in PBS containing 2% Triton X-100, and treatment with TrueBlack Lipofuscin Autofluorescence Quencher (23007, Biotium). Thereafter, slides were blocked in PBS containing 4% bovine serum albumin (A2153, Sigma-Aldrich) and 2% normal goat serum (S-1000, Vector

Laboratories), and incubated with primary antibodies and species-specific Alexa Fluor secondary antibodies (A32732, A11029, A21434, and A21244, Thermo Fisher Scientific). Finally, slides were coverslipped with anti-fade mounting media containing DAPI (P36931, Thermo Fisher Scientific). Fluorescence slide scanning was performed using a Zeiss Axio Scan.Z1 with a Hamamatsu ORCA-Flash4.0 V3 camera using Zeiss ZEN 3.1 software. Images were created with a Zeiss Plan-Apochromat 20X/0.8 objective lens with illumination by Zeiss Colibri.2 LEDs (365 nm, 470 nm, 555 nm) and corresponding filters (Zeiss Filter Set 49, 38 HE, and 43 HE, respectively). For subcellular localization of mutant hnRNPH2, fluorescent imaging was performed using a Yokogawa CSU W1 spinning disk attached to a Nikon Ti2 eclipse with a Photometrics Prime 95B camera using Nikon Elements software. A 60× Plan Apo 1.40NA oil objective was used and Perfect Focus 2.0 (Nikon) was engaged for all captures. Imaging was performed using 405-nm, 488-nm, and 561-nm lasers for DAPI, Alexa Fluor 488, and Alexa Fluor 555, respectively. Image J/Fiji software (35) was used for maximum intensity Z-projection and color image processing (LUT Fire) for visualization of cytoplasmic hnRNPH2 signal. For cortical cytoarchitecture, fluorescently labeled cells were quantified using QuPath software (34). Briefly, rectangular regions of interest were positioned over visual, somatosensory, and somatomotor cortical regions, with each region of interest subdivided into eight equal bins from the pia to the inner border of the cortex (36). QuPath's positive cell detection function was used to detect all cells using the DAPI channel, followed by application of a single measurement classifier for the remaining channels. The distribution of neurons was expressed as the number of SATB2, CTIP2, and FOXP2 neurons as a percentage of the total number of DAPI-positive cells within each bin.

Mouse in situ hybridization

Whole embryos and brains of WT C57BL/6J mice were harvested at embryonic day 12.5, 14.5, 16.5 and postnatal day 0, 7, 14, and 56, respectively. Samples were fixed in 10% neutral

buffered formalin and in situ hybridization performed with a chromogenic (Fast Red), single-plex BaseScope assay (Advanced Cell Diagnostics) according to the manufacturer's instructions with custom probes against *Hnrnph1* (BA-Mm-Hnrnph1-3zz-st) and *Hnrnph2* (BA-Mm-Hnrnph2-2zz-st). Slides were scanned on the PANNORAMIC 250 Flash digital scanner (3DHISTECH) and analyzed using HALO image analysis platform according to the RNAscope quantification protocol (Indica Labs). Briefly, cells in a tissue section were grouped into 5 bins based on the number of dots per cell ranging from 0+ to 4+. Clusters were divided by the typical probe signal area to calculate a dot number for the cluster in identified cells of interest. Each sample was evaluated for the percentage of cells in each bin. The H-score for the sample was calculated by totaling the percentage of cells in each bin according to the weighted formula shown below, and a single score was assigned to an entire tissue section based on the average target expression in this tissue. H-scores were provided on a weighted scale of 0–400. The H-score was calculated using the algorithm with the following equation: H-Score = $(1 \times \%\ Probe\ 1 + Cells\ ) + (2 \times \%\ Probe\ 2 + Cells\ ) + (3 \times \%\ Probe\ 3 + Cells\ ) + (4 \times \%\ Probe\ 4 + Cells\ )$.

Mouse Western blots and digital droplet RT-PCR

Brains from experimentally naïve male hemizygous mutants or KOs and male WT littermate controls were harvested at 8 weeks (*Hnrnph2*^{R206W} and KO) or 3 weeks (*Hnrnph2*^{P209L}) of age for Western blots and ddRT-PCR. In addition, brains of WT C57BL/6J mice were harvested at embryonic day 12.5, 14.5, 16.5 and postnatal day 0, 7, 14, and 56. Brains were removed, cortices dissected out, flash frozen in liquid nitrogen, and stored at -80°C.

For Western blots, samples were subjected to sequential solubility fractionation or nucleocytoplasmic fractionation as previously described (37). Protein concentrations were determined by DC protein assay (5000111, Bio-Rad), and 35 µg RIPA-soluble, 80 µg cytoplasmic protein, or maximum volume nuclear lysate (40 µl) was loaded onto the gel. Electrophoresis was performed using the Bolt Bis-Tris Plus mini gel system (Thermo Fisher

Scientific). Gels were transferred to PVDF membranes using the iBlot 2 dry blotting system (Thermo Fisher Scientific), blocked in Odyssey TBS blocking buffer (LI-COR), incubated with primary antibodies against hnRNPH2 (ab179439, Abcam), hnRNPH1 (PA5-50678, Thermo Fisher Scientific), GAPDH (97166, Cell Signaling Technology), or lamin A/C (Cell Signaling Technology, 2032), followed by species-specific IRDye secondary antibodies (925-3221, 925-68070, LI-COR). Blots were imaged on the Odyssey CLx system and analyzed on Image Studio software (LI-COR). The full, uncut gels are included in the Supplemental Material.

For ddRT-PCR, samples were treated with RNAlater-ICE (Thermo Fisher Scientific), RNA extracted using the RNeasy Plus Universal Mini Kit (73404, Qiagen), and treated for DNA contamination with the TURBO DNA-free kit (AM1907, Thermo Fisher Scientific). 10 ng RNA was used with a one-step RT-ddPCR advanced kit for probes (1864021, Bio-Rad), together with the following assays: Mouse *Gapdh* Primer Limited (Mm99999915_g1, Thermo Fisher Scientific), Mouse *Rpp30* (dMmuCPE5097025, Bio-Rad), Mouse *Hnrnph1* (Mm00517601_m1, Thermo Fisher Scientific), and Mouse *Hnrnph2* (Mm01340844_g1, Thermo Fisher Scientific).

Primary cortical neuron culture

Primary culture of cortical neurons was prepared from P1 mice as previously described (38). The cells were plated on 8-well dishes (Lab-Tek chambered cover glass, Nunc, Thermo Fisher Scientific) coated with 10 µg/ml poly-L-ornithine (Sigma Aldrich) at a density of 30 x 10^4 cells/well and cultured in NeuroCult neuronal plating medium (Stem Cell Technologies) supplemented with B-27 (Thermo Fisher Scientific) and GlutaMAX Supplement (Gibco, Thermo Fisher). On day 5, primary neurons were transitioned to BrainPhys Neuronal Medium (Stem Cell Technologies) and supplemented with B-27 by performing half-medium changes every 3-4 days.

Magnetofection of primary cortical neurons

On day 6, neurons were transfected using paramagnetic nanobeads (NeuroMag, OZ Biosciences). pVectOZ-GFP (0.4 µg) (OZ Biosciences) was incubated with 0.75 µl NeuroMag beads in 50 µl Opti-MEM (Thermo Fisher Scientific) for 15 minutes and then added dropwise to the cultures. Cells were incubated on top of a magnetic plate (OZ Biosciences) for 15 minutes.

Neuronal cell staining and imaging

On day 14, neurons were fixed with 4% paraformaldehyde (Electron Microscopy Services) for 20 minutes, permeabilized with 0.1% Triton-X for 10 minutes, and blocked with 5% normal goat serum in TBST for 1 hr. Neurons were then further incubated with primary antibody against MAP2 (Abcam, ab5392, 1:1000), washed 3 times with TBST, incubated with a host-specific Alexa Fluor 647 antibody (Thermo Fisher, A21449, 1:1000) for 2 hours at room temperature, and washed 3 times with TBST and 2 times with PBS. Imaging was performed on a Yokogawa CSU W1 spinning disk attached to a Nikon Ti2 eclipse with a Photometrics Prime 95B camera using Nikon Elements software (v5.21.02). All imaging was performed on a Nikon Plan Apo 60× 1.40 NA oil objective, with Immersol 518 F (Zeiss). After finding neurons positive for both MAP2 and GFP, a tilescan of 592.92 µm x 592.92 µm was taken centered around the soma, with a z-step size of 0.3 µm for a total z-stack size between 15 µm and 40 µm per neuron. Acquisition of the GFP channel was performed using a 488-nm laser at 50% power with 100-ms exposure. Stitching was performed automatically in Nikon Elements.

Image analysis of dendritic arborization and spines

Stitched images were imported into Imaris (Bitplane v9.9.1), and using the in-built Filaments module, dendrites and dendritic spines originating from the centered soma in each image were segmented. Any dendrite originating from the cell body was considered as a primary dendrite. A dendritic branch point was determined when the original dendrite bifurcated into two or more daughter trees. For assigning branch level, the lowest level was given to the primary dendrites

and levels were increased when a branch point was reached. Dendritic full branch depth was calculated by the sum of dendritic full branch points of a given neuron and dendritic full branch level was the maximum value reached by any primary tree on a given neuron. For Sholl analyses, Sholl radii originating from the centroid of the soma were increased at 1-μm intervals. The Sholl intersection profile was obtained by counting the number of dendritic branches at each given distance from the soma and/or averaging them across the entire neuron. Morphometric measurements for dendrite full branch depth, dendrite full branch level, dendrite length, number of Sholl intersections, total number of spines, and spine density (per 10 μm) of the segmentation were extracted and plotted in GraphPad Prism 9.

RNA sequencing

Total RNA was extracted with an RNeasy Universal Plus Mini kit (Qiagen; 73404). A sequencing library was prepared with a TruSeq Stranded Total RNA Kit (Illumina) and sequenced with an Illumina HiSeq system with 100-bp read length. Total stranded RNA sequencing data were processed by the internal AutoMapper pipeline. Raw reads were first trimmed (Trim-Galore v0.60), mapped to mouse (GRCm38) or human (hg38) genome assembly (STAR v2.7) and then the gene level values were quantified (RSEM v1.31) based on GENCODE annotation (vM22). We obtained the TPM (transcript per million) counts for genes with TPM greater than one in at least one sample and the gene expression analysis was performed with non-parametric ANOVA using Kruskal-Wallis and Dunn's tests on log-transformed TPM counts between three replicates of each experimental group, implemented in Partek Genomics Suite v7.0 software (Partek). The expression of a gene was considered significantly different if $P < 0.05$ and $\log_2$FC > 0.5 in at least one of the group comparisons. The calculated z-scores of significantly differential expressed genes or $\log_2$Rs were plotted using hierarchical clustering in a heat map, using correlation distance measure, implemented in Spotfire v7.5.0 software (TIBCO). For alternative splicing analysis, the aligned and sorted BAM files after STAR alignment were used for AS

analysis using rMATS (v4.0.2) (50). A3SS, A5SS, SE, RI, and MXE events were evaluated. Significant AS events were identified while average coverage >10 and delta percent spliced in (ΔPSI) > 0.1.

Statistics

Significant differences from expected Mendelian inheritance ratios were determined by chi-square tests. The log-rank (Mantel-Cox) test was used to determine significant differences between survival curves and hazard ratios computed by a log-rank approach. Differences in body weight over time were determined by fitting a mixed-effects model (REML) for time, genotype, and time x genotype interaction, followed by Sidak's multiple comparisons test to compare WT mice to mutants or KOs. For differences in linear craniofacial measurements, we used a two-way ANOVA (line, genotype, line x genotype interaction) followed by Sidak's multiple comparisons test to compare WT mice to mutants or KOs. For MRI analysis and linear interlandmark distance analysis, Wilcoxon rank sum test was used to compare groups. In the EDMA analysis, biological shapes were compared using an EDMA bootstrap test (30). The global test is based on the pairwise distances in the form matrices, taking the max/min ratio of the distances. This is then done for all the B replicates, which provides the null distribution. The analysis was performed using the R package EDMAinR. Correction for multiple testing was performed using the FDR method. Significance for the incidence of hydrocephalus was determined by Fisher's exact test. SHIRPA and audiogenic seizure scores were analyzed by aligned ranks transformation (ART) non-parametric two-way ANOVA (line, genotype, line x genotype interaction), followed by Mann-Whitney U test to compare WT mice to mutants or KOs. Differences in all motor tests, optomotor response, and hot plate test were determined by two-way ANOVA (line, genotype, line x genotype interaction) followed by Sidak's multiple comparisons test to compare WT mice to mutants or KOs. Scent habituation data were analyzed by repeated measures two-way ANOVA (trial, genotype, trial x genotype interaction),

followed by Sidak's multiple comparisons test to compare WT mice to mutants or KOs. For the open field test, differences between mutants and WT controls were determined by unpaired t test when analyzing the total duration of the test, and by repeated measures two-way ANOVA (time bin, genotype, time bin x genotype interaction) followed by Sidak's multiple comparisons test when analyzing different time bins across the test. Significance for the incidence of falls from the elevated plus maze was determined by Fisher's exact test. Total distance was analyzed by unpaired t test, and percentage time spent in the maze zones was subjected to two-way ANOVA (zone, genotype, zone x genotype interaction), followed by Sidak's multiple comparisons test to compare mutants and controls. For time bin analysis across the elevated plus maze test, repeated measures two-way ANOVAs (time bin, genotype, time bin x genotype interaction), followed by Dunnett's multiple comparison test to time bin 1 (% time in open arms, % time in closed arms) or by Sidak's multiple comparisons test to compare mutants and controls (% time in center zone) was used. Morris water maze cued and training data were analyzed by repeated measures two-way ANOVA (trial/day, genotype, trial/day x genotype interaction), followed by Sidak's multiple comparisons test to compare mutants and controls. Probe trial data were analyzed by unpaired t test (mean speed, mean distance from platform location, % time in the thigmotaxis zone, platform location crossings) and two-way ANOVA (quadrant, genotype, quadrant x genotype interaction) followed by Sidak's multiple comparisons test to compare mutants and controls. Y maze, novel object recognition and three chamber social interaction data were analyzed by unpaired t tests. In the Y maze, the correlation between total arm entries and % spontaneous alternations were evaluated by Pearson's correlation. Nuclear hnRNPH2 levels in mouse cortex by Western blot were compared using unpaired t tests. *Hnrnph1* and *Hnrnph2* transcript levels measured by ddRT-PCR were analyzed by two-way ANOVA (line, genotype, line x genotype interaction) followed by Sidak's multiple comparisons test to compare WT to mutants or KOs. *Hnrnph1* and *Hnrnph2* expression by ddRT-PCR and ISH were analyzed by two-way ANOVA (genotype, developmental time point,

genotype x developmental time point interaction) followed by Sidak's multiple comparisons test to compare *Hnrnph1* levels to *Hnrnph2* levels at each time point, or Tukey's multiple comparisons test to compare transcript levels between developmental time points for each gene separately. *Hnrnph2* expression by ddRT-PCR in *Hnrnph2* KO mice were analyzed by one-way ANOVA followed by Sidak's multiple comparisons test to compare WT males to hemizygous KO males, as well as WT females to heterozygous and homozygous KO females. NeuN-positive cell counts were analyzed by two-way ANOVA (line, genotype, line x genotype interaction) followed by Sidak's multiple comparisons test to compare WT to mutants or KOs. For cortical layer analysis, the percentage of SATB2-, CTIP2- and FOXP2-positive neurons were analyzed by two-way ANOVA (genotype, bin, genotype x bin interaction) followed by Tukey's multiple comparisons test. A *P* value less than 0.05 was considered significant.

Study approval

All studies were approved by the St. Jude Children's Research Hospital institutional review committee on animal safety.

References

1. Kang HJ, Kawasawa YI, Cheng F, Zhu Y, Xu X, Li M, et al. Spatio-temporal transcriptome of the human brain. *Nature.* 2011;478(7370):483-9.
2. Pletikos M, Sousa AM, Sedmak G, Meyer KA, Zhu Y, Cheng F, et al. Temporal specification and bilaterality of human neocortical topographic gene expression. *Neuron.* 2014;81(2):321-32.
3. Johnson MB, Kawasawa YI, Mason CE, Krsnik Z, Coppola G, Bogdanovic D, et al. Functional and evolutionary insights into human brain development through global transcriptome analysis. *Neuron.* 2009;62(4):494-509.
4. Gordon A, Yoon SJ, Tran SS, Makinson CD, Park JY, Andersen J, et al. Long-term maturation of human cortical organoids matches key early postnatal transitions. *Nat Neurosci.* 2021;24(3):331-42.
5. Narina S, Connelly JP, and Pruett-Miller SM. High-Throughput Analysis of CRISPR-Cas9 Editing Outcomes in Cell and Animal Models Using CRIS.py. *Methods Mol Biol.* 2023;2631:155-82.
6. Connelly JP, and Pruett-Miller SM. CRIS.py: A Versatile and High-throughput Analysis Program for CRISPR-based Genome Editing. *Sci Rep.* 2019;9(1):4194.
7. Wang C, Ward ME, Chen R, Liu K, Tracy TE, Chen X, et al. Scalable Production of iPSC-Derived Human Neurons to Identify Tau-Lowering Compounds by High-Content Screening. *Stem Cell Reports.* 2017;9(4):1221-33.
8. Hodgkins A, Farne A, Perera S, Grego T, Parry-Smith DJ, Skarnes WC, et al. WGE: a CRISPR database for genome engineering. *Bioinformatics.* 2015;31(18):3078-80.
9. Gates H, Mallon AM, Brown SD, and Consortium E. High-throughput mouse phenotyping. *Methods.* 2011;53(4):394-404.
10. Glynn D, Bortnick RA, and Morton AJ. Complexin II is essential for normal neurological function in mice. *Hum Mol Genet.* 2003;12(19):2431-48.
11. Rogers DC, Fisher EM, Brown SD, Peters J, Hunter AJ, and Martin JE. Behavioral and functional analysis of mouse phenotype: SHIRPA, a proposed protocol for comprehensive phenotype assessment. *Mamm Genome.* 1997;8(10):711-3.
12. Luong TN, Carlisle HJ, Southwell A, and Patterson PH. Assessment of motor balance and coordination in mice using the balance beam. *J Vis Exp.* 2011(49).
13. Brooks SP, Trueman RC, and Dunnett SB. Assessment of Motor Coordination and Balance in Mice Using the Rotarod, Elevated Bridge, and Footprint Tests. *Curr Protoc Mouse Biol.* 2012;2(1):37-53.
14. Musumeci SA, Calabrese G, Bonaccorso CM, D'Antoni S, Brouwer JR, Bakker CE, et al. Audiogenic seizure susceptibility is reduced in fragile X knockout mice after introduction of FMR1 transgenes. *Exp Neurol.* 2007;203(1):233-40.
15. McIlwain KL, Merriweather MY, Yuva-Paylor LA, and Paylor R. The use of behavioral test batteries: effects of training history. *Physiol Behav.* 2001;73(5):705-17.
16. Paylor R, Spencer CM, Yuva-Paylor LA, and Pieke-Dahl S. The use of behavioral test batteries, II: effect of test interval. *Physiol Behav.* 2006;87(1):95-102.
17. Walf AA, and Frye CA. The use of the elevated plus maze as an assay of anxiety-related behavior in rodents. *Nat Protoc.* 2007;2(2):322-8.
18. Carobrez AP, and Bertoglio LJ. Ethological and temporal analyses of anxiety-like behavior: the elevated plus-maze model 20 years on. *Neurosci Biobehav Rev.* 2005;29(8):1193-205.
19. Rodgers RJ, Johnson NJ, Cole JC, Dewar CV, Kidd GR, and Kimpson PH. Plus-maze retest profile in mice: importance of initial stages of trail 1 and response to post-trail cholinergic receptor blockade. *Pharmacol Biochem Behav.* 1996;54(1):41-50.

20. Seibenhener ML, and Wooten MC. Use of the Open Field Maze to measure locomotor and anxiety-like behavior in mice. *J Vis Exp.* 2015(96):e52434.
21. Takahashi A, Kato K, Makino J, Shiroishi T, and Koide T. Multivariate analysis of temporal descriptions of open-field behavior in wild-derived mouse strains. *Behav Genet.* 2006;36(5):763-74.
22. Leger M, Quiedeville A, Bouet V, Haelewyn B, Boulouard M, Schumann-Bard P, et al. Object recognition test in mice. *Nat Protoc.* 2013;8(12):2531-7.
23. Lueptow LM. Novel Object Recognition Test for the Investigation of Learning and Memory in Mice. *J Vis Exp.* 2017(126).
24. Miedel CJ, Patton JM, Miedel AN, Miedel ES, and Levenson JM. Assessment of Spontaneous Alternation, Novel Object Recognition and Limb Clasping in Transgenic Mouse Models of Amyloid-beta and Tau Neuropathology. *J Vis Exp.* 2017(123).
25. Rein B, Ma K, and Yan Z. A standardized social preference protocol for measuring social deficits in mouse models of autism. *Nat Protoc.* 2020;15(10):3464-77.
26. Vorhees CV, and Williams MT. Morris water maze: procedures for assessing spatial and related forms of learning and memory. *Nat Protoc.* 2006;1(2):848-58.
27. Angoa-Perez M, Kane MJ, Briggs DI, Francescutti DM, and Kuhn DM. Marble burying and nestlet shredding as tests of repetitive, compulsive-like behaviors in mice. *J Vis Exp.* 2013(82):50978.
28. Richtsmeier JT, Baxter LL, and Reeves RH. Parallels of craniofacial maldevelopment in Down syndrome and Ts65Dn mice. *Dev Dyn.* 2000;217(2):137-45.
29. Maga AM, Tustison NJ, and Avants BB. A population level atlas of Mus musculus craniofacial skeleton and automated image-based shape analysis. *J Anat.* 2017;231(3):433-43.
30. Lele S, and Richtsmeier JT. Euclidean distance matrix analysis: a coordinate-free approach for comparing biological shapes using landmark data. *Am J Phys Anthropol.* 1991;86(3):415-27.
31. Hill CA, Sussan TE, Reeves RH, and Richtsmeier JT. Complex contributions of Ets2 to craniofacial and thymus phenotypes of trisomic "Down syndrome" mice. *Am J Med Genet A.* 2009;149A(10):2158-65.
32. Menegaz RA, Ladd SH, and Organ JM. Craniofacial allometry in the OIM(-/-) mouse model of osteogenesis imperfecta. *FASEB J.* 2020;34(8):10850-9.
33. Dorr AE, Lerch JP, Spring S, Kabani N, and Henkelman RM. High resolution three-dimensional brain atlas using an average magnetic resonance image of 40 adult C57Bl/6J mice. *Neuroimage.* 2008;42(1):60-9.
34. Bankhead P, Loughrey MB, Fernandez JA, Dombrowski Y, McArt DG, Dunne PD, et al. QuPath: Open source software for digital pathology image analysis. *Sci Rep.* 2017;7(1):16878.
35. Schindelin J, Arganda-Carreras I, Frise E, Kaynig V, Longair M, Pietzsch T, et al. Fiji: an open-source platform for biological-image analysis. *Nat Methods.* 2012;9(7):676-82.
36. Lee FHF, Lai TKY, Su P, and Liu F. Altered cortical Cytoarchitecture in the Fmr1 knockout mouse. *Mol Brain.* 2019;12(1):56.
37. Becker LA, Huang B, Bieri G, Ma R, Knowles DA, Jafar-Nejad P, et al. Therapeutic reduction of ataxin-2 extends lifespan and reduces pathology in TDP-43 mice. *Nature.* 2017;544(7650):367-71.
38. Yamashita N, Morita A, Uchida Y, Nakamura F, Usui H, Ohshima T, et al. Regulation of spine development by semaphorin3A through cyclin-dependent kinase 5 phosphorylation of collapsin response mediator protein 1. *J Neurosci.* 2007;27(46):12546-54.

Uncropped/unedited images are presented in Figure 2A, Supplementary Figure 2B-D, Supplementary Figure 4B, and Supplementary Figure 15A.

Full unedited gel for Figure 1E

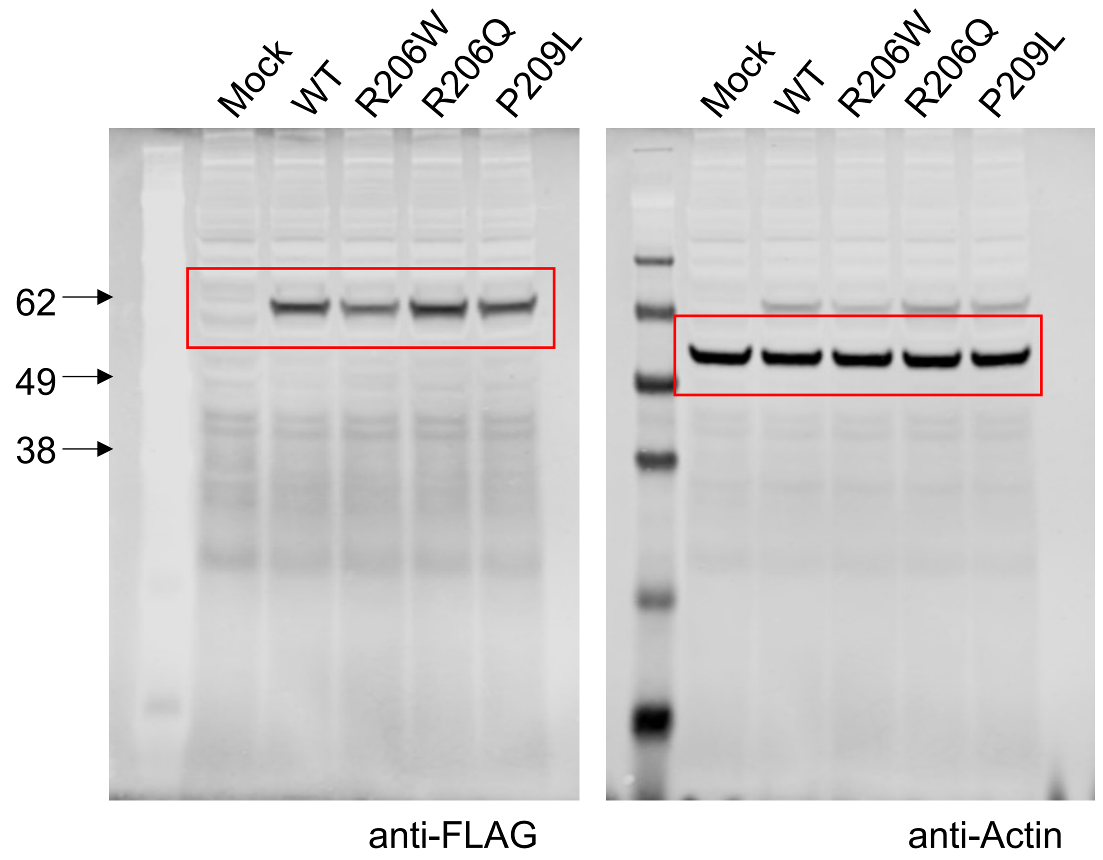

Full unedited gel for Figure 2C

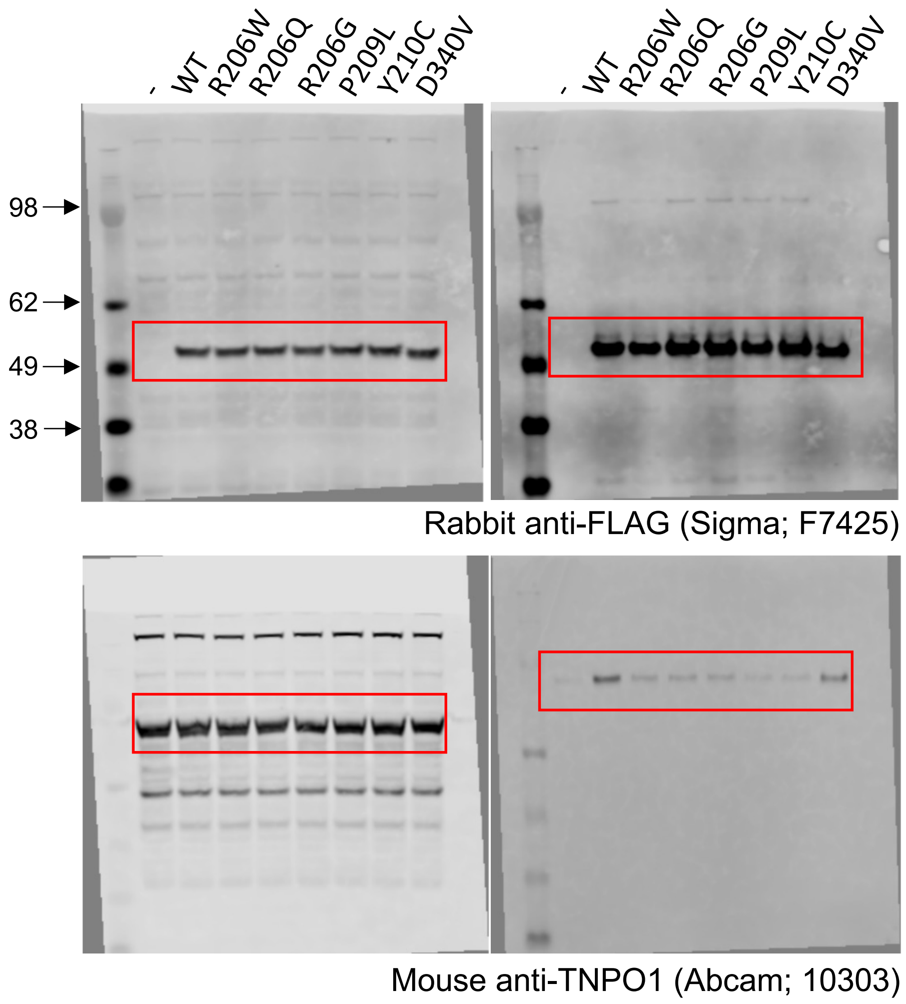

Full unedited gel for Supplementary Figure 14A

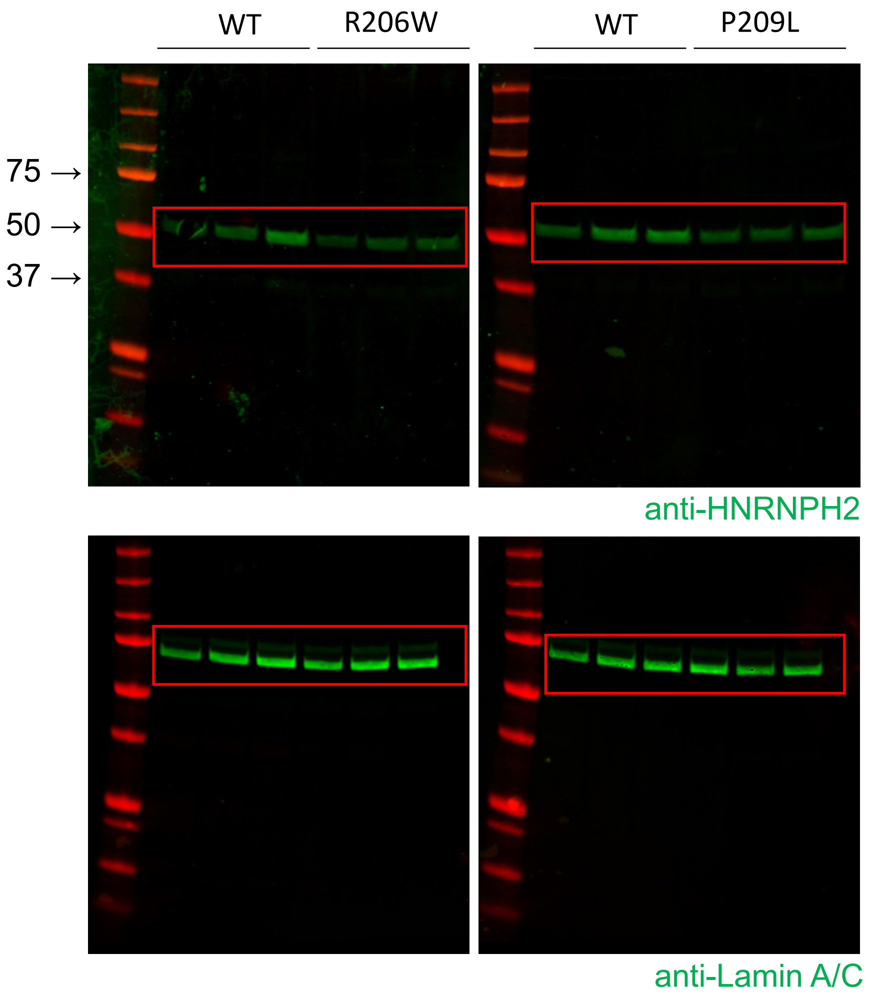

Full unedited gel for Supplementary Figure 4B

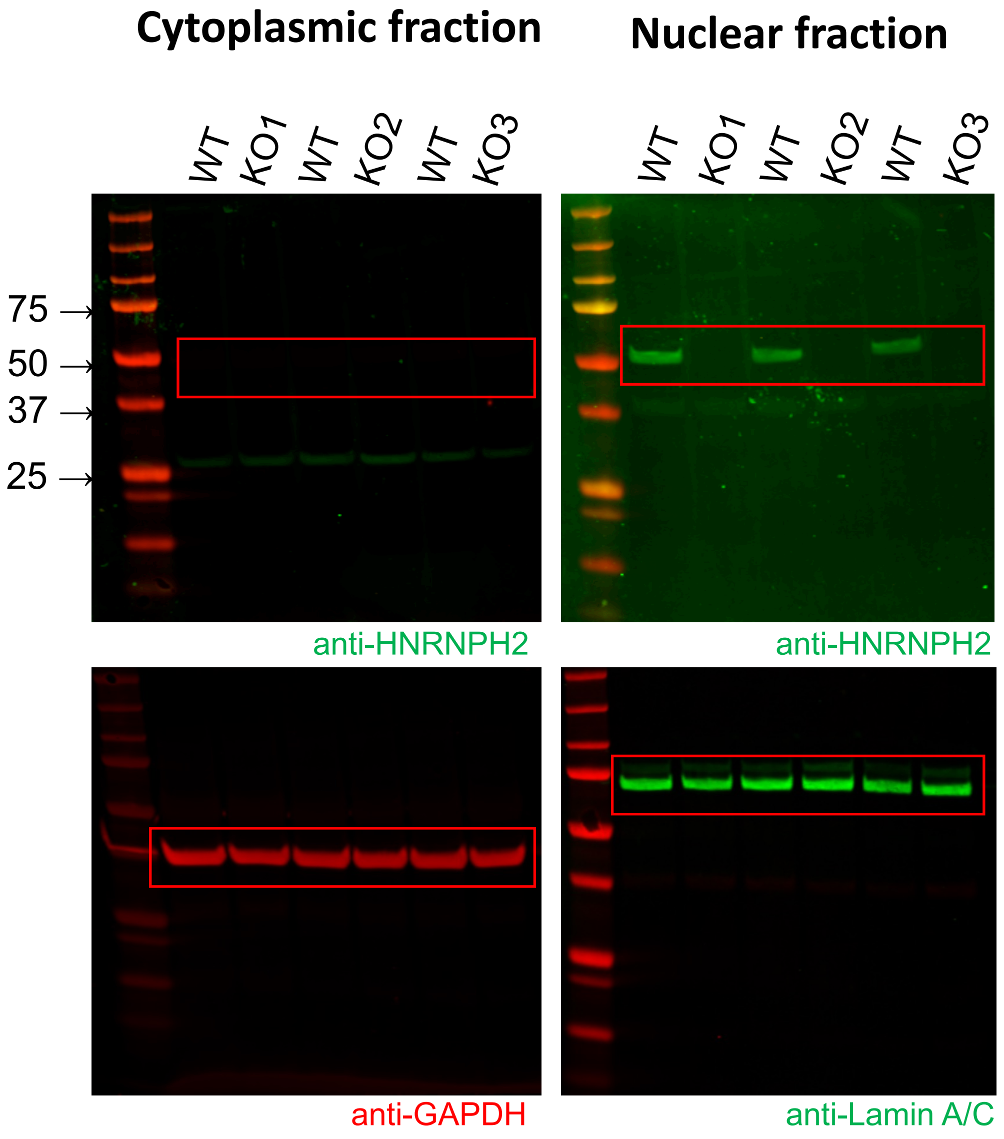

Supplement: Supplemental data [file jci-133-160309-s139.pdf]
